# Assessment of oxygen consumption in response to progressive hypoxia

**DOI:** 10.1371/journal.pone.0208836

**Published:** 2018-12-21

**Authors:** Gary A. Cobbs, James E. Alexander

**Affiliations:** Dept. of Biology, University of Louisville, Louisville, KY, United States of America; University of South Alabama Mitchell Cancer Institute, UNITED STATES

## Abstract

A new method is presented for describing the rate of oxygen consumption in response to progressive hypoxia. The method consists of screening candidate functions describing the relationship between Vo_2_ (oxygen consumption rate) and Po_2_ (ambient oxygen concentration) by testing each for fit to observed data for a single curve and the function that best fits is chosen using lowest AICc value as the criterion. Descriptive statistics are then extracted from the selected function that best describes the pattern present in the curve. Several new descriptive statistics for the pattern of response are proposed which are based on the null model of simple diffusion and no regulation. The method quantifies deviation from the null model at each point on the curve and measures both positive and negative deviation which occur when the curve changes more slowly or more rapidly than the null model predicts, respectively. The new descriptive statistics generalize the traditional one used in the past, the critical oxygen tension (P_c_), and allow interpretation of a variety of shapes of curves which cannot be analyzed with conventional methods. Because the method is descriptive, it does not implicate any specific mechanisms in generating the response. The method is applied to data from 68 animals in 14 different species groups reported in the literature. The overall results suggest the existence of substantial diversity in response types among animals, which requires a more complex description than has traditionally been used.

## Introduction

For many years, measuring tolerance to hypoxia has been a goal of physiologists studying the adaptations of aquatic animals to their environment. The amount of oxygen taken up per unit time (*Vo*_*2*_) typically is plotted as a function of the environmental oxygen levels (oxygen tensions) expressed as the partial pressure of oxygen, *Po*_*2*_. The traditional method of analysis assumes that for a ‘classic conformer’ (a non-regulator), *Vo*_*2*_ declines in direct proportion to declining *Po*_*2*_, and for an oxyregulator, *Vo*_*2*_ remains constant, down to a *Po*_*2*_ level called the critical oxygen tension, P_c_ [1,2]. The range of oxygen tensions in which *Vo*_*2*_ remains constant (from 100% saturation to P_c_) is traditionally referred to as the range of regulation [3]. For an oxyregulator, once P_c_ has been reached, oxygen uptake rates are assumed to decline linearly in proportion with further reductions in ambient oxygen concentrations (the range of conformity, [4,3]. The P_c_ value is at the boundary between the range of regulation and the range of conformity and is used as a measure of regulation intensity. A P_c_ value at full air saturation indicates conformity, while a P_c_ value near that of full air saturation (*Po*_*2*_ = 160 torr or 21.3 kPa) indicates weak regulation and a P_c_ value close to anoxia (*Po*_*2*_ = 0 torr) indicates strong regulation. This model has a single parameter, P_c_, and will be referred to later as the model for a ‘classic regulator’.

A quantitative method commonly used to determine P_c_ is ‘broken stick regression’ [5] where *Vo*_*2*_ is assumed to be constant at its highest value when *Po*_*2*_ ≥ P_c_ at which point *Vo*_*2*_ decreases linearly to zero as *Po*_*2*_ decreases from P_c_ to zero. The broken stick model has been used for estimation of P_c_ even though some data analyzed do not fit the model well. If regulatory ability is not dichotomous (i.e., ‘classic regulation’ versus ‘classic conformity’), and a distinct P_c_ value is not obvious, then additional descriptors of relative regulatory ability are needed to describe the pattern of regulation. Several analytical methods have previously been proposed, most of which involve fitting *Vo*_*2*_ as the response variable of a regression, with *Po*_*2*_ as the explanatory variable. Besides the broken stick linear model described above, various non-linear regression models have been used, and specific regression coefficients used as a measure of the degree of oxygen regulation [1,6,7,5,8,9]. A problem inherent in past attempts to compare the degree of oxygen regulation among aquatic species is that the models used are purely descriptive and the estimated parameters are not direct measures of intensity or pattern of regulation. Many of the problems associated with using P_c_ as the sole descriptor of regulation are discussed by Wood [10]. The method given here is an attempt to give a better and more complete description of observed responses that can be used to characterize different patterns of regulation.

**Fig 1:**
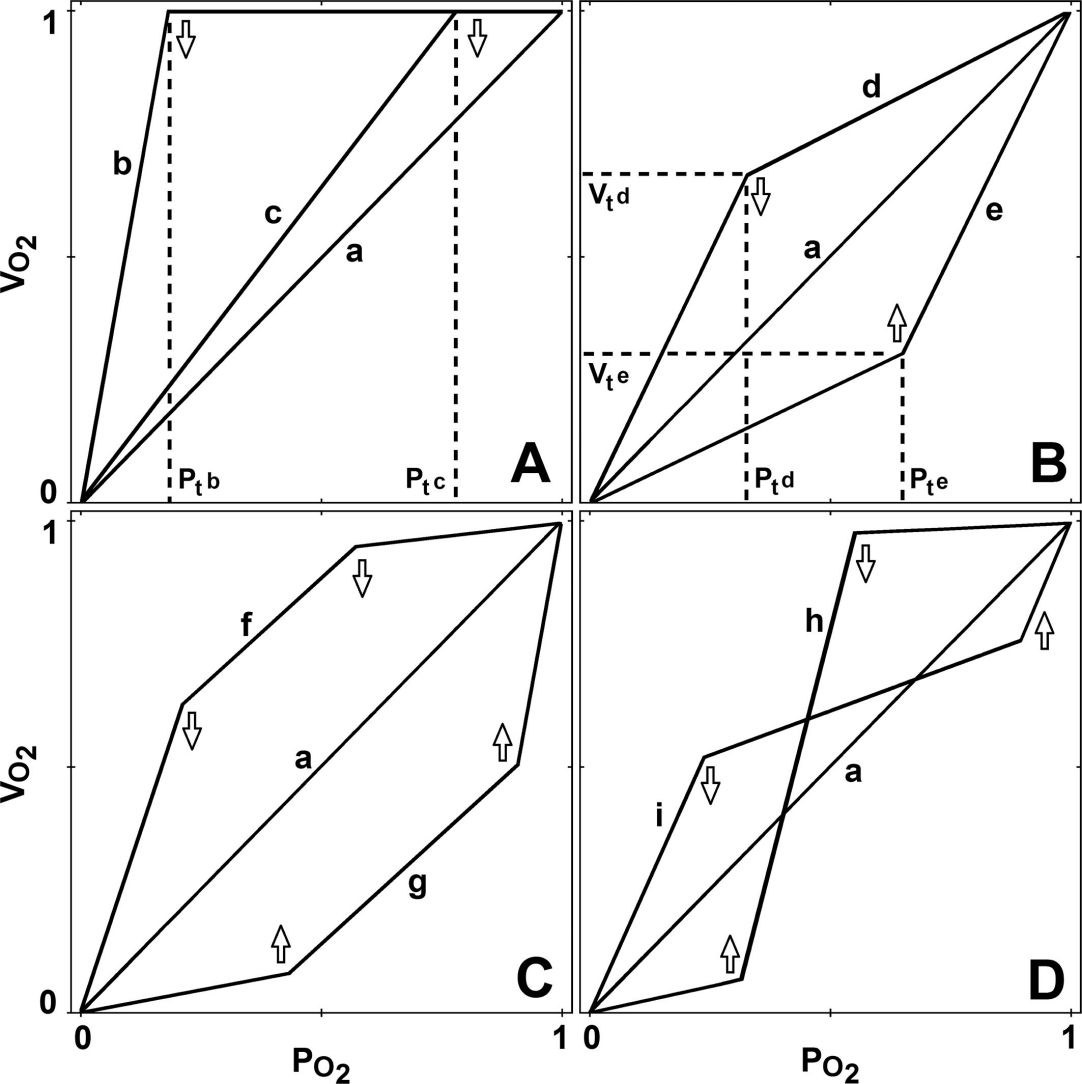
The nine curves (a through i) represent different segmented linear models for progressive hypoxia. Curve **a** represents a classic conformer and is model S1. Curves **b** and **c** represent classic regulation with **b** having more regulation than **c** and are both examples of model S2. Curve **d** represents partial regulation for x between P_t,d_ and 1 and no regulation between 0 and P_t,d_ and is an example of model S3. Curve **e** represents negative regulation for x between P_t,e_ and 1 and no regulation between 0 and P_t,e_ and is an example of model S3. Curves **f**, **g**, **h**, and **i** are all examples of model S4 and each have three linear segments labeled 1, 2, and 3 and all have no regulation in segment 1. Curve **f** has positive regulation in segments 2 and 3. Curve **g** has negative regulation in segments 2 and 3. Curve **h** has negative regulation in segment 2 and positive regulation in segment 3. Curve **i** has positive regulation in segment 2 and negative regulation in segment 3. The symbols ↓ and ↑ denote shift down and shift up threshold points, respectively.

**Fig 2:**
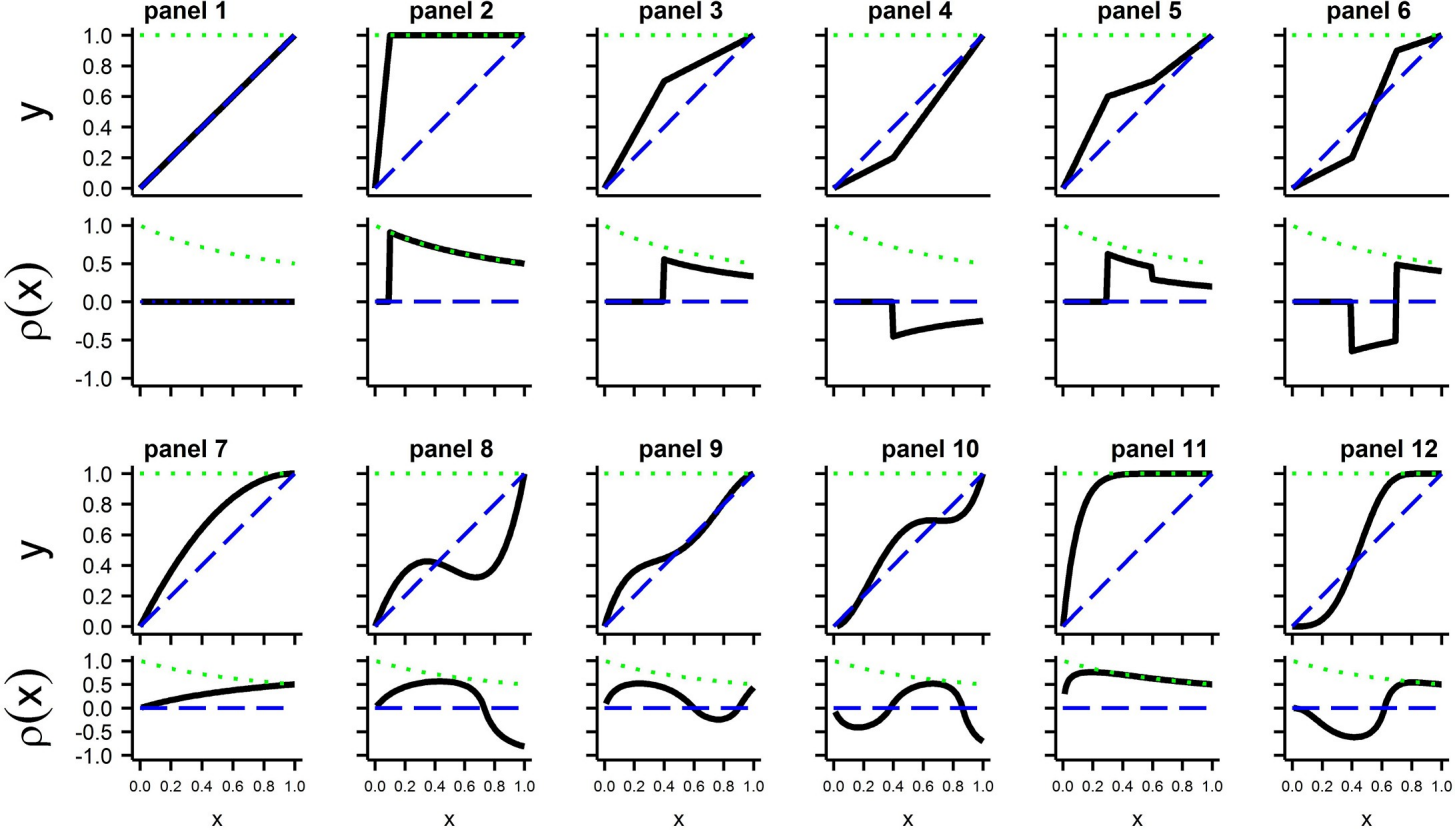
Each panel contains the hypoxia plot on top and the matching regulation profile below. The blue dashed line is the curve for a non-regulator and the green dotted line is the curve for a perfect regulator. The 12 panels contain the following models: (1) S1 (a non-regulator), (2) S2 with P_t_ = 0.1, (3) S3 with P_t_ = 0.4, V_t_ = 0.7, (4) S3 with P_t_ = 0.4, V_t_ = 0.2, (5) S4 with P_t1_ = 0.3, V_t1_ = 0.6, P_t2_ = 0.6, V_t2_ = 0.7, (6) S4 with P_t1_ = 0.4, V_t1_ = 0.2, P_t2_ = 0.7, V_t2_ = 0.9, (7) Polynomial with b_0_ = 0, b_1_ = 2, b_2_ = -0.99, b_3_ = -0.01, b_4_ = 0, (8) Polynomial with b_0_ = 0, b_1_ = 2.6, b_2_ = -4, b_3_ = -1.1, b_4_ = 3.5, (9) Polynomial with b_0_ = 0, b_1_ = 3.333, b_2_ = -10.222, b_3_ = 14.222, b_4_ = -6.333, (10) Polynomial with b_0_ = 0, b_1_ = 0.1, b_2_ = 9.2, b_3_ = -17.5, b_4_ = 9.4, (11) C2 with P_p_ = 0.1, (12) C3 with P_p_ = 0.5, V_p_ = 0.3.

There are recurring patterns in data that deviate from the traditional model for regulation. For some animals, *Vo*_*2*_ initially declines linearly with increasing hypoxia until a threshold *Po*_*2*_ level is reached, at which regulation ceases and *Vo*_*2*_ decreases to zero (Fig 1B curve d). Oxygen uptake rates for some species initially increase under increasing hypoxia, or increase at some intermediate *Po*_*2*_ level, presumably due to increased ventilation rates, increased circulatory fluid perfusion rates, or due to movement. In other cases, *Vo*_*2*_ drops initially as *Po*_*2*_ declines, then *Vo*_*2*_ levels off at some lower level over a range of *Po*_*2*_ and eventually reaches a threshold *Po*_*2*_ value at which all regulation stops (Fig 1D curve i). Finally, for many species described as regulators in the published literature, an abrupt transition point (P_c_) is not found and instead of a single point, a range of P_c_ values are reported to describe a single hypoxia curve [11]. A thorough search of the literature showed that curves similar to the patterns shown in Figs 1 and 2 are present throughout the animal kingdom.

The prevalence of poor fit to the traditional regression model [12] emphasizes the need for more versatile methods to describe the relationship between *Vo*_*2*_ and *Po*_*2*_. An integrative method for estimating and comparing overall oxygen regulatory capacity was developed to measure the amount of oxygen regulation [13] called the Regulation Value (R). In this method, the metabolic rate (*Vo*_*2*_) of an individual is obtained during a progressive hypoxia experiment over the entire *Po*_*2*_ gradient (from 100% to 0% saturation). The metabolic rate is standardized as a percentage of the highest *Vo*_*2*_ value recorded for the individual during the run (*Vo*_*2*_ ranging from 0 to 100%). R is the area under the *Vo*_*2*_*-Po*_*2*_ curve and expressed as a percentage of the total possible area. R thus can range from 0 to 100%, where a regulator will have a value exceeding 50% and a ‘classic conformer’ over the entire range of *Po*_*2*_ will have a value of 50%. Organisms whose R value is below 50% are described as ‘hypoxia sensitive’ [13]. Mueller and Seymour [8] later independently derived a similar metric, which they called the Regulation Index (RI). These integrated metrics (R and RI) have been used for comparative studies providing a single value which summarizes the overall respiratory responses of organisms over the entire range of environmental oxygen levels, from full air saturation to anoxia [13,8]. While R and RI are useful measures of the overall metabolic response over the full range of oxygen tensions (full air saturation to anoxia), they do not provide information about critical oxygen tensions at which organisms shift their metabolic response, which is of importance for many comparative studies [8].

Here, we show the majority of data obtained from experiments on hypoxia tolerance reflect a greater variety of more complex relationships between *Vo*_*2*_ and *Po*_*2*_ than the traditional models describe. We propose new analytical methods for the interpretation of data from such experiments. New descriptive statistics are proposed that provide more complete descriptions of the diversity in observed patterns of oxygen tolerance and quantify intensity of response at specific *Po*_*2*_ values as well as total amount of regulation over the entire range of the curve. The new methods include the broken stick model as a special case and may be regarded as a generalization of current methods. The absence of a mechanistic model describing the effect of decreasing *Po*_*2*_ on *Vo*_*2*_ necessitates the use of purely descriptive methods to compute statistics characterizing intensity and pattern of hypoxia tolerance. The descriptive statistics proposed here may be used to compare responses among different types of organisms, or to compare the responses of a given species under different conditions. They are likely to be useful for the investigation of the mechanisms of regulation oxygen consumption. Methods for assessing hypoxia tolerance such as those presented here can predict the biological consequences of increasing levels of hypoxia currently occurring throughout the world’s oceans [14,15].

To date, no broad quantitative generalizations between ecological or evolutionary patterns and oxyregulation have been identified for aquatic animals [1,6,4,7,16,17,11,18,3]. In these studies, animals were categorized as either oxyregulators or oxyconformers, although the validity of a dichotomy of regulation and conformity has been questioned in the past [7,17]. A number of authors have suggested a continuum of regulation, ranging from strict regulation to conformity; many species appear to lie along such a continuum [7,19,20,13,21]. The method presented here is more versatile than those previously available for describing and studying patterns of regulation in response to hypoxia and may be used to determine if a continuum of regulation types exists in some organisms.

## Materials and methods

Functions are used to model the relationship between *Vo*_*2*_ and *Po*_*2*_ relative to their values at *Po*_*2*_ = 100%. Define *x = Po*_*2*_ */100* and y = *Vo*_*2*_ /*(Vo*_*2*_ at *Po*_*2*_ = 100) which are standardized oxygen levels of the environment and standardized oxygen consumption levels of the organism, respectively. Any function *y = f(x)* giving a good description of the relationship between x and y may be used to extract statistics describing patterns of response. The method involves choosing the best *f(x)* from a set of candidate functions. Statistics which describe the pattern of tolerance to progressive hypoxia are then extracted from the selected *f(x)*. Different choices for *f(x)* may be viewed as different ways of smoothing the data before extracting descriptive statistics from the smoothed data. Functions involving parameters are used for *f(x)* rather than other types of smoothing techniques because they may be compared by ΔAICc and evidence ratio (ER) values [22,23,24,25]. Descriptive statistics proposed here are described below and the candidate selections used for *f(x)* are described in the section titled ‘Selections for *f(x)*’.

### Descriptive statistics

A new descriptive statistic, P_t_, is a threshold value for x at which there is an abrupt change or discontinuity in the slope of *f(x)*. If *f(x)* is a segmented linear function, the point/s (*P*_*t*_, *f(P*_*t*_*)*) is/are points at which two linear line segments join. If *f(x)* is a linear segmented model with two line segments, (0,0)→(P_t_,1) and (P_t_,1)→(1,1), then P_t_ is the same as the parameter P_c_ for the model of a ‘classic regulator’discussed in the introduction. If *f(x)* is continuous, threshold points are defined using curvature and P_t_ is an x value at which there is a relative minimum or relative maximum in curvature and for which the absolute amount of curvature at x = P_t_ exceeds a defined limit, Kcutoff. For a continuous curve defined by *f(x)*, curvature at x is given by *κ(x)* = *f''(x)*/[1+{*f'(x)*}^2^]^-3/2^ where *f'(x)* and *f''(x)* denote first and second derivatives of *f(x)*, respectively. If *κ(x)* is a relative minimum or relative maximum and |*κ(x)*|>Kcutoff, then x is denoted a threshold value, *P*_*t*_. Kcutoff is a value for curvature that ensures the change in slope at P_t_ is sufficiently larger than in adjacent regions that it may be considered a threshold value. All analyses presented here used Kcutoff = 1.5. A P_t_ value may be mechanistically interpreted as a value of x at which the organism undergoes an abrupt change in intensity and/or direction of regulation though mechanisms other than active regulation might also cause a threshold to be present in a hypoxia curve.

Another descriptive statistic, V_t_ = *f*(P_t_), may be mechanistically interpreted as a minimal value of y an organism will tolerate before substantially changing the pattern of regulation. Introducing the statistic, V_t_, allows description of more complex patterns of hypoxia tolerance than the model of ‘classic regulation’ is capable of (see Fig 1B curves d and e).

While many organisms have hypoxia curves with a single threshold point, it is possible for a single curve to have two or more threshold points. Different threshold points will be denoted with subscripts, *i*.*e*., (P_t,1_,V_t,1_), (P_t,2_,V_t,2_), and so on. None of the hypoxia curves analyzed here had more than two threshold points in the best fitting model, and it is likely that more than two threshold points are rarely needed to adequately describe data. It is also possible for a hypoxia curve to show regulation and have no threshold points (see Fig 2 panel 7 top). Such situations can be described only with a continuous selection for *f(x)*; the presence or absence of threshold points will depend on the value selected for Kcutoff.

#### Measures of instantaneous regulation

While threshold points are useful for describing the shape of some curves, they may not be present in a hypoxia curve for which regulation is present. Even if threshold points are present, they may not capture important aspects of the pattern of response. Threshold points are not measures of direction or intensity of regulation, but are simply values at which there is greater change in trend of *y* than in adjacent areas in the curve. A measure of instantaneous regulation quantifies the intensity of regulation at a single point, (x, *f(x)*). In order to describe the intensity of instantaneous regulation, a model for the absence of instantaneous regulation is required so that regulation can be defined as deviation from no regulation. Fick’s first law of diffusion applied to diffusion across a single membrane will be used as the null model for absence of change due to regulation or similar effects. In this discussion, for simplicity, the law of diffusion is applied to the standardized variables x and y rather than *Po*_*2*_ and *Vo*_*2*_, but because x and y are simply scale transformations of *Po*_*2*_ and *Vo*_*2*_, respectively, the same results could be obtained by applying the law of diffusion to the unstandardized variables. Assume that due to metabolic processes of an animal, the oxygen concentration inside the animal is much lower than in the surrounding aqueous environment, x. Also assume oxygen entering the animal is immediately metabolized or bound to temporary storage structures preventing it from leaving the animal by diffusion. In this situation Fick’s first law states the rate of diffusion of oxygen into the organism is proportional to x. Under this null model the relationship between y and x is y = kx where k is a constant proportional to the diffusion coefficient for oxygen. From y = kx we obtain k = y/x and dy/dx = k and equating these two gives dy/dx = y/x. The general null model for the relationship of y to x is then dy/dx = y/x. A special case of the general null model occurs if the line passes through the point (1,1), then k = 1 and the line is y = x.

A function *f(x)* is in agreement with the general null model at x if *fʹ(x) = f(x)/x*. We therefore propose an instantaneous measure of regulation as the amount of deviation from the null model at x which is

$$\rho (x)=\frac{f(x)}{x}-f^{`}(x)$$ (1)

which for 0≤x≤1 has range -∞ <*ρ(x)*< ∞. In Eq (1), *f(x)*/x is the slope that is expected under the null model at x and *f* ʹ*(x)* is the slope of *f(x)* at x and *ρ(x)* is the difference between these two slopes. If *ρ(x)* <0, its value is the negative of the excess of slope above that expected under the null model. If *ρ(x)* = 0, the slope is equal to that expected under the null model. If *ρ(x)* >0, its value is the negative of the deficit of slope below that expected under the null model. Subsequently we will refer to *ρ(x)* as a measure of regulation but it might also be sensitive to effects that some may not consider to be regulation. Because all mechanisms producing a hypoxia response curve are not known for most animals, there is no unequivocal mechanistic interpretation of a hypoxia curve or for *ρ(x)*. We propose *ρ(x)* as a general measure of any type of deviation from the null model which includes active regulation and may also include passive and other types of processes that may not be considered as regulatory in nature. The null model we use here in the definition of *ρ(x)* has been used in hypoxia studies for decades.

A classic regulator may be used as a reference when measuring intensity of regulation with *ρ(x)*. For a ‘classic regulator’ with parameter P_t_, as described in the introduction, *f(x)* = x/P_t_ for x<P_t_ and *f(x)* = 1 for x≥P_t_. Using Eq (1) shows that for a ‘classic regulator’ *ρ(x)* = 0 for x<P_t_ and *ρ(x)* = x^-1^ for x≥P_t_ (see Fig 2 panel 2). Interpretations of values of *ρ(x)* are given below.

∙ If *ρ(x)* < 0 then ‘negative regulation’ is occurring at x.

∙ If *ρ(x)* = 0 then ‘no regulation’ is occurring at x.

∙ If 0 < *ρ(x)* < x^-1^ then ‘partial positive regulation’ is occurring at x

∙ If *ρ(x)* = x^-1^ then ‘perfect positive regulation’ is occurring at x.

∙ If *ρ(x)* > x^-1^ then ‘positive over-regulation’ is occurring at x.

A plot of *ρ(x)* versus x will be called the regulation profile and describes the direction, intensity, and pattern of regulation over values of x. Three statistics describing the pattern of response extracted from *ρ(x)* are P_c-min_, P_c-zero_, and P_c-max_, which are the values of x for which *ρ(*x*)* is at its minimum, zero, and its maximum, respectively. For some choices for *f(x)* such as segmented linear functions (which includes a perfect regulator), *ρ(x)* may be zero over a range of x values. To make P_c-zero_ a unique value, it is defined as a value of x for which *ρ(x)* = 0 and for which there is transition between any two of the three categories of regulation, *ρ(x)<0*, *ρ(x) = 0*, *ρ(x*)>0. This occurs if *ρ(P*_*c-zero*_*)* = 0 and *ρ(P*_*c-zero*_*+δ)*≠0 for |δ|≳0. For a ‘perfect regulator’, P_c_ = P_t_ = P_c-min_ = P_cmax_ = P_c-zero_, however, for *f(x)* more complex than the traditional ‘classic regulator’, P_c_ may be undefined and the values for P_t_, P_c-min_, P_cmax_, and P_c-zero_ may all be different from one another. Examples of regulation profiles for a variety of selections for *f(x)* are given in lower part of each panel of Fig 2 and will be described in more detail in the section below titled ‘selections for *f(x)’*.

#### Measures of total regulation

Total directional regulation estimated from the interval L_1_<x<L_2_, where 0≤L_1_<L_2_≤1, is defined as

$$\tau_{dir}(L_{1},L_{2})=\left(\frac{1}{L_{2}-L_{1}}\right)\int_{L_{1}}^{L_{2}}\rho (x)dx$$ (2)

and is the mean of *ρ(x)* over L_1_<x<L_2_. For a ‘classic regulator’ with parameter P_t_, τ_dir_(0,1) = -ln(P_t_). Similarly, total absolute regulation estimated from the interval L_1_<x<L_2_ is defined as

$$\tau_{dir}(L_{1},L_{2})=\left(\frac{1}{L_{2}-L_{1}}\right)\int_{L_{1}}^{L_{2}}|\rho (x)|dx$$ (3)

and is the mean of |*ρ(x)|* over L_1_<x<L_2_. Now define total positive regulation over the interval L_1_<x<L_2_ as

$$T_{pos}(L_{1},L_{2})=\left(\frac{1}{L_{2}-L_{1}}\right)\int_{L_{1}}^{L_{2}}I_{\rho (x)>0}\rho (x)dx\;where\;I_{S}(x)=\left\{ \begin{array}{c} 1\;if\;x\in S\\ 0\;if\;x\notin S \end{array}\right.$$ (4)

and total negative regulation over the interval L_1_<x<L_2_ as

$$T_{neg}(L_{1},L_{2})=\left(\frac{1}{L_{2}-L_{1}}\right)\int_{L_{1}}^{L_{2}}I_{\rho (x)<0}\rho (x)dx\;where\;I_{S}(x)=\left\{ \begin{array}{c} 1\;if\;x\in S\\ 0\;if\;x\notin S \end{array}\right.$$ (5)

These may be computed from τ_dir_(L_1_,L_2_) and τ_abs_(L_1_,L_2_) as

$$T_{pos}(L_{1},L_{2})=\frac{\tau_{abs}(L_{1},L_{2})+\tau_{dir}(L_{1},L_{2})}{2}$$ (6)

and

$$T_{pos}(L_{1},L_{2})=\frac{\tau_{abs}(L_{1},L_{2})-\tau_{dir}(L_{1},L_{2})}{2}$$ (7)

and are the mean positive and mean negative regulation over L_1_<x<L_2_, respectively. The ranges of T_pos_ (L_1_,L_2_) and T_neg_ (L_1_,L_2_) are 0≤T_pos_ (L_1_,L_2_) ≤ +∞ and 0≤T_neg_ (L_1_,L_2_) ≤ +∞, respectively. Values of T_pos_ (L_1_,L_2_) or T_neg_ (L_1_,L_2_) near 0 indicate low amount of total regulation and higher values indicate greater amount of total regulation. A ‘classic regulator’ may be used as a reference when interpreting these measures of total regulation. For a ‘classic regulator’ with parameter P_t_ >0 (same as P_c_>0), T_pos_(0,1) = -ln(P_t_) and T_neg_ (0,1) = 0. If an animal that is not a ‘classic regulator’ has T_pos_ amount of positive regulation then exp(-T_pos_) gives an effective P_t_, or equivalently effective P_c_, which is the P_t_ value that gives the same amount of total positive regulation for a ‘classic regulator’.

The method presented here assumes any chosen *f(x)* and its corresponding *ρ(x)* are perfect descriptors of the true relationship with x over the entire range of x, 0≤x≤1. If *f(x)* only approximates the true relationship, as is always the case, there is a problem in accurately describing the hypoxia curve and its response profile when x is close to 0. For example, if ρ(x) has a singularity at x = 0 then its behavior near 0 may be biologically unreasonable, but reasonable further from zero. Another reason for inaccuracy near x = 0 is the non-existence of data points near and below x = 0 which compromises the smoothing ability of a fitted *f(x)* near x = 0. This problem could be rectified somewhat by making many measurements of y near x = 0, but this is almost never done in practice. The best *f(x)* is chosen based on its fit to the entire hypoxia curve and its shape when x is close to 0 may be of questionable accuracy because there are few or no data points in this region other than at (0,0). To minimize this effect, the value of *ρ(x)* in the regulation profile is presented only in the range L_1_≤x≤L_2_ where L_1_ is chosen as the second smallest x in the data and L_2_ is chosen as the second largest x in the data. This definition for L_1_ and L_2_ allows the data to restrict the range for estimation of *ρ(x)*. Computation of values for τ_dir_ and τ_abs_ are also adjusted using these same choices for L_1_ and L_2_. T_pos_, and T_neg_ are then computed from the adjusted τ_dir_ and τ_abs_ as previously described.

#### Selections for *f(x)*

Choices used for initial candidate functions to be screened are described here. Other choices could be added to this list, however, in this study one of the choices presented here always gave a good fit to the data. The functions, *f(x)*, for each model are given in Table 1. The formula for the regulation profile, *ρ(x)*, τ_dir_, and τ_abs_ for each model are given in Tables A-C in S1 Appendix. The selections are in two categories, segmented linear models and continuous models, and are described below.

**Table 1:** Formulae for the seven choices for *f(x)*.

name	y = *f(x)* for 0≤ x ≤ 1, for S2, S3, and S4 below: if x∈s, I_s_(x) = 1 otherwise I_s_(x) = 0
S1	y = x
S2	$y=I_{x<P_{t}}(x)\left(\frac{1}{P_{t}}\right)x+I_{x\geq <P_{t}}(x)$
S3	$y=I_{x<P_{t}}(x)\left(\frac{V_{t}}{P_{t}}\right)x+I_{P_{t}\leq x\leq 1}(x)\left[V_{t}+(x-P_{t})(\frac{1-V_{t}}{1-P_{t}})\right]$
S4	$y=I_{x<P_{t,1}}(x)\left(\frac{V_{t,1}}{P_{t,1}}\right)x+I_{P_{t,1}\leq x\leq P_{t,2}}(x)\left[V_{t,1}+(x-P_{t,1})(\frac{V_{t,2}-V_{t,1}}{P_{t,2}-P_{t,1}})\right]+I_{P_{t,2}\leq x\leq 1}(x)\left[V_{t,2}+(x-P_{t,2})(\frac{1-V_{t,2}}{{1-P}_{t,2}})\right]$
C2	$y=1-{\left(1-x\right)}^{\left(\frac{1}{P_{p}}\right)}\;where\;0<Pp$
C3	$y=1-{\left(1-x^{\left(\frac{1}{V_{p}}\right)}\right)}^{\left(\frac{1}{P_{p}}\right)}where\;0<Pp\;and\;0<Vp$
MP	y=∑0kbixifori=1,2,…,kconstrainedsodydx>0for0≤x≤1

#### Segmented linear models

The relationship between y and x, *y = f(x)*, is a segmented linear function for 0≤x≤1. Segmented linear functions with up to three segments are used here and model parameters are the coordinates of the points where line segments join. The four segmented linear models use here are called S1, S2, S3, and S4 and are described below.

**Model S1 (1 segment)**: The ends of the single linear segment are at (0,0) and (1,1) (see curve **a** of all panels of Fig 1). Model S1 has *ρ(x)* = 0 for all x and thus depicts the null model which has no regulation of any type. Model S1 may also be referred to as the model of a ‘classic conformer’. The hypoxia plot and regulation profile are given in the upper and lower plots, respectively, of panel 1 of Fig 2.

**Model S2 (2 segments):** The ends of the two linear segments lie at (0,0)→(P_t_, 1) and (P_t_, 1)→(1,1), where 0≤P_t_≤1, and the two segments are joined at the point (P_t_, 1), the critical point (see Fig 1A curves b and c). This is the conventional model of a ‘classic regulator’ described in the introduction and has been used extensively for interpretation of hypoxia curves. Model S2 has *ρ(x)* = 0 when x≤P_t_ and *ρ(x)* = x^-1^ P_t_≤x. Model S2 simplifies to model S1 if P_t_ = 1. Sample hypoxia plot and matching regulation profile are given in upper and lower plots, respectively, of panel 2 of Fig 2.

**Model S3 (2 segments)**: Coordinates of the ends of the two linear segments are (0,0)→(P_t_,V_t_) and (P_t_,V_t_)→(1,1), where 0≤P_t_≤1 and 0≤V_t_≤1 (see Fig 1B curves d and e). The two segments join at the point (P_t_,V_t_) which is a critical point. If x<P_t_, there is no regulation since *ρ(x)* = 0. If x≥P_t_ and V_t_>P_t_, only positive regulation occurs. If x≥P_t_ and V_t_<P_t_, only negative regulation occurs. If V_t_ = 1 model S3 simplifies to model S2 and if V_t_ = 1 and P_t_ = 1 model S3 simplifies to model S1. If V_t_ = P_t_, there is no point of discontinuity and model S3 simplifies to model S1. Sample hypoxia plots and matching regulation profiles are given in upper and lower plots, respectively, of panels 3 and 4 of Fig 2.

**Model S4 (3 segments)**: Ends of the three linear segments lie at (0,0)→(P_t1_,V_t1_), (P_t1_,V_t1_)→(P_t2_,V_t2_), (P_t2_,V_t2_)→(1,1), where 0≤P_t1_≤P_t2_≤1 and 0≤V_t1_≤1 and 0≤V_t2_≤1 (see Fig 1 panel C curves f and g and panel D curves h and i). If (P_c2_,V_c2_) = (1,1) model S4 simplifies to model S3. If (P_c2_,V_c2_) = (1,1) and (P_c1_,V_c1_) = (P_c1_,1) model S4 simplifies to model S2. If (P_c2_,V_c2_) = (1,1) and (P_c1_,V_c1_) = (1,1) model S4 simplifies to model S1. Model S4 always has two threshold points at (P_t1_,V_t1_), (P_t2_,V_t2_). The threshold value P_t1_ is always a P_c-zero_ value. For S4 models sample hypoxia plot and matching regulation profile are given in upper and lower plots, respectively, of panels 5 and 6 of Fig 2.

#### Continuous models

Here *f(x)* is a continuous function for 0≤x≤1. Three different selections for continuous *f(x)* models denoted MP, C3 and C2 are described below.

**Model MP (monotone polynomial):** Model MP is a polynomial in x of arbitrary degree and monotonically increasing in the interval 0≤x≤1. Monotone polynomials of degree 1 through 7 were each fit to a curve and the one with the lowest AICc value was taken as the best monotone polynomial model (MP). Model MP is able to fit many shapes of curves. If the only non-zero coefficient is b_1_, and b_1_ = 1, then model MP simplifies to model S1. Sample hypoxia plots and matching regulation profiles for a variety of selections of coefficient values are given in upper and lower plots, respectively, of panels 7,9, and 10 of Fig 2.

**Model C3 (double exponent model):** The model C3 function (see Table 1) is a non-linear function that can assume a wide variety of shapes for x in (0,1) and has parameters P_p_ and V_p_. Model C3 is a monotonically increasing function with *f(0)* = 0 and *f(1)* = 1. It was chosen because it has some properties in common with models S1, S2, and S3. If P_p_ = 1 and V_p_ = 1, model C3 reduces to model S1 (no regulation). A sample hypoxia plot and matching regulation profile are given in upper and lower plots of panel 12 Fig 2.

**Model C2 (a single exponent model):** Model C2 is the special case of model C3 with parameter V_p_ = 1 and 0<P_p_ (see Table 1) and is a monotonically increasing function. If P_p_<1 then model C2 gives only positive regulation. If P_p_ = 1 then model C2 gives no regulation and becomes model S1. If P_p_>1 then model C2 gives only negative regulation. A sample hypoxia plot and matching regulation profile are given in upper and lower plots of panel 11 of Fig 2.

#### Fitting models to data

Models S1, S2, C2 and C3 were fit to data using the nlsLM function in R. Models S3 and S4 were fit using the segmented function of the segmented package of R by searching a net of 10,000 start vectors for the one that gave the best fit. Some curves could not be fit to model S4 because the best fit had P_t2_ = 1 and V_t2_ = 1 which makes model S4 become model S3. Some curves could not be fit to model S3 because the best fit had P_t_ = 1 or V_t_ = 1 which makes model S3 become model S1 or S2. The MP model was fit using the monopol function of the monopoly package of R. Values of x which gave minimum or maximum curvature or minimum, maximum, or zeros for *ρ*(x) for models C2, C3 and MP were found numerically. For a particular curve, the seven fitted models (S1,S2,S3,S4,C2,C3,MP) are compared using AICc value, which is AIC corrected for small sample size [26,27]. A lower value of AICc indicates a better fitting model and the model with the lowest AICc value is taken as the best among the models tested. The regulation profile, threshold points, critical points, T_pos_, T_neg_ and R for models S1, S2, S3, and S4 were obtained with analytic formulae given in S1 Appendix or determined numerically. For models C2, C3 and MP, values for T_pos_ and T_neg_ were computed using the integrate function of R.

Because the AICc value is a measure of quality of fit, models with similar AICc values are similar in their explanation of the data. The difference in AICc values for two models, ΔAICc, is a measure of difference in their quality of fit. Burnham and Anderson [24] (p. 70) suggest a ΔAICc of 10 or more indicates very substantial difference between two models, a ΔAICc less than 2 indicates essentially no difference between two models, and a ΔAICc between 4 and 7 implies some difference between two models. Evidence ratios (ER) are the ratio of the probabilities of two models for a given set of data and are computed from the ΔAICc values. Here, the ER is computed for an alternative model, with the best model being the reference standard and ER is the fold increase in probability of the best model over the alternative model. Consider the best fitting model for a particular animal. If none of the other models when applied to that animal give an ER less than 15, the best model will be denoted the ‘uniquely best model’ for that animal. If another model gives an ER<15, the best model will then be denoted the ‘best model’ and any alternative model that gives an ER value less than 15 will be called an ‘equivalent alternative model’.

### Data set

Hypoxia tolerance data for 68 individual organisms in 16 groups were obtained from the literature. The species, number of individuals, and source of the data are given in Table D in S1 Appendix. Animals were selected from published literature using the following criteria. First, each data point is the oxygen uptake response of a single animal to a progressive decrease in Po_2_ in a closed respirometer system, where the metabolic activities of the animal decreased the oxygen level over time. Second, the response was depicted with at least ten (Po_2_, Vo_2_) measurement points with Po_2_ values ranging from >80% oxygen saturation to <20% saturation.

## Results

### Examples of animals with best fitting models S1, S2, S3, S4, C3, and MP

**Fig 3:**
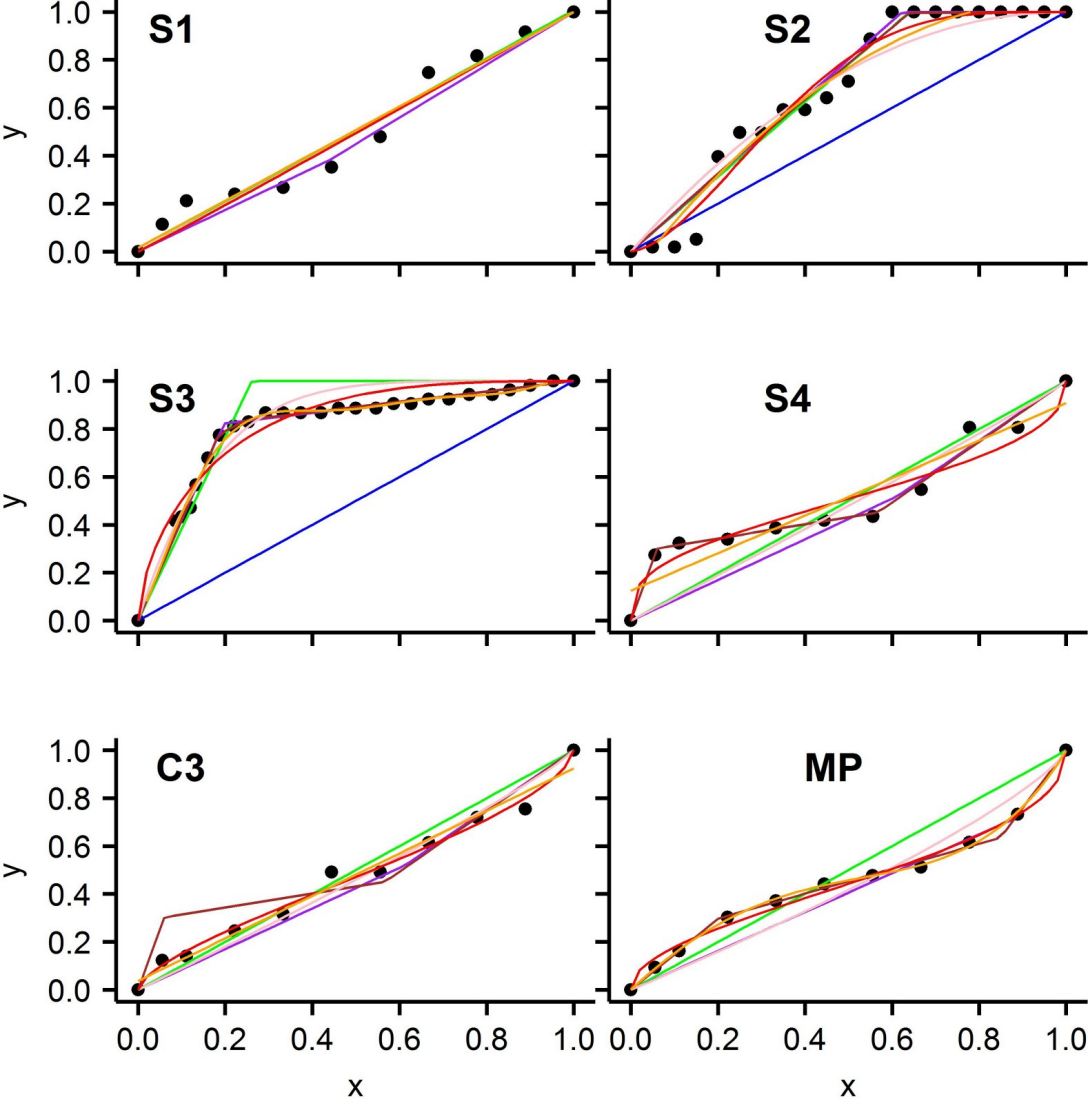
Results of fitting all 7 models to each of six selected animals. Each panes is a separate animal and the name of the best fitting model for the animal is given at the top of the panel. Black dots are the observed data points. Solid lines are: blue = S1, green = S2, purple = S3, brown = S4, pink = C2, red = C3, orange = MP.

**Fig 4:**
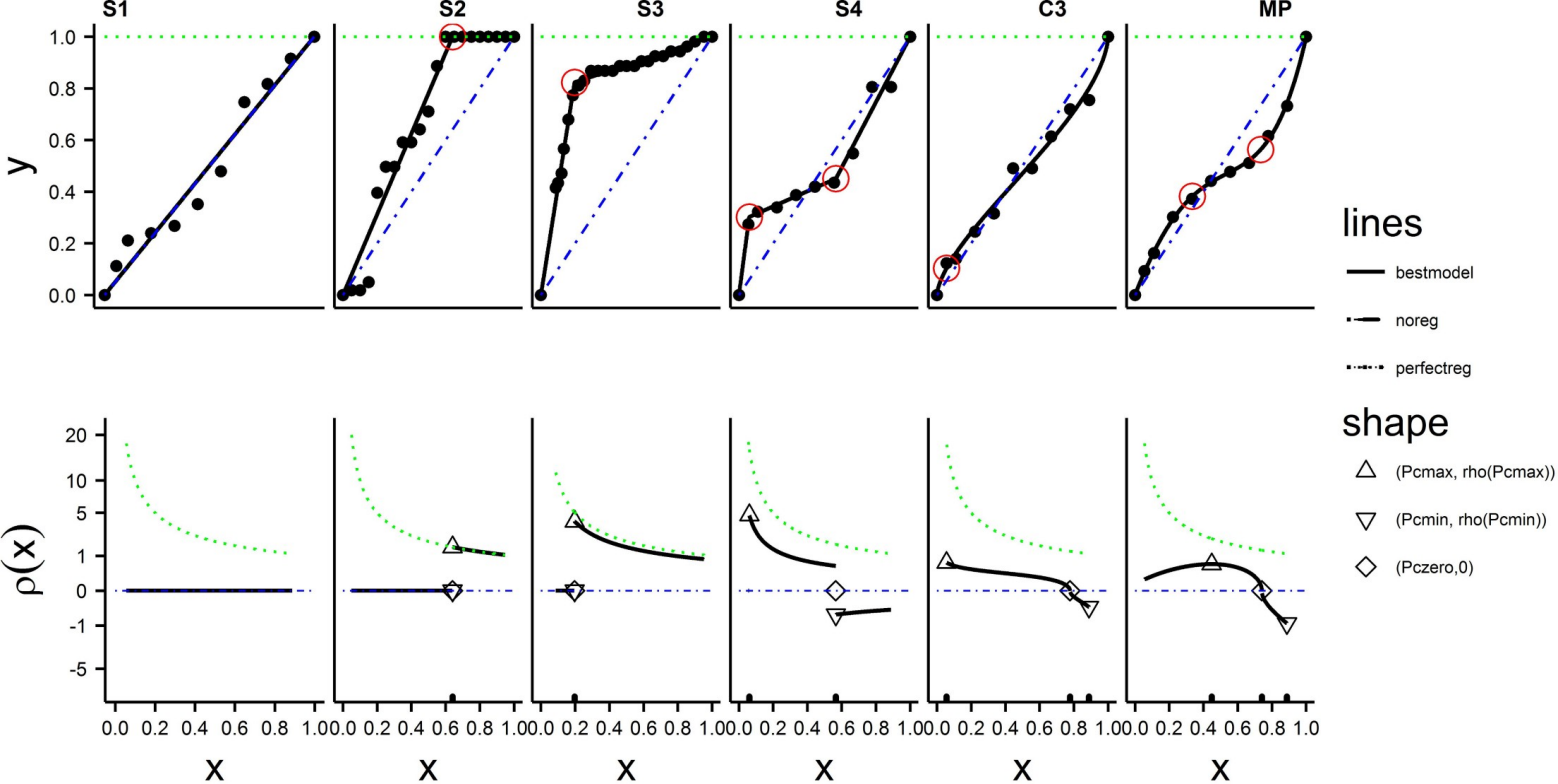
Hypoxia plots (top panels) and matched regulation profiles (bottom panels) for a selected animals with best fitting models S1,S2,S3,S4,C3, and MP. Solid black lines (──) are the predicted lines for y and ρ(x). Observed data points are marked with black dots. The alternating dot-dash blue line (— · — · — ·) is the expected line for a non-regulator for both y and ρ(x). The dotted green line (····) is the expected line for a perfect regulator for both y and ρ(x). In the hypoxia plot threshold points are marked by a red circle. In the regulation profile points with x values at P_c-min_, P_c-zero_ and P_c-max_, are marked with ▽, ◊ and Δ, respectively, and their locations on the x-axis are marked with tics just above the axis.

From the 68 animals studied, six were chosen which had best fitting models S1, S2, S3, S4, C3, and MP, respectively, and are depicted in Figs 3 and 4. Model C2 is not included because it was not the best model for any animals examined. The fits for each of these six animals to each of the seven models are shown in Fig 3. Fig 4 gives the summary of the analysis, using the best model, for each of the same six animals in Fig 3. The upper panels of Fig 4 contain the observed data points, the curve defined by the best *f(x)*, and threshold values, if present. The lower panels of Fig 4 contain the regulation profile, *ρ(x)*, determined from the best *f(x)* using Eq (1), with locations of P_c-min_, P_c-max_, and P_zero_, if present, indicated on the plot. The six hypoxia curves in Fig 4 show how well the best model fit the observed data. Values of the descriptive statistics for the curves given in Fig 4 and conclusions about regulation for each animal are given in Table E in S1 Appendix. Plots similar to the individual panels of Fig 4, for each of the 68 animals are given in S1 Results.

### Analysis of all animals

**Fig 5:**
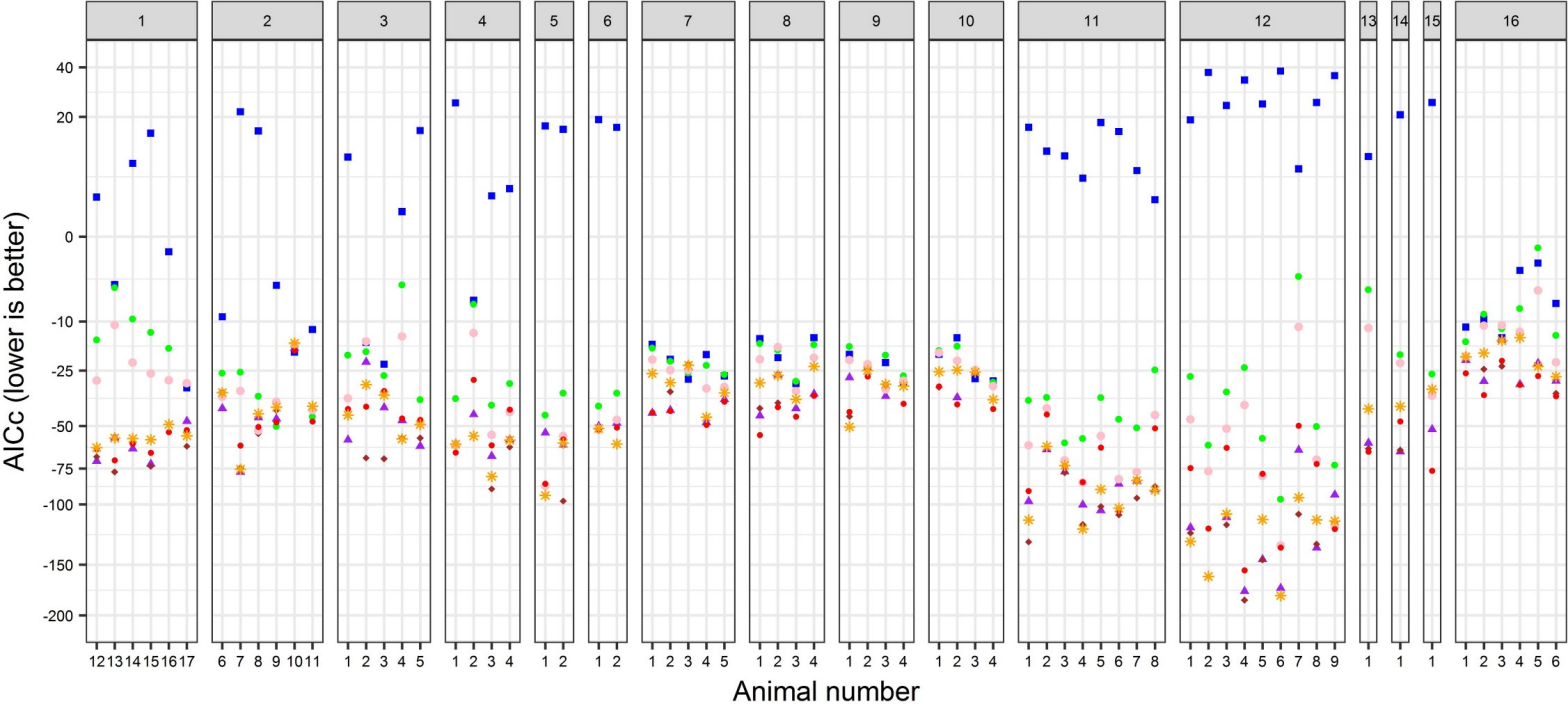
AICc values for the fit of each model to each of the 68 animals. Species groups are given in different panels with the label at the top. The legend for shapes and colors is the following: blue square = S1, green dot = S2, purple triangle = S3, purple diamond = S4, ● = C2, red dot = C3, orange asterisk = MP.

The hypoxia curves for nearly all (67/68) of the animals give very good fit to the best model. The average R^2^ (coefficient of determination) is 0.984 and for all but one animal the R^2^ is above 0.95. Fig 5 gives AICc values for the fit of every model to every animal. Model S1 (blue squares) is the poorest fitting model for almost all animals, indicating most animals are regulators of some type. S1 (classic conformer) is the best fitting model for only three animals which are the only non-regulators among all animals studied. Model S2 (small green dots) fits better than model S1 for most animals but almost always gives a worse fit than models S3, S4, C2, C3 or MP, indicating most of the animals that are regulators are not classic regulators, instead, these regulators are referred to as partial regulators [28,29,30] when describing an intermediate level of regulation. Model S2 is the best fitting model for only one animal. For the remaining 64 animals, the number with best fit by models S3, S4, C2, C3 or MP are 10, 23, 0, 21, and 10 animals, respectively.

**Fig 6:**
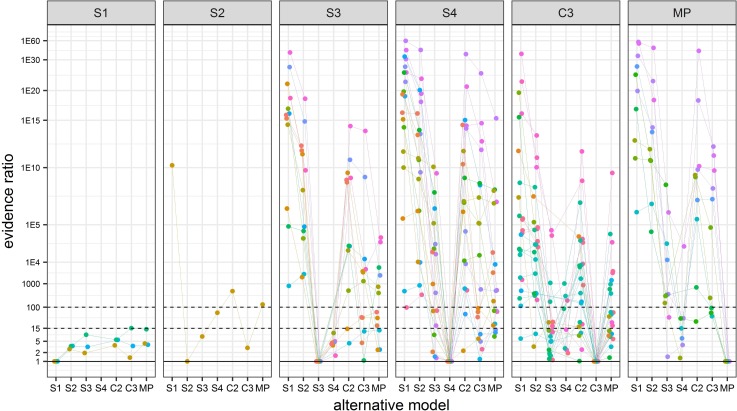
Plot of evidence ratio (ER) vs alternatives to the best model for each of the 68 animals. The ER value for the best model is 1 and values greater than 1 are the fold increase in likelihood in favor of the best model over the alternative model. Each panel gives the plot for all animals with the best model given at the top of the panel. Values from a single animal have the same color and are connected with lines.

The first panel in Fig 6, labeled S1, contains data for the three animals for which model S1 was the best model and gives ER values comparing each of the other models to S1. This panel shows that alternative models S2, S3, S4, C2, C3, and MP all gave ER values less than 15. Thus, model S1 is not the ‘uniquely best model’ for any of the 68 animals studied, but is the ‘best model’ for three of them. This result is expected because model S1 is a special case of each of the other models.

The second panel in Fig 6 labeled S2 shows the one animal for which model S2 was the best. Models S3 and C3 are ‘equivalent alternative models’ and the ER value against model S1 is near 10^10^. The third panel in Fig 6 labeled S3 contains data for the 15 animals for which model S3 was the best model. It gives ER values comparing each of the other models to model S3 and shows that when S3 is the best model, models S1 and S2 are never an equivalent alternative model. Panel S3 also indicates for animals with model S3 as the best model it is often the uniquely best model and when it is not, one of models S4, C2, C3, or MP is an equivalent alternative model. Panels S4, C3, and MP of Fig 6 have similar interpretations. Panels S4 and MP show that models S1 or S2 are never equivalent alternatives to models S4 or MP. Panel C3 indicates that among the 23 animals with C3 as the best model one had S1 as an equivalent alternative model and one had S2 as an equivalent alternative model. Model C2 is not included in Fig 6 because it is not the best fitting model for any of the 68 animals.

**Fig 7:**
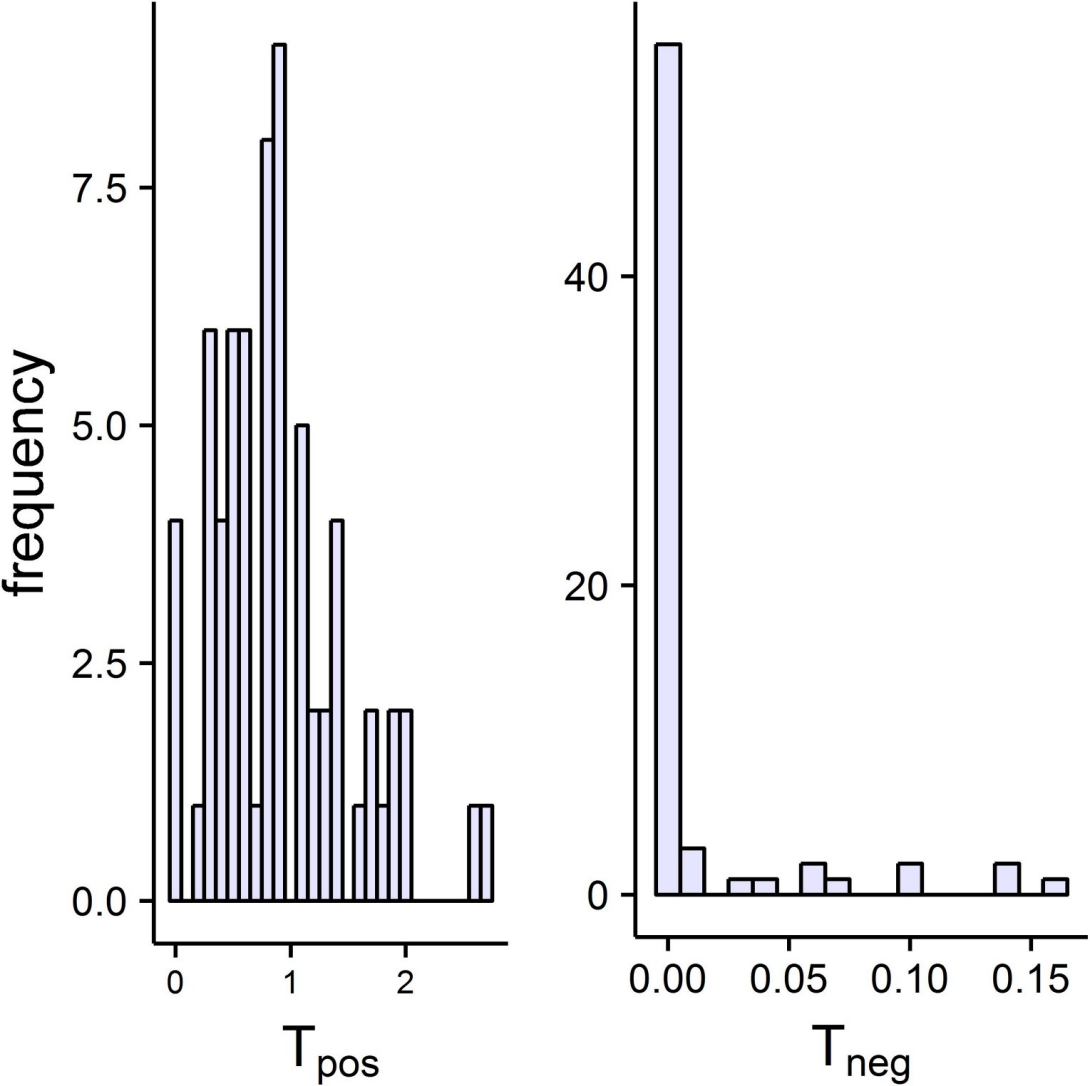
Histograms of T_pos_ and T_neg_ for all 68 animals.

**Fig 8:**
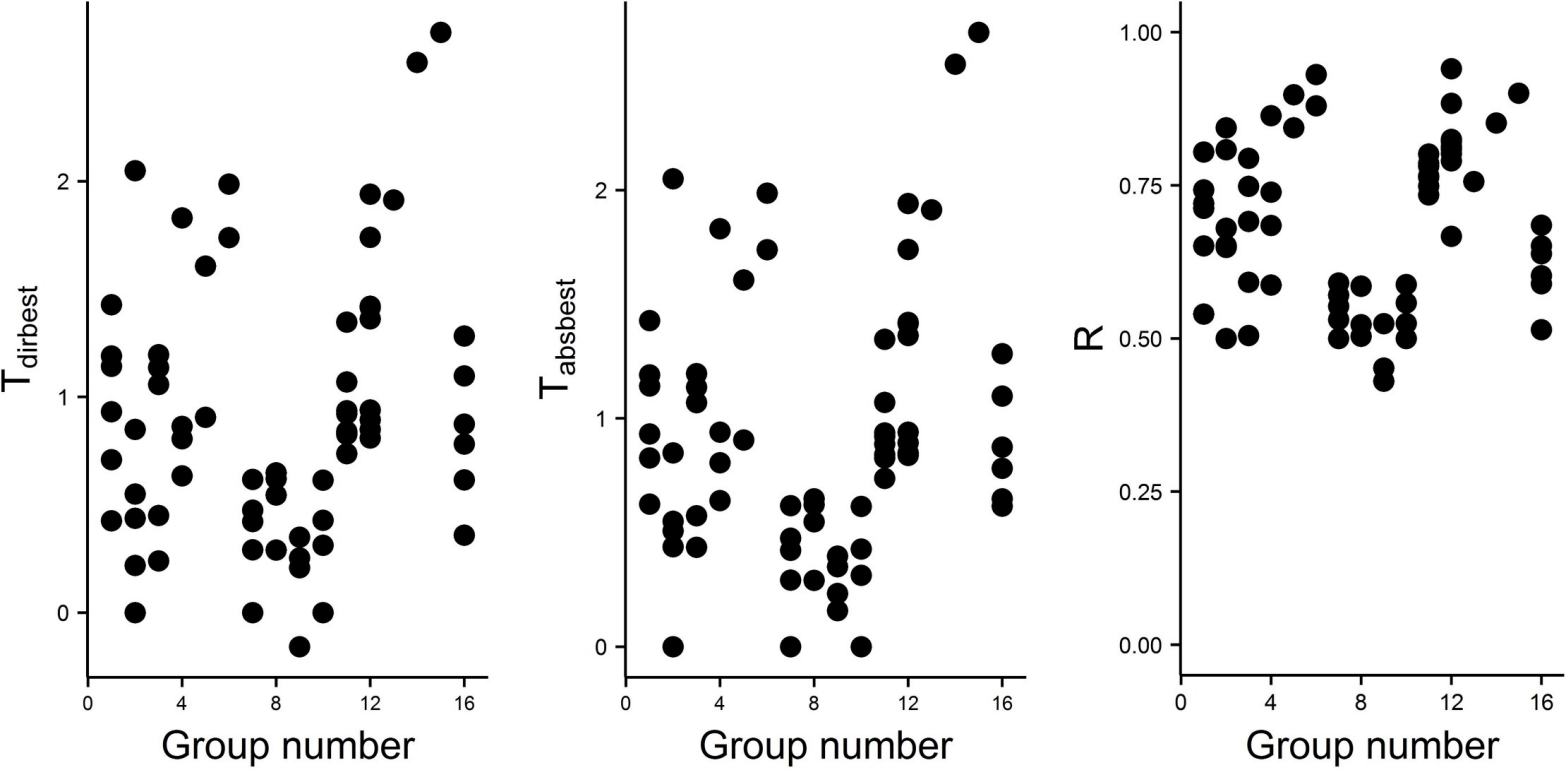
Plots of T_pos_, T_neg_, and R vs group number for all 68 animals.

Fig 7 gives histograms of T_pos_ and T_neg_ from the best model for each of the 68 animals. The left panel shows the great majority of the 68 animals performed positive regulation that ranged from low values near 0 to high values near 2.7, and only four animals had essentially zero positive regulation. The right panel shows many animals (55/68) had zero negative regulation and the T_neg_ of the rest ranged from near 0 to near 0.16. Fig 8 gives T_pos_, T_neg_, and R for the best model for each animal plotted versus group number.

**Fig 9:**
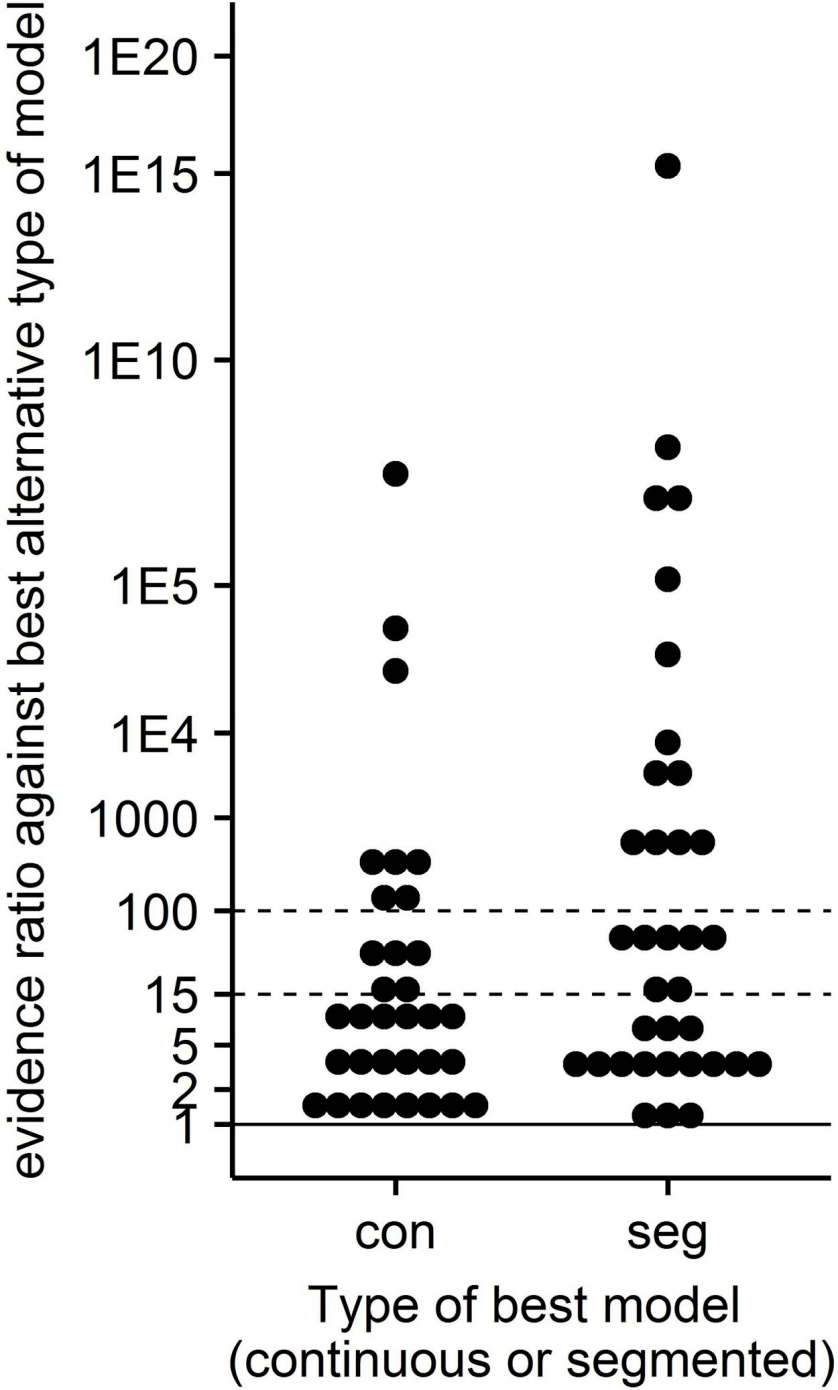
Scatter plot of evidence ratios (ER) vs type (continuous, segmented) of best model for each of the 68 animals. When the best model of of type con the ER is the ratio of the probability of the best segmented model to the probability of the best continuous model. When the best model of of type seg the ER is the ratio of the probability of the best continuous model to the probability of the best segmented model. An ER value<15 is taken here to indicate an approximately equivalent alternative model.

Comparison of the best segmented model and the best continuous model is made by computing ΔAICc = AICc(best segmented)—AICc(best continuous) for each animal separately and then computing the ER with the best model as the reference. For 17 animals, the best model was continuous and none of the segmented models were ‘acceptable alternative models’. For 22 animals, the best segmented model was segmented and none of continuous models were ‘acceptable alternative models’. The dot plots of the ER values are shown in Fig 9 separately for animals with continuous or segmented best models. In Fig 9, a dot for the continuous group indicating ER≥15 indicates an animal with a continuous best model that has 15 or more times the likelihood than the best segmented model. Similarly, a dot at or above 15 in the segmented group indicates an animal with segmented best model that has 15 or more times the likelihood than the best continuous model.

## Discussion

### The traditional models S1 and S2 (classic conformer and classic regulator, respectively) are not adequate for most animals

An overall conclusion from Fig 6 is that for 64 of the 68 animals there is a pattern of regulation that cannot be explained by the conventional models S1 or S2 which model classic conformity and classic regulation, respectively. The evidence for this claim is that when one of the newly proposed models (S3, S4, C2, C3, MP) is the best fitting model, the conventional models S1 and S2 almost always (62/64) gave ER > 100, indicating much poorer fit to the data (see Fig 6). The only two exceptions to this are animals that had C3 as the best model. One had both S1 and S2 as “equivalent alternative models” and the other had S2 as an “equivalent alternative” model. These exceptions can happen because for certain parameter values, model C3 can be very similar to models S1 or S2 (see panel C3 of Fig 4 and panels 2 and 11 of Fig 2). The primary deficiency of the conventional model S2 is that it assumes y is constant with a value of 1 when x > P_t_ and few animals give this pattern. For many animals, as x drops from 1, y also immediately drops from 1 (See Fig 4, Panel S3, S4, C3, MP) as well as many figures in S1 Results. Models (S3, S4, C2, C3 and MP) describe this immediate drop in y as well as describing a more precipitous drop in y at lower x values. When the drop in y is below the line y = x the pattern is labeled negative regulation. This pattern may be described by models S3, S4, C3 and MP but not by models S1 or S2. This finding indicates the traditional parameter P_c_ alone (or equivalently, P_t_ of model S2) does not adequately describe the shape of the hypoxia curve or the regulation profile for many animals.

### Regulation profiles suggest some animals perform regulation at very low *Po*_*2*_ levels

When S4 or a continuous model was the best fitting model, there was often positive regulation occurring at x values very close to zero (see panels S4 and C3 and MP of Fig 4 and results in S1 Results. The second to the lowest x value is the lowest level at which this study reports regulation because the lowest x value is zero and ρ(x*)* was computed only for values between and including the second lowest and second highest x values in the data. Of the 68 animals 32 had their maximum positive regulation at the lowest *Po*_*2*_ level for which regulation can be detected in this study. This observation suggests that for a substantial proportion of animals, there may be positive regulation occurring at oxygen levels below what this study is able to detect. Thus, for these animals, the data cannot be taken as evidence for a threshold value of x below which all regulation stops. Of course, the proportion of animals capable of regulation at low *Po*_*2*_ levels will depend on which groups are included in the study. Certainly some groups cannot regulate at such low *Po*_*2*_ levels.

### Very few animals can be described as ‘classic’ conformers

Only three of 68 animals had S1 as the best model. Also, when a model other than S1 was the best model for an animal the ER values indicated S1 was an “acceptable alternative model” only for one animal. The analysis here strongly indicates there are at most only four true non regulators among the 68 animals studied here. Most of the animals showing low levels of positive or negative regulation in this study would have been classified as conformers in previous studies using the traditional P_c_ method, however, the ER comparisons to model S1 showed they had more than zero regulation (see Fig 4 panels C3 and MP and many profiles given in S1 Results). It might be possible that very low T_pos_ and T_neg_ values result from processes other than active regulation but that cannot be determined from analysis of hypoxia curves.

### Positive regulation is very common and quite variable in amount

Sixty four of the 68 animals showed positive regulation. The histogram of T_pos_ values seen in Fig 7 shows a range from 0 to 2.69 with an average of 0.8858 and the inter-quartile range is from 0.497 to 1.138. This high amount of variability along with the uni-modality of the histogram of T_pos_ (see Fig 7) supports the idea that there is a continuum of regulation types rather than a clustering around just two types called “strong regulators” and “conformers”. The highest value observed for T_pos_ is 2.69 which is equivalent to the amount of total positive regulation found in a strong classic regulator with a P_c_ value of 0.068. There seem to be differences among the species groups in amount of total positive regulation, T_pos_, as seen in Fig 8, where groups 7, 8, 9, and 10 have means less than those of groups 4, 5, 6, 11, 12, 13, 14 and 15. Because the 68 animals studied here are not a random sample of all animals, we cannot present these results as an accurate measure of the amount of diversity of regulation types for all animals. We do propose that the methods presented here for studying diversity of regulation types are much better than studies using only the traditional models, S1 and S2, to estimate a P_c_ value and will likely reveal differences undetected by previous methods.

The third panel of Fig 8 shows the regulation index (R) gives a result somewhat similar to T_pos_. This similarity occurs because there are only very low levels of negative regulation in the animals studied here. If there were more extensive negative regulation then T_pos_ and R would be less similar and could show different patterns. If an investigator is only interested in the total amount of positive regulation and if there is little or no negative regulation in the population under study, R may give a pattern of differences similar to that found with T_pos_.

### Negative regulation is common and when it is present it is at low levels

Negative regulation indicated by T_neg_>0 was found in 16 of 68 animals and it was always at a low level. The histogram of T_neg_ values seen in Fig 7 shows a range from 0 to 0.159 with an average of 0.013 and the lower and upper limits of the inter-quartile range are zero and zero, respectively, indicating high skewness to the right. When negative regulation is present it is often found in an inverse sigmoidal-shaped hypoxia curve. In these cases, the first critical point (P_c1_,V_c1_) is a shift down type and is above y = x and a second critical point (P_c2_,V_c2_) is a shift up type and is below y = x. Panels S4, C3 and MP of Fig 4 are examples of such curves in which an animal performs positive regulation in some parts of the curve and negative regulation in other parts of the curve. There is no obvious pattern of differences in amount of negative regulation among the groups studied here, however, this study should not taken as evidence of lack of species variation for negative regulation as the statistical power associated with such a claim from the data presented here is surely small. A study with a larger sample of organisms showing negative regulation might reveal differences among species.

### Many animals do not fit a segmented linear model

Segmented models assume regulation occurs in just two or three modes. For example, models S2 and S3 assume regulation is either ‘on’ or ‘off’ and model S4 assume there are three modes which might be called ‘no regulation’, ‘some regulation’, and ‘more regulation’. In contrast, continuous models assume there is a continuum of regulation intensities due to infinitesimal changes in regulation with infinitesimal changes in x. Among the 68 animals, 32 were best fit by one of the continuous models C2,C3, or MP suggesting a continuous nature of regulation is occurring in at least some animals. Further, a pair-wise comparison of the best continuous model to the best segmented model using ER over all 68 animals is shown in Fig 9. Seventeen animals had a continuous model as the best model and among these, nine animals had ER≥15 and six had ER≥100 indicating that a continuous model is dramatically better than a segmented one for these animals (see Fig 9). Twenty-two animals had a segmented best model and for seventeen it was the uniquely best model (ER≥15) and for 11 of these the ER against the best continuous model was above 100. Twenty-nine animals indicated the best continuous and best segmented models are approximately equivalent models. These results suggest that for some animals there is a continuum of regulation instead of two modes of regulation as has been asserted by some researchers in the past [7,19,20,13,21].

In conclusion, we have shown that for most animals, the proposed new approach for extracting information from *Po*_*2*_ -*Vo*_*2*_ curves gives a better description than the traditional methods currently used. The first step of the method is similar to the method of Marshal [9] and others in that it uses a variety of linear or non-linear functions as required to fit the observed pattern in a curve. The second step of our method uses a new approach to describe patterns present in the curve that are putatively due to regulation of *Vo*_*2*_. A key feature in the new method is the concept of instantaneous regulation which proposes the amount of regulation varies continuously with *Po*_*2*_. The function *ρ(x)* is called the regulation profile and is proposed to describe the intensity of regulation at x (the standardized *Po*_*2*_) and is a complete description of the pattern of regulation. The function *ρ(x)* may be equal to zero or have positive or negative values at different values of x. When *ρ(x)* >0, the drop in *Vo*_*2*_ with decreasing *Po*_*2*_ is less than would occur in the absence of regulation. This is the traditional type of regulation which we call positive regulation. When ρ(x) < 0, the drop in *Vo*_*2*_ with decreasing *Po*_*2*_ is more than would occur in the absence of regulation. We call this type of regulation “negative regulation” which is the opposite of positive regulation. When *ρ(x)* = 0, there is no regulation at x. Metrics describing the pattern of regulation are extracted from *ρ(x)* and are denoted T_pos_, T_neg_, P_c-min_, P_c-max_, and P_zero_. When the relationship in the *Po*_*2*_ -*Vo*_*2*_ curve is represented by a segmented linear function with two segments (also called the broken stick model) P_c-min_, P_c-max_, and P_zero_ are all equal to each other and have the same value as P_c_ of the traditional model. When the *Po*_*2*_ -*Vo*_*2*_ curve is more complex than the broken stick model, the P_c_ parameter is not sufficient to describe the pattern on regulation and the new parameters, P_c-min_, P_c-max_, and P_zero_, may each have a unique value and comprise a better description of the pattern of regulation and may be used instead of P_c_. In this sense our method is a generalization of the traditional method. The metrics T_pos_, T _neg_ quantify the total amount of positive and negative regulation indicated by a *Po*_*2*_ -*Vo*_*2*_ curve, respectively. We analyzed data from 68 animals and found that most animals show patterns that are better described by the new method than by the traditional model that uses only P_c_ as a descriptor of the type and amount of regulation. Because the new method is sensitive to response patterns that are not measurable with the traditional method, its use may be more successful in finding differences among species in their response to hypoxic environments.

## Supporting information

S1 Appendix (DOCX)

S1 Results (DOCX)

S1 Data (DOC)

## Abbreviations

Pc: the oxygen concentration.

x: standardized ambient oxygen concentration

y: standardized oxygen consumption rate concentration

f(x): a function describing the dependence of y on x

ρ(x): a function giving amount of instantaneous regulation at x

κ(x): curvature of a function evaluated at x

Kcutoff: a value for determining threshold points of a function, f(x)

Tpos (L1, L2): amount of positive regulation in interval (L1, L2)

Tneg (L1, L2): amount of negative regulation in interval (L1, L2)

S1,S2,S3,S4,C2,C3,MP: names of selections for f(x)

R: the regulation value

R2: coefficient of determination

AIC: Akaike Information Criterion

AICc: Akaike Information Criterion adjusted for small sample size

ΔAICc: difference between AICc value for two models

ER: Evidence Ratio (best model used as the reference)

Pt, Vt: x and y coordinates of a threshold point for a selected f(x)

Pc-min, Pc-max, and Pc-zero: minimum, maximum, and zero’s of the function ρ(x)
