# Supplementary material for: Assessment of oxygen consumption in response to progressive hypoxia
Source: PLoS One. 2018 Dec 21;13(12):e0208836. doi: 10.1371/journal.pone.0208836 (PMC6303046; doi:10.1371/journal.pone.0208836)
Supplement: S1 Appendix — (DOCX) [file pone.0208836.s001.docx]

Appendix

Table A Formulae for *ρ(x)* for each of the seven models

| model | *ρ(x)* = f(x)/x -*f’(x)* |
| --- | --- |
| S1 | *ρ(x)* ≡ 0 |
| S2 | $\rho\left( x \right)=I_{0\leq x<P_{t}}0+I_{P_{t}\leq x<1}x^{-1}$ |
| S3 | $\rho\left( x \right)=I_{0\leq x<P_{t}}0+I_{P_{t}\leq x<1}{\left\{ V_{t}-P_{t}\left( \frac{1-V_{t}}{1-P_{t}} \right) \right\}x}^{-1}$ |
| S4 | $\rho\left( x \right)=I_{0\leq x<P_{t,1}}0+I_{P_{t,1}\leq x<P_{t,2}}\left( x \right){\left\{ V_{t,1}-P_{t,1}\left( \frac{V_{t,2}-V_{t,1}}{P_{t,2}-P_{t,1}} \right) \right\}x}^{-1}+I_{P_{t,2}\leq x\leq1}\left( x \right){\left\{ V_{t,2}-P_{t,2}\left( \frac{1-V_{t,2}}{1-P_{t,2}} \right) \right\}x}^{-1}$ |
| C2 | $\rho\left( x \right)=\left( \frac{1}{x} \right)\left[ 1-\left( 1-x \right)^{\frac{1}{P_{p}}}\left\{ 1-\left( \frac{1}{P_{p}} \right)x\left( 1-x \right)^{-1} \right\} \right]$ |
| C3 | $\rho\left( x \right)=\left( \frac{1}{x} \right)\left[ 1-\left( 1-x^{\frac{1}{V_{p}}} \right)^{\frac{1}{P_{p}}}\left\{ 1-\left( \frac{1}{V_{p}} \right)\left( \frac{1}{P_{p}V_{p}} \right)x^{\frac{1}{V_{p}}}\left( 1-x^{\frac{1}{V_{p}}} \right)^{-1} \right\} \right]$ |
| MP | $\rho\left( x \right)=\sum_{i=0}^{k} b_{i}\left( 1-i \right)x^{i-1}$ |

Table B Formulae for *τ*_dir_ for each of the seven models

| model | $\tau_{dir}\left( L_{1},L_{2} \right)=\int_{L_{1}}^{L_{2}} \rho\left( x \right)dx \mathrm{where}0< L1< L2 \leq1$ |
| --- | --- |
| S1 | *τ*_dir_(L_1_,L_2_) ≡ 0 |
| S2 | $\tau_{dir}\left( L_{1},L_{2} \right)=\left( \frac{1}{L_{2}-L_{1}} \right)\left[ ln\left\{ max\left( L_{2},P_{t} \right) \right\}-ln\left\{ max\left( L_{1},P_{t} \right) \right\} \right]$ |
| S3 | $\tau_{dir}\left( L_{1},L_{2} \right)=\left( \frac{1}{L_{2}-L_{1}} \right)\left[ ln\left\{ max\left( L_{2},P_{t} \right) \right\}-ln\left\{ max\left( L_{1},P_{t} \right) \right\} \right]$ |
| S4 | $\tau_{dir}\left( L_{1},L_{2} \right)=\left( \frac{1}{L_{2}-L_{1}} \right)\left\{ V_{t,1}-P_{t,1}\left( \frac{V_{t,2}-V_{t,1}}{P_{t,2}-P_{t,1}} \right) \right\}\left\{ ln\left( P_{t,2} \right)-ln\left( P_{t,1} \right) \right\}+\left\{ V_{t,2}-P_{t,2}\left( \frac{1-V_{t,2}}{1-P_{t,2}} \right) \right\}\left\{ ln\left( L_{2} \right)-ln\left( P_{t,2} \right) \right\}$ if L_1_< P_t,1_<P_t,2_ < L_2_ |
| C2 | $\tau_{dir}\left( L_{1},L_{2} \right)=\left( \frac{1}{L_{2}-L_{1}} \right)\int_{L_{1}}^{L_{2}} \left( \frac{1}{x} \right)\left[ 1-\left( 1-x \right)^{\frac{1}{P_{p}}}\left\{ 1-\left( \frac{1}{P_{p}} \right)x\left( 1-x \right)^{-1} \right\} \right]dx$ |
| C3 | $\tau_{dir}\left( L_{1},L_{2} \right)=\left( \frac{1}{L_{2}-L_{1}} \right)\int_{L_{1}}^{L_{2}} \left( \frac{1}{x} \right)\left[ 1-\left( 1-x^{\frac{1}{V_{p}}} \right)^{\frac{1}{P_{p}}}\left\{ 1-\left( \frac{1}{V_{p}} \right)\left( \frac{1}{P_{p}V_{p}} \right){x^{\frac{1}{V_{p}}}\left( 1-x^{\frac{1}{V_{p}}} \right)}^{-1} \right\} \right]dx$ |
| MP | $\tau_{dir}\left( L_{1},L_{2} \right)=\left( \frac{1}{L_{2}-L_{1}} \right)\left[ b_{0}\left\{ ln\left( L_{2} \right)-ln\left( L_{1} \right) \right\}+\sum_{2}^{k} b_{i}\left( \frac{1-i}{i} \right)\left( L_{2}^{i}-L_{1}^{i} \right) \right]$ |

Table C Formulae for *τ*_abs_ for each of the seven models

|  | $\tau_{abs}\left( L_{1},L_{2} \right)=\int_{L_{1}}^{L_{2}} \left\vert\rho\left( x \right) \right\vert dx$ where 0< L_1_< L_2_ ≤ 1 |
| --- | --- |
| S1 | *τ*_abs_(L_1_,L_2_) ≡ 0 |
| S2 | $\tau_{abs}\left( L_{1},L_{2} \right)=\left( \frac{1}{L_{2}-L_{1}} \right)\left[ ln\left\{ max\left( L_{2},P_{t} \right) \right\}-ln\left\{ max\left( L_{1},P_{t} \right) \right\} \right]$ |
| S3 | $\tau_{abs}\left( L_{1},L_{2} \right)=\left( \frac{1}{L_{2}-L_{1}} \right)\left\vert\frac{V_{t}-P_{t}}{1-P_{t}} \right\vert\left[ ln\left\{ max\left( L_{2},P_{t} \right) \right\}-ln\left\{ max\left( L_{1},P_{t} \right) \right\} \right]$ |
| S4 | $\tau_{abs}\left( L_{1},L_{2} \right)=\left( \frac{1}{L_{2}-L_{1}} \right)\left\{ \left\vert V_{t,1}-P_{t,1}\left( \frac{V_{t,2}-V_{t,1}}{P_{t,2}-P_{t,1}} \right) \right\vert\right\}\left\{ ln\left( P_{t,2} \right)-ln\left( P_{t,1} \right) \right\}+\left\{ \left\vert V_{t,2}-P_{t,2}\left( \frac{1-V_{t,2}}{1-P_{t,2}} \right) \right\vert\right\}\left\{ ln\left( L_{2} \right)-ln\left( P_{t,2} \right) \right\}$ if L_1_< P_t,1_<P_t,2_ < L_2_ |
| C2 | $\tau_{dir}\left( L_{1},L_{2} \right)=\left( \frac{1}{L_{2}-L_{1}} \right)\int_{L_{1}}^{L_{2}} \left( \frac{1}{x} \right)\left[ \left\vert1-\left( 1-x \right)^{\frac{1}{P_{p}}}\left\{ 1-\left( \frac{1}{P_{p}} \right)x\left( 1-x \right)^{-1} \right\} \right\vert\right]dx$ |
| C3 | $\tau_{dir}\left( L_{1},L_{2} \right)=\left( \frac{1}{L_{2}-L_{1}} \right)\int_{L_{1}}^{L_{2}} \left( \frac{1}{x} \right)\left[ \left\vert1-\left( 1-x^{\frac{1}{V_{p}}} \right)^{\frac{1}{P_{p}}}\left\{ 1-\left( \frac{1}{V_{p}} \right)\left( \frac{1}{P_{p}V_{p}} \right){x^{\frac{1}{V_{p}}}\left( 1-x^{\frac{1}{V_{p}}} \right)}^{-1} \right\} \right\vert\right]dx$ |
| MP | $\tau_{dir}\left( L_{1},L_{2} \right)=\left( \frac{1}{L_{2}-L_{1}} \right)\int_{L_{1}}^{L_{2}} \left\vert\sum_{0}^{k} b_{i}\left( i-1 \right)x^{i-1} \right\vert dx$ |

Table D Information on species used in the analysis

| groupno | Species name | n* | Source |
| --- | --- | --- | --- |
| 1 | *Dreissena polymorpha* ( 5 °C acclimated) | 6 | [1] |
| 2 | *Dreissena polymorpha* (15 °C acclimated) | 6 | [1] |
| 3 | *Dreissena polymorpha* (25 °C acclimated) | 5 | [1] |
| 4 | *Upogebia pugettensis* | 4 | [2] |
| 5 | *Palaemon serratus* | 2 | [3] |
| 6 | *Palaemon elegans* | 2 |  |
| 7 | *Neanthes virens* | 5 | [4] |
| 8 | *Nereis diversicolor* | 4 |  |
| 9 | *Aphrodita aculeata* | 4 |  |
| 10 | *Arenicola marina* | 4 |  |
| 11 | *Hemiscyllium ocellatum* | 8 | [5] |
| 12 | *Ammodytes hexapterus* | 9 | [6] |
| 13 | *Pleurobrachia bachei* | 1 | [7] |
| 14 | *Mnemiopsis leidyi* | 1 |  |
| 15 | *Bolinopsis infundibulum* | 1 |  |
| 16 | *Ciona intestinalis* | 6 | [8] |

n* = number of individuals analyzed for the species group

Table E Description and statistics for graphs presented in Figure 4.

| groupno | animalid | model | description | T_pos_ | T_neg_ | P_t_,  V_t_ § | P_t2_,  Vt_2_ | P_c-min_ | P_c-max_ | P_c-zero_ |
| --- | --- | --- | --- | --- | --- | --- | --- | --- | --- | --- |
| 7 | 3 | S1 | The animal shows no regulation of any type. The animal is a conformer. | 0 | 0 | ‡,  ‡ | ‡,  ‡ | ‡ | ‡ | ‡ |
| 2 | 9 | S2 | The animal is a moderate positive regulator and performs no negative regulation. The P_c-max_ = P_c-zero_ value of 0.640 indicates max regulation at 0.64 and a change in regulation mode at this value. | 0.438 | 0 | 0.640  ‡ | ‡,  ‡ | 0.640 | 0.640 | 0.64 |
| 12 | 8 | S3 | The animal is a strong positive regulator and performs no negative regulation. The P_c-max_ = P_c-zero_ value of 0.197 indicates max regulation at 0.197 and a change in regulation mode at this value. | 1.421 | 0 | 0.197,  0.824 | ‡,  ‡ | 0.197 | 0.197 | 0.197 |
| 16 | 3 | S4 | The animal is a moderate positive regulator when 0.171<x>0.935 and performs weak negative regulation when x>0.935. The P_c-max_ of 0.171 indicates regulation at low oxygen levels. It has high T_pos_ because it performs positive regulation at low oxygen levels. | 0.504 | 0.144 | 0.061,  0.302 | 0.566,  0.451 | 0.566 | 0.061 | 0.566 |
| 9 | 4 | C3 | The animal is a weak positive regulator when 0.056<x<0.888 and a very weak negative regulator when x>0.888.The P_c-max_ value indicates max positive regulation at very low oxygen level of 0.056. | 0.221 | 0.012 | 0.056,  0.103 | ‡,  ‡ | 0.888 | 0.056 | 0.777 |
| 9 | 1 | MP | The animal is a moderate positive regulator when x<0.777 and performs weak negative regulation when x>0.777. Its maximum positive regulation is at 0.448. | 0.325 | 0.072 | 0.335,  0.382 | 0.735,  0.563 | 0.888 | 0.448 | 0.741 |

§ P_t_ or P_t1_ and V_t_ or V_t1 ,_ ‡ indicates values are undefined

**References**

1. Alexander, J. E. Jr and R. F. McMahon. 2004. Respiratory response to temperature and hypoxia in the zebra mussel *Dreissena polymorpha*. Comparative Biochemistry and Physiology A 137: 425-434.
2. Thompson, R. K. and A. W. Pritchard. 1969. Respiratory adaptations of two burrowing crustaceans, *Callianassa californiensis* and *Upogebia pugettensis*. The Biological Bulletin 136: 274-287.
3. Taylor, A. C. and J. I. Spicer. 1989. Interspecific comparison of the respiratory response to declining oxygen tension and the oxygen transporting properties of the blood of some palaemonid prawns (Crustacea: Palaemonidae). Marine Behaviour and Physiology 14: 81-91.
4. Shumway, S. E. 1979. The effects of body size, oxygen tension and mode of life on the oxygen uptake rates of polychaetes. Comparative Biochemistry and Physiology A 64: 273-278.
5. Routley, M. H., G. E. Nilsson and G. M. C. Renshaw. 2002. Exposure to hypoxia primes the respiratory and metabolic responses of the epaulette shark to progressive hypoxia. Comparative Biochemistry and Physiology A 131: 313-321.
6. Quinn, T. and D. E. Schneider. 1991. Respiration of the teleost fish *Ammodytes hexapterus* in relation to its burrowing behavior. Comparative Biochemistry and Physiology A 98: 71-75.
7. Thuesen, E. V., L. D. Rutherford Jr. and P. L. Brommer. 2005. The role of aerobic metabolism and intragel oxygen in hypoxia tolerance of threee ctenophores: *Pleurobrachia bachei*, *Bolinopsis infundibulum* and *Mnemiopsis leidyi*. Journal of the Marine Biological Association of the United Kingdom 85: 627-633.
8. Shumway, S. E. 1978. Respiration, pumping activity and heart rate in *Ciona intestinalis* exposed to fluctuating salinities. Marine Biology 48: 235-242.
