# Supplementary figures and images for: Assessment of oxygen consumption in response to progressive hypoxia
Source: PLoS One. 2018 Dec 21;13(12):e0208836. doi: 10.1371/journal.pone.0208836 (PMC6303046; doi:10.1371/journal.pone.0208836)

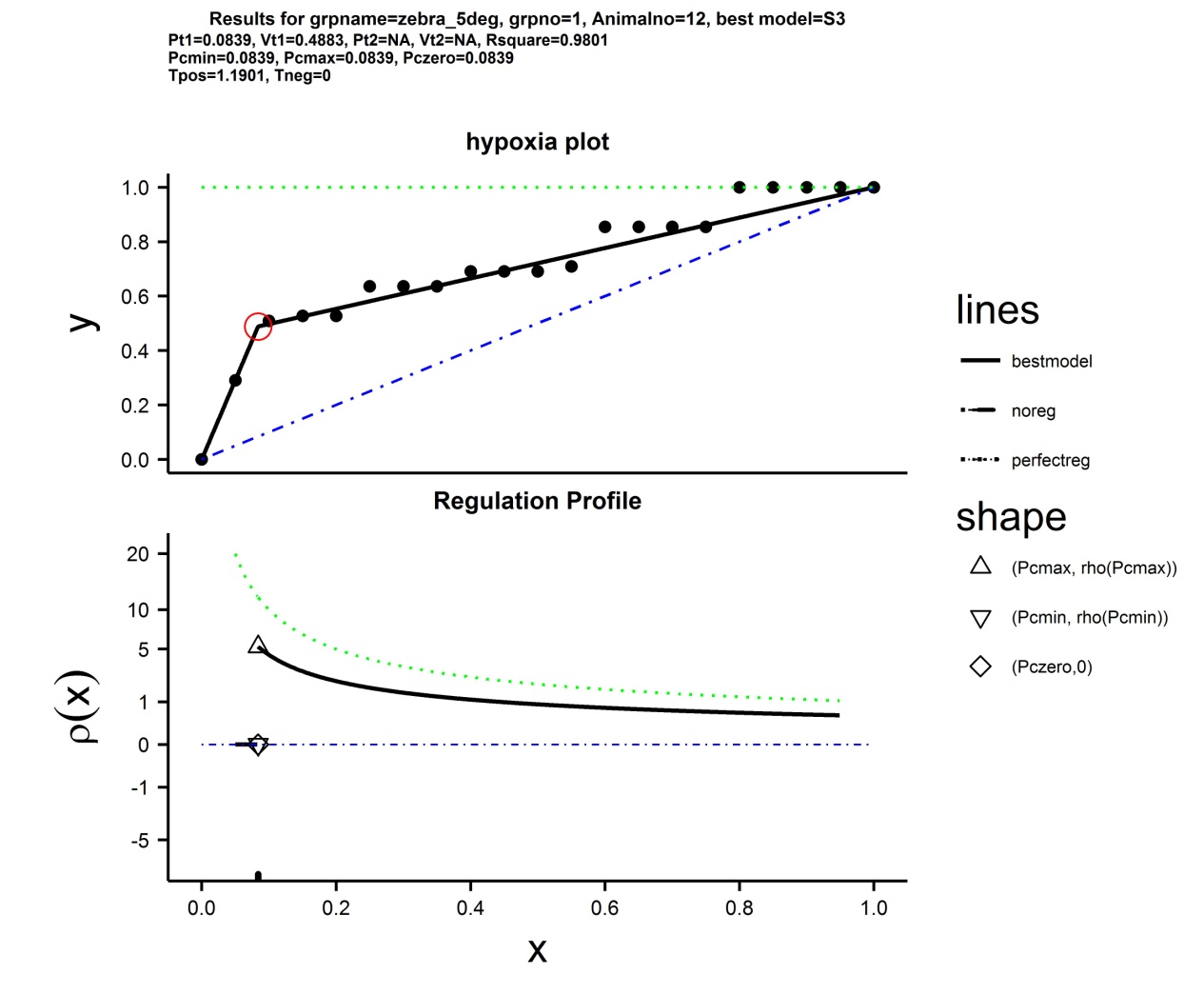


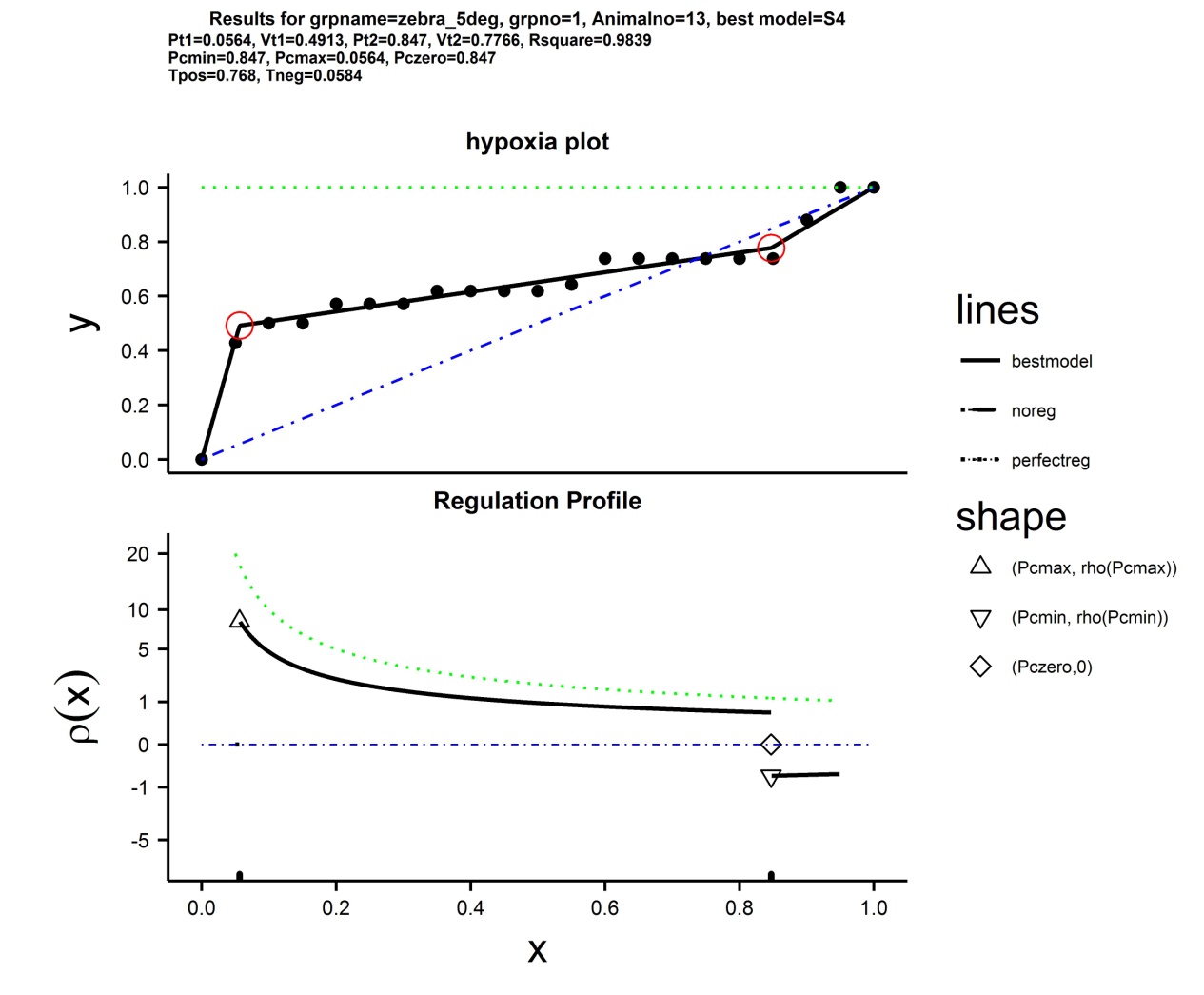

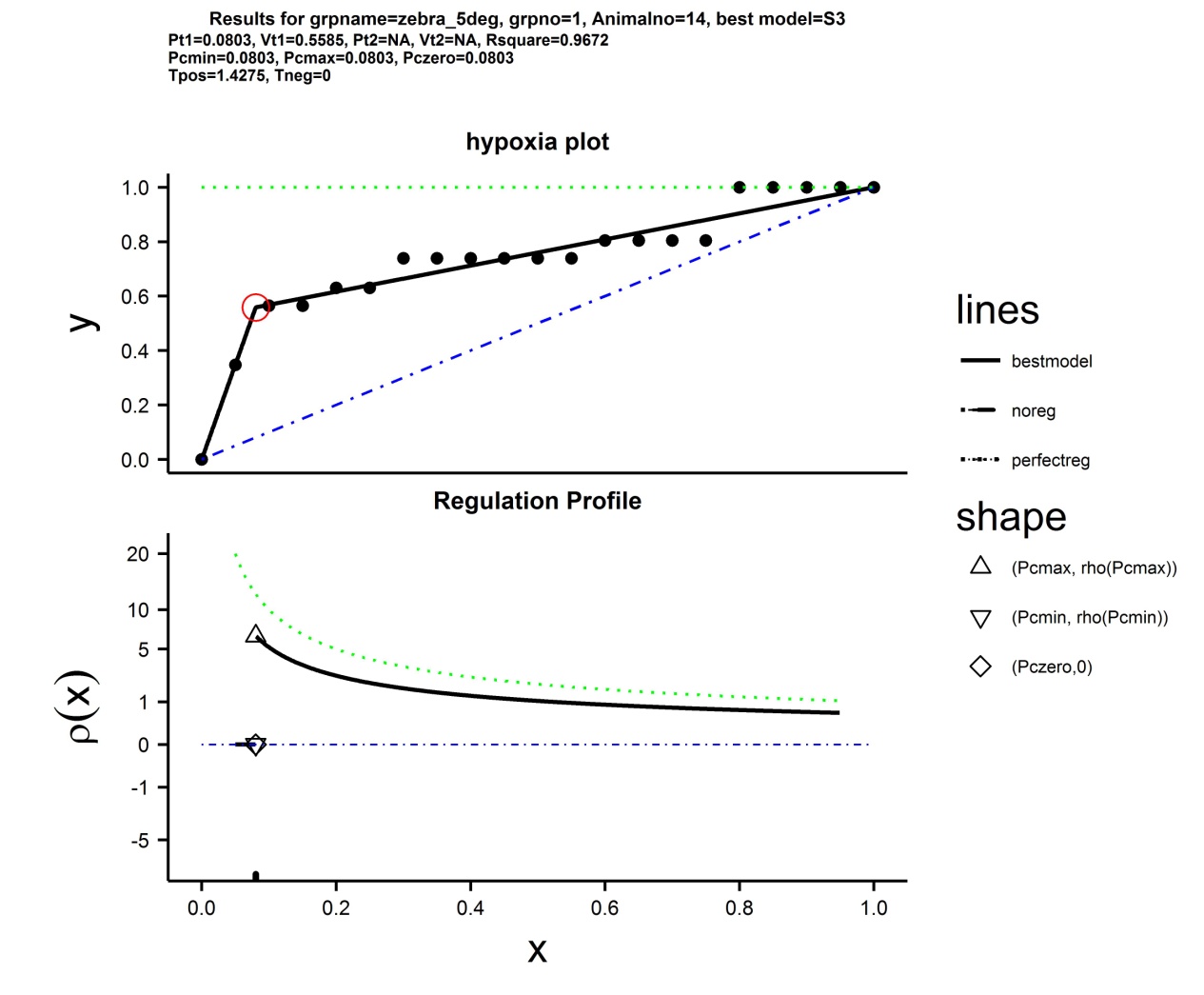

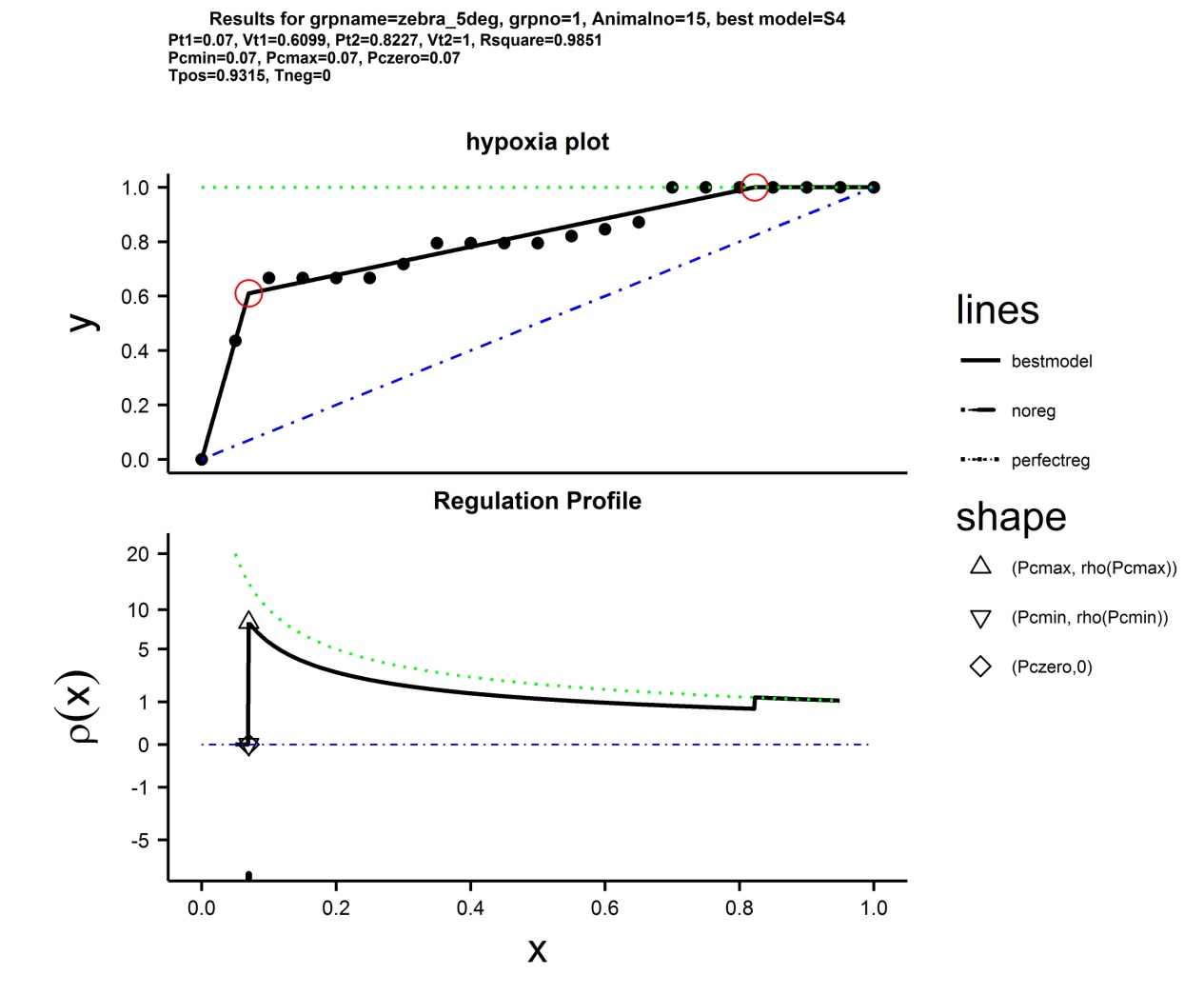

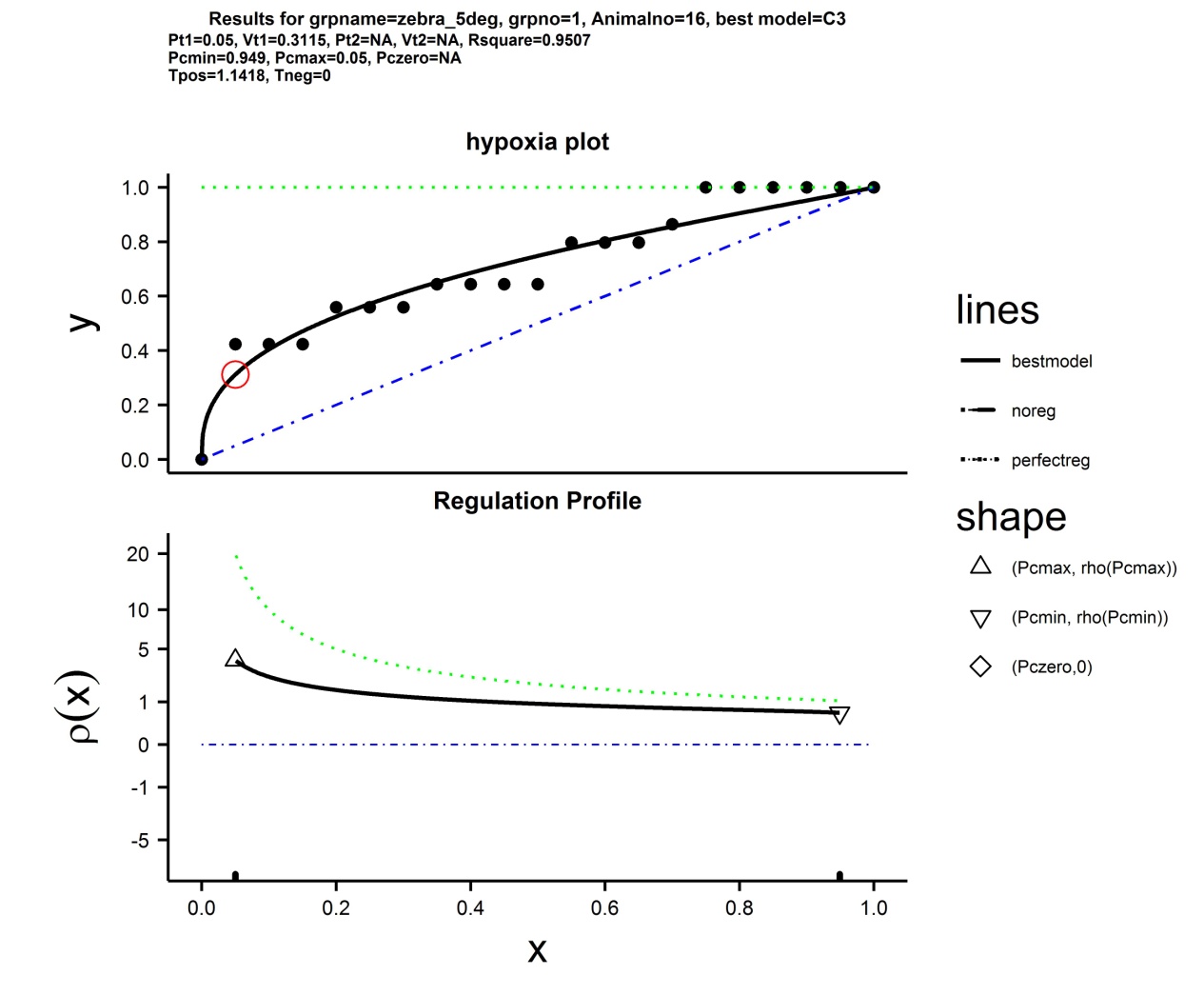

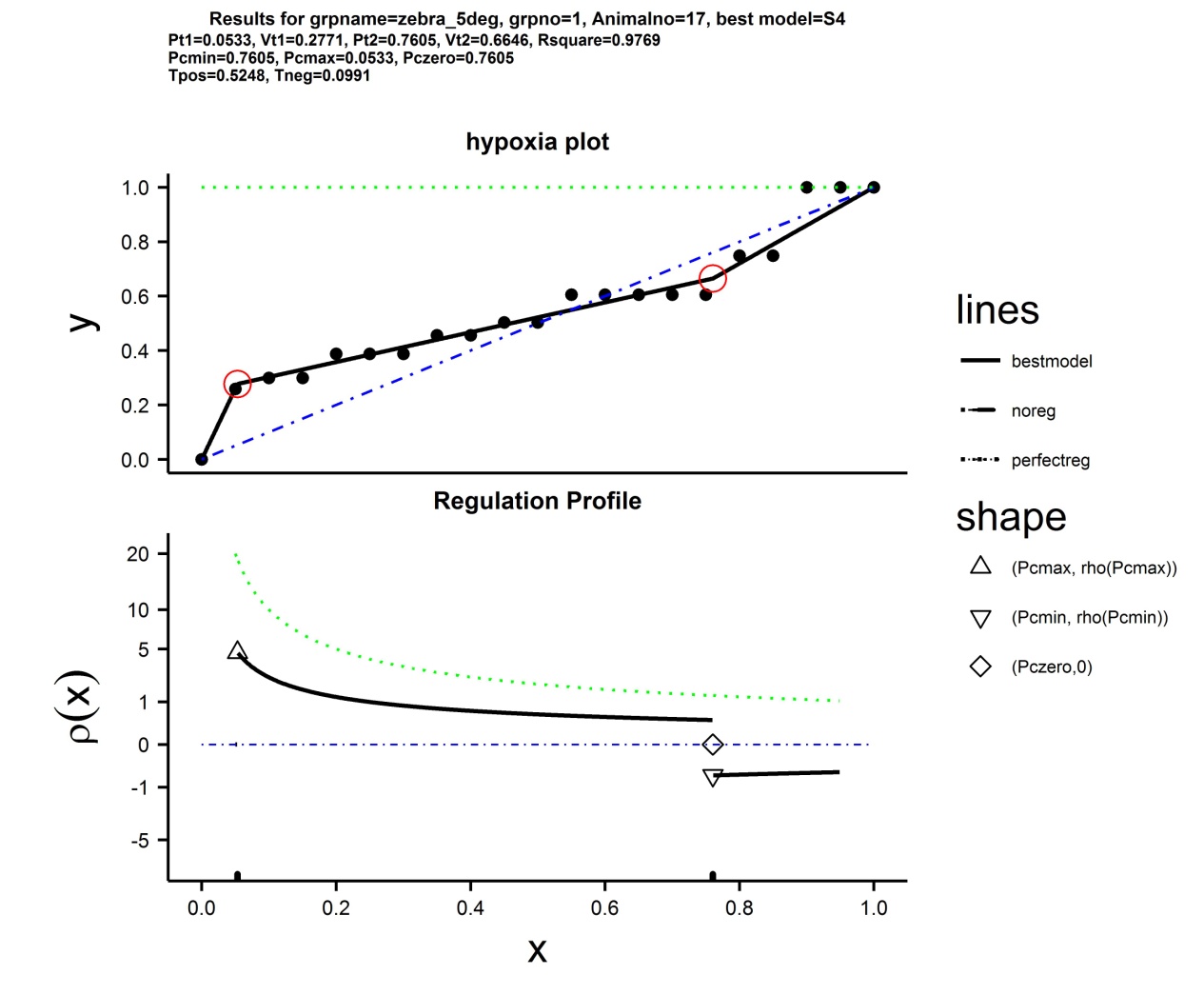

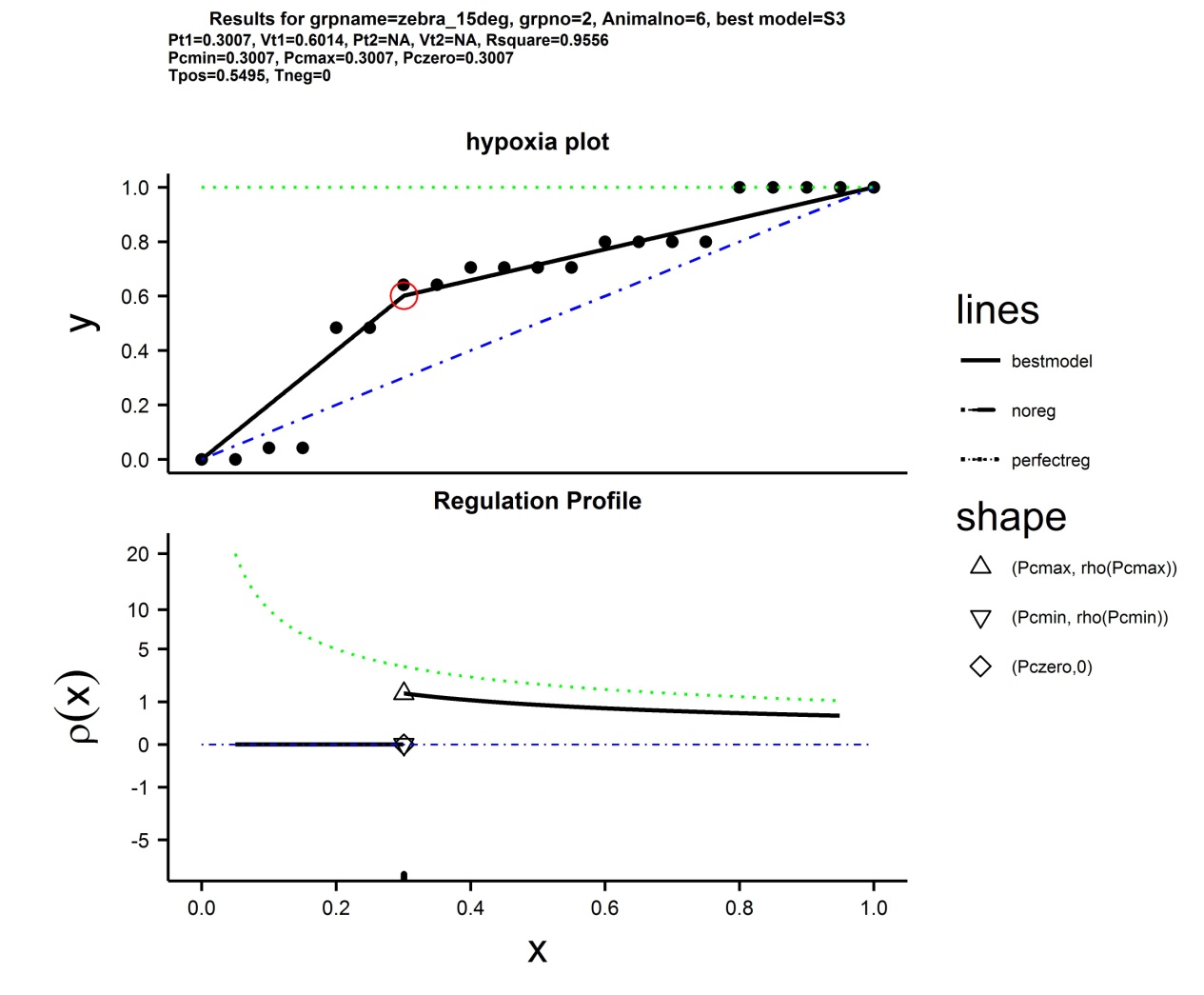

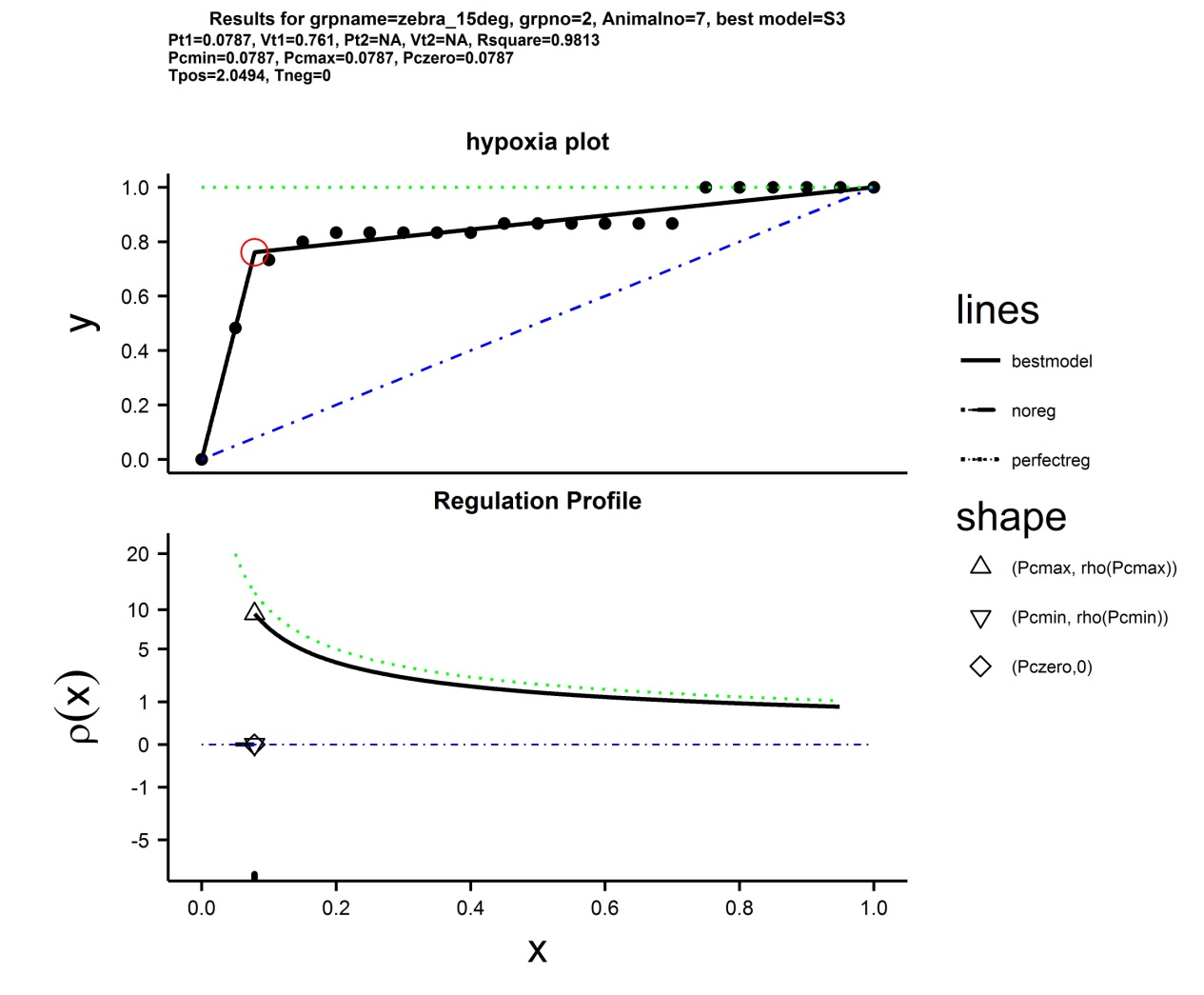

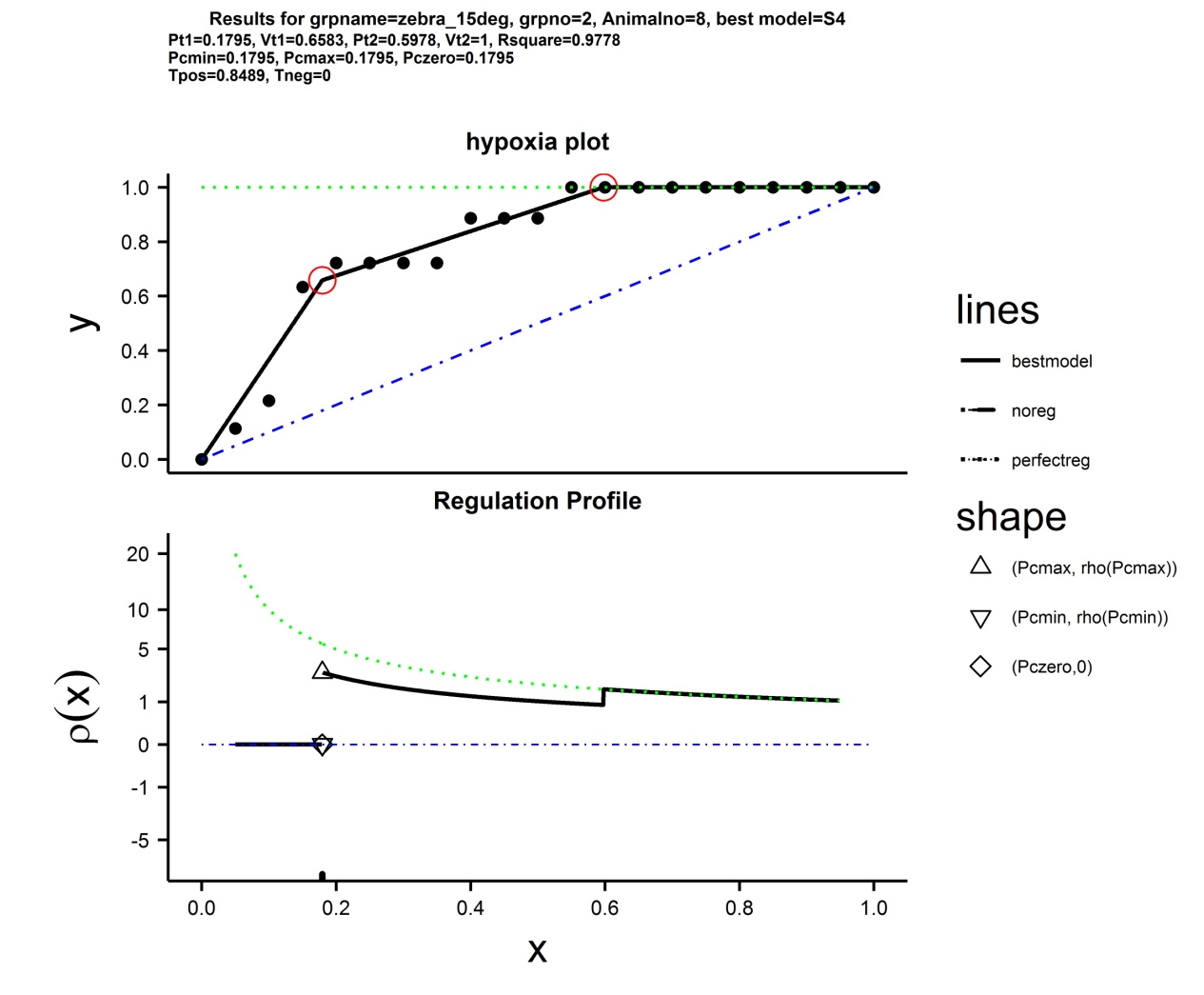

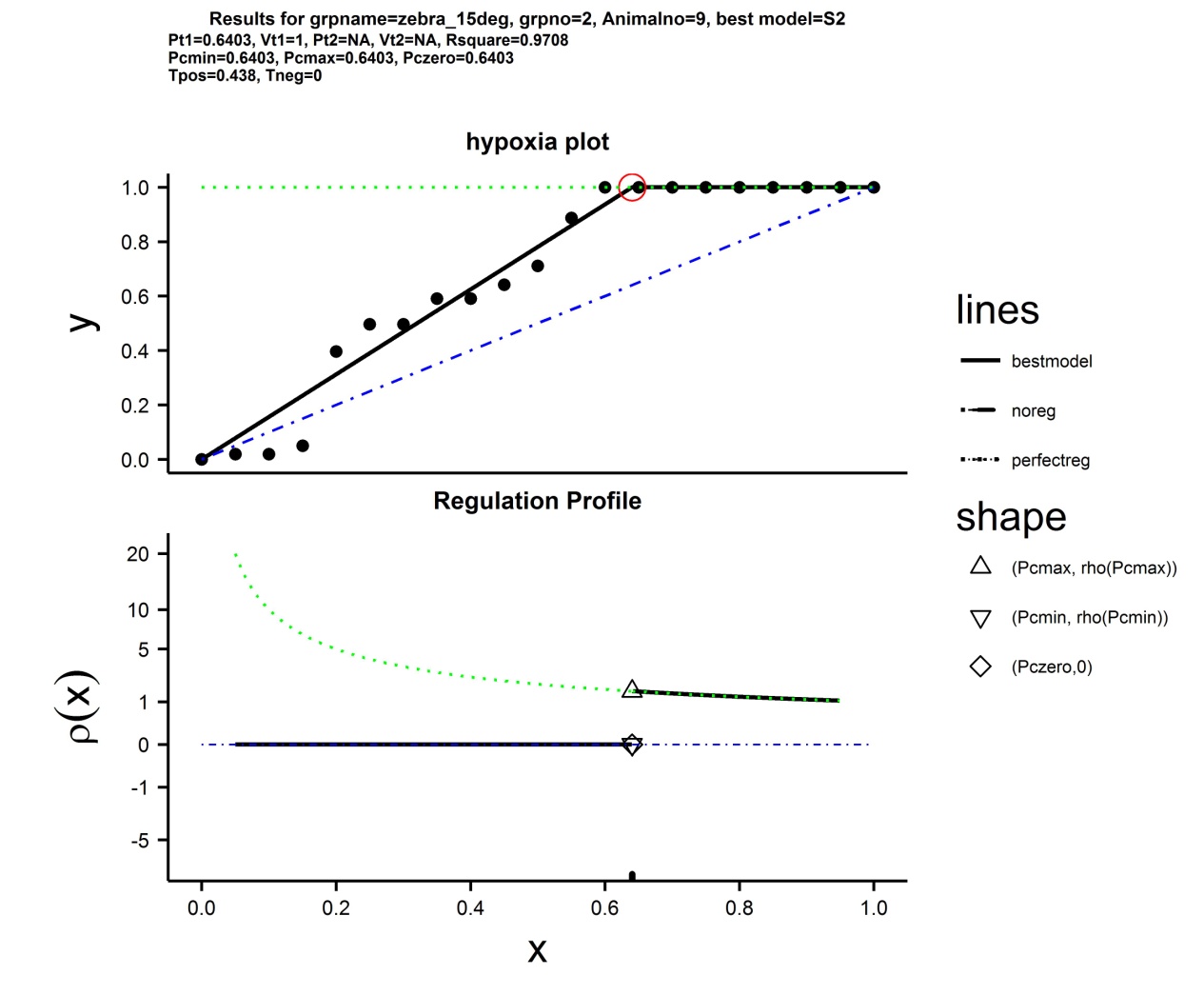

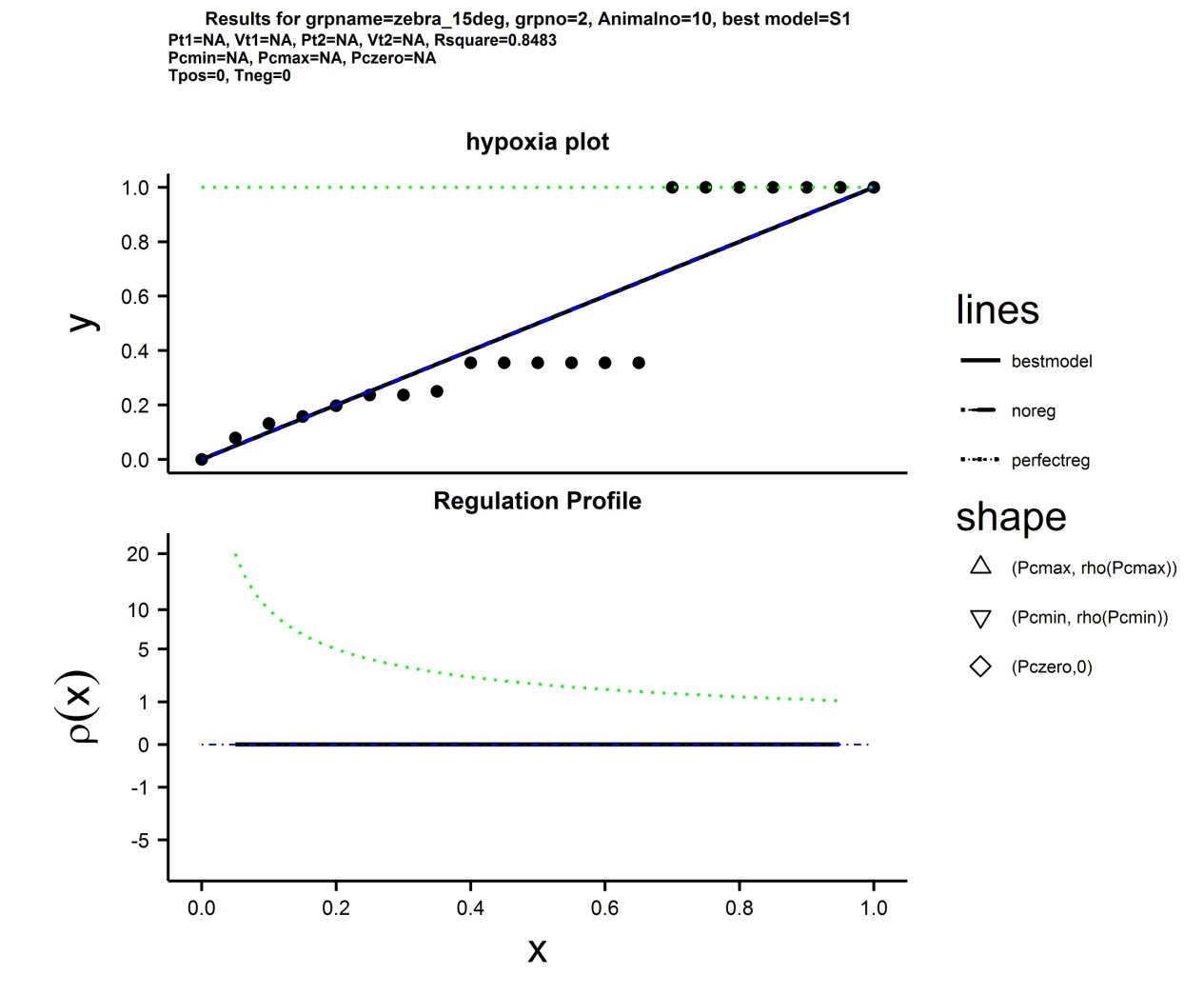

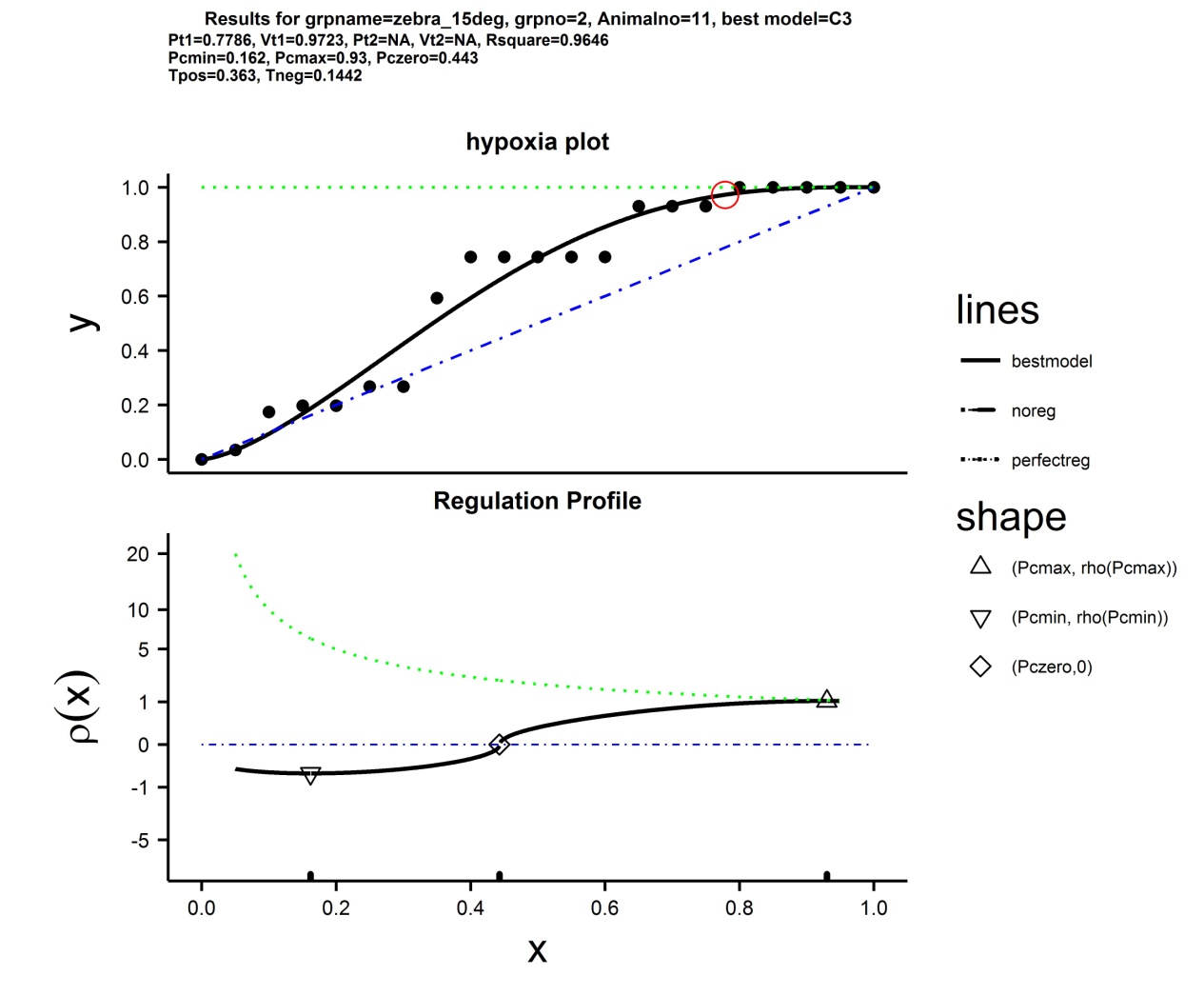

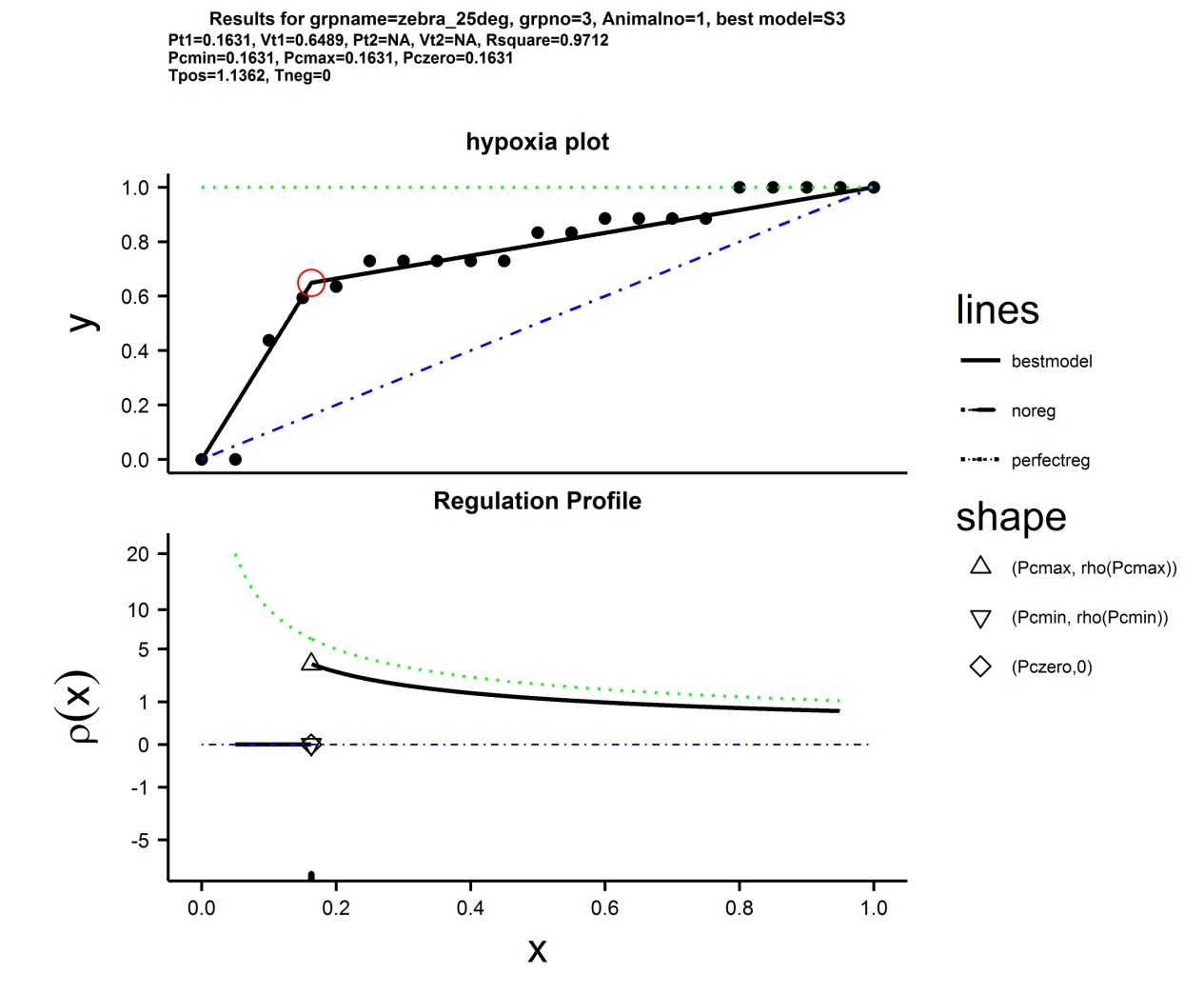

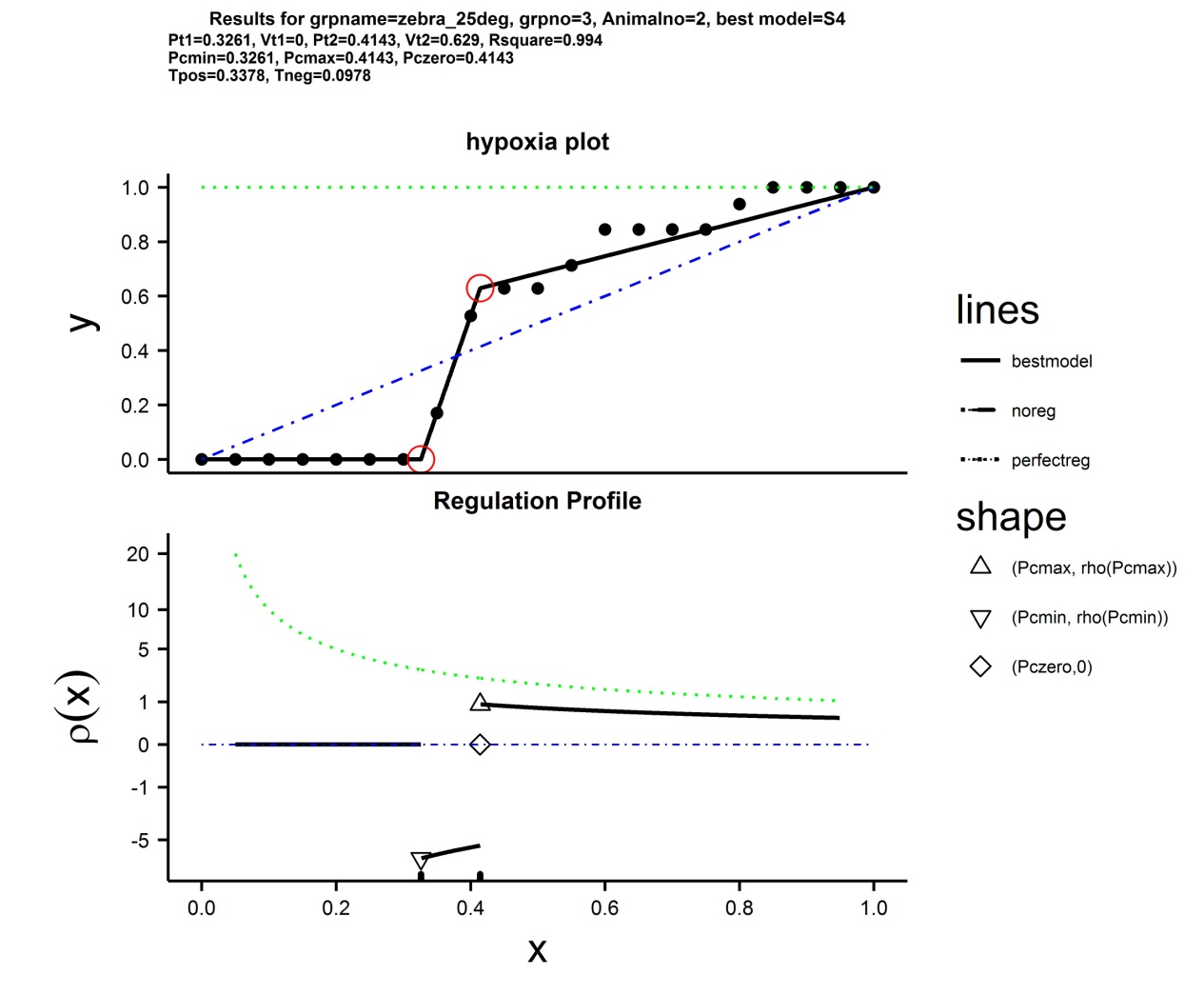

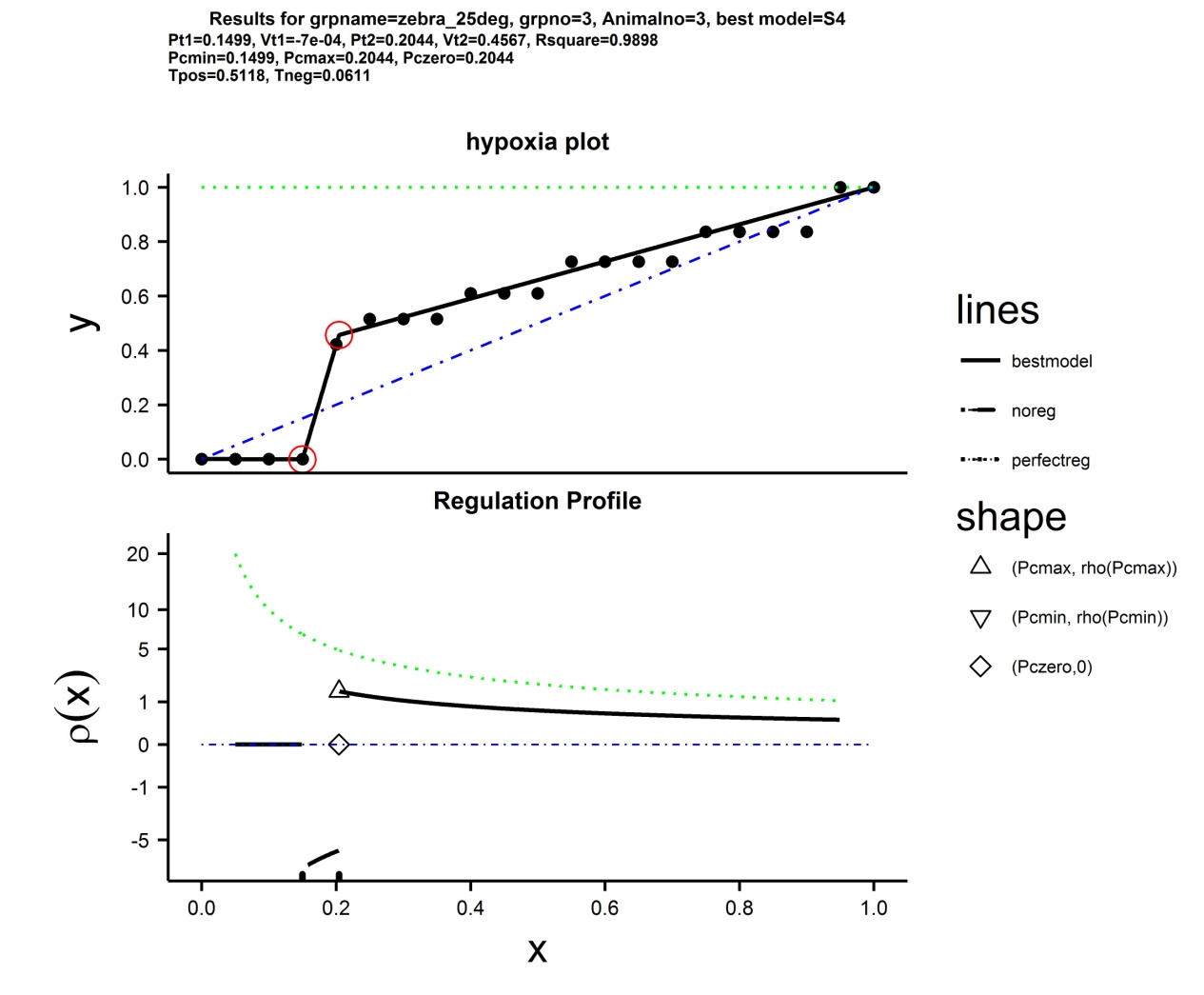

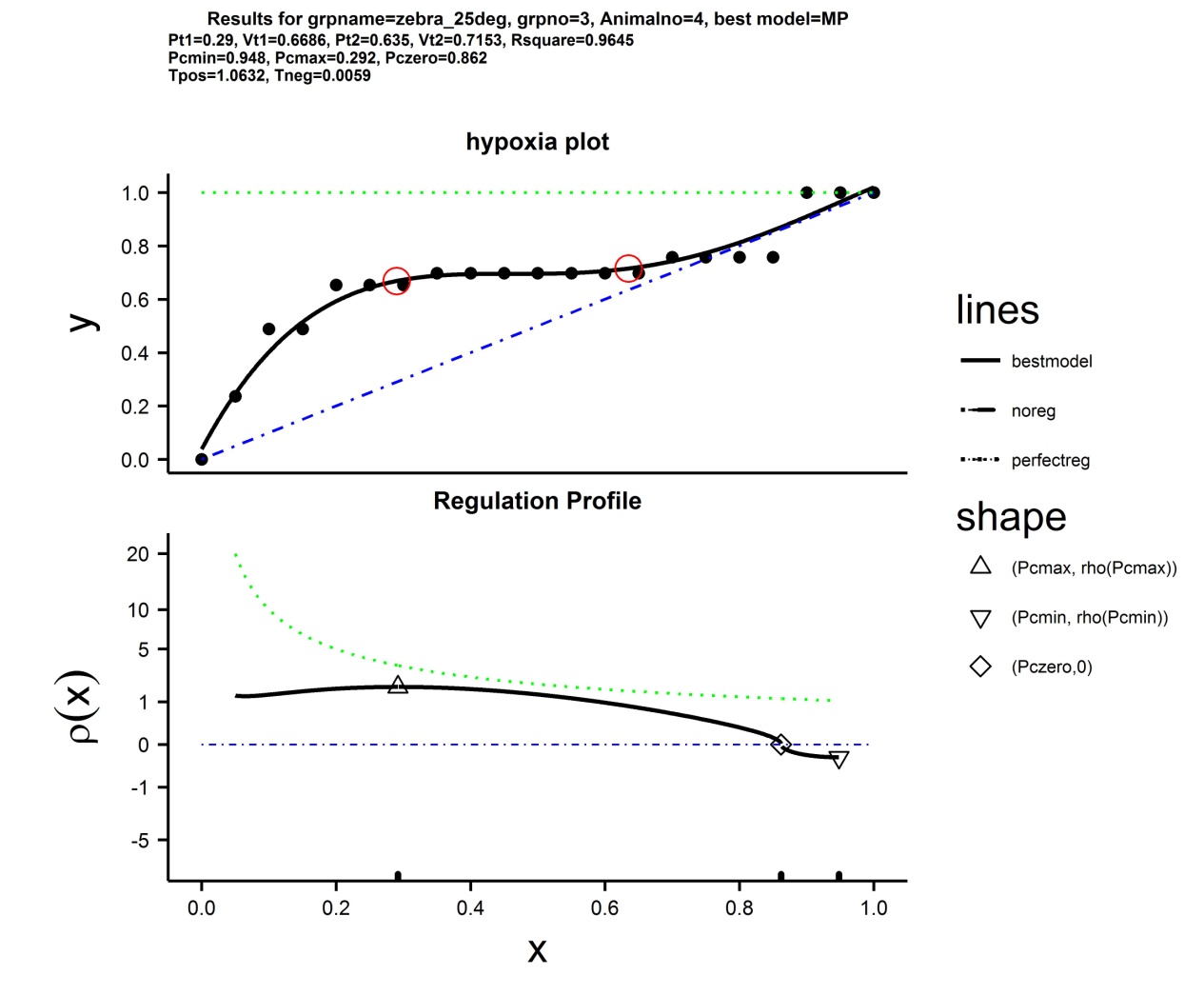

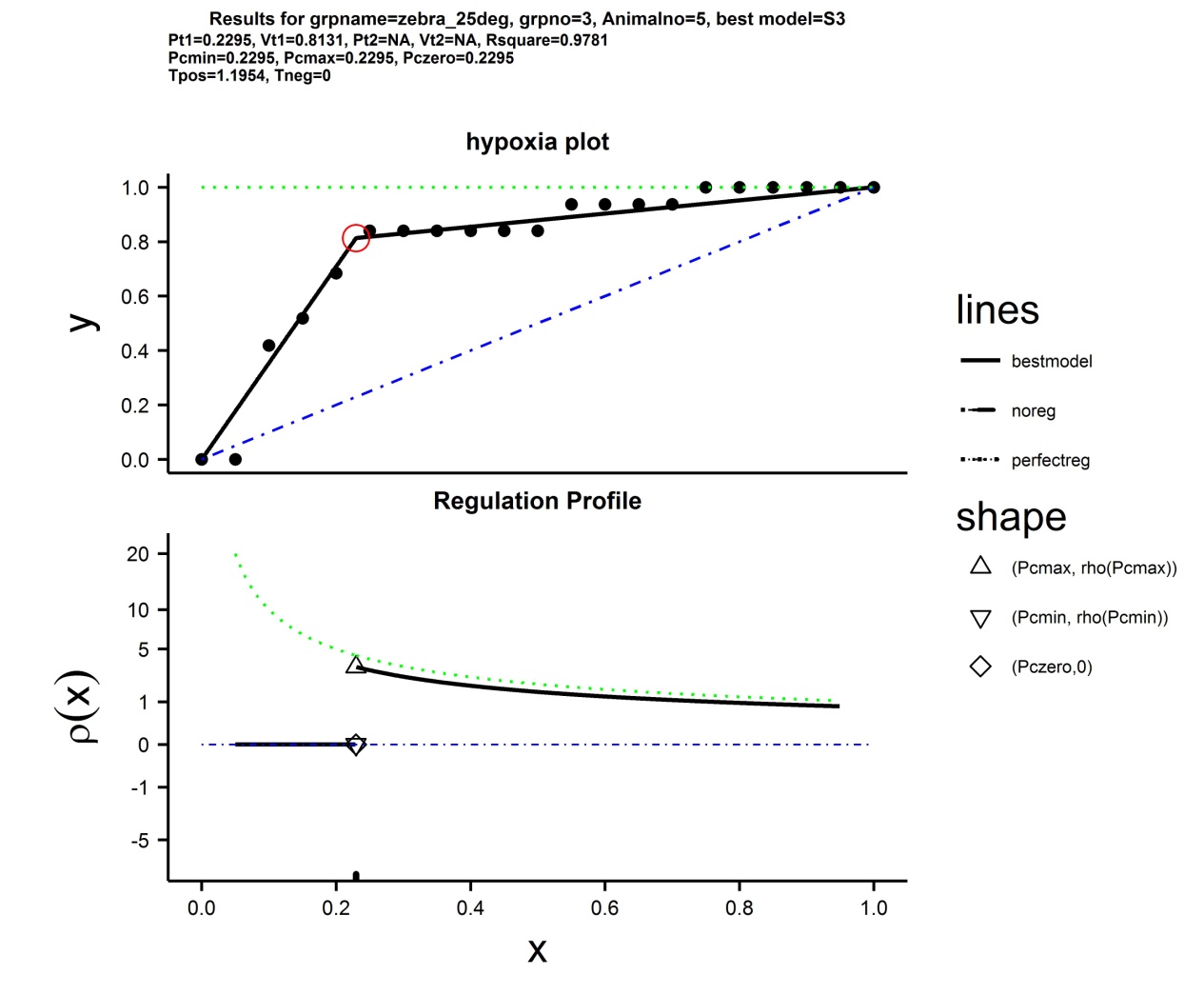

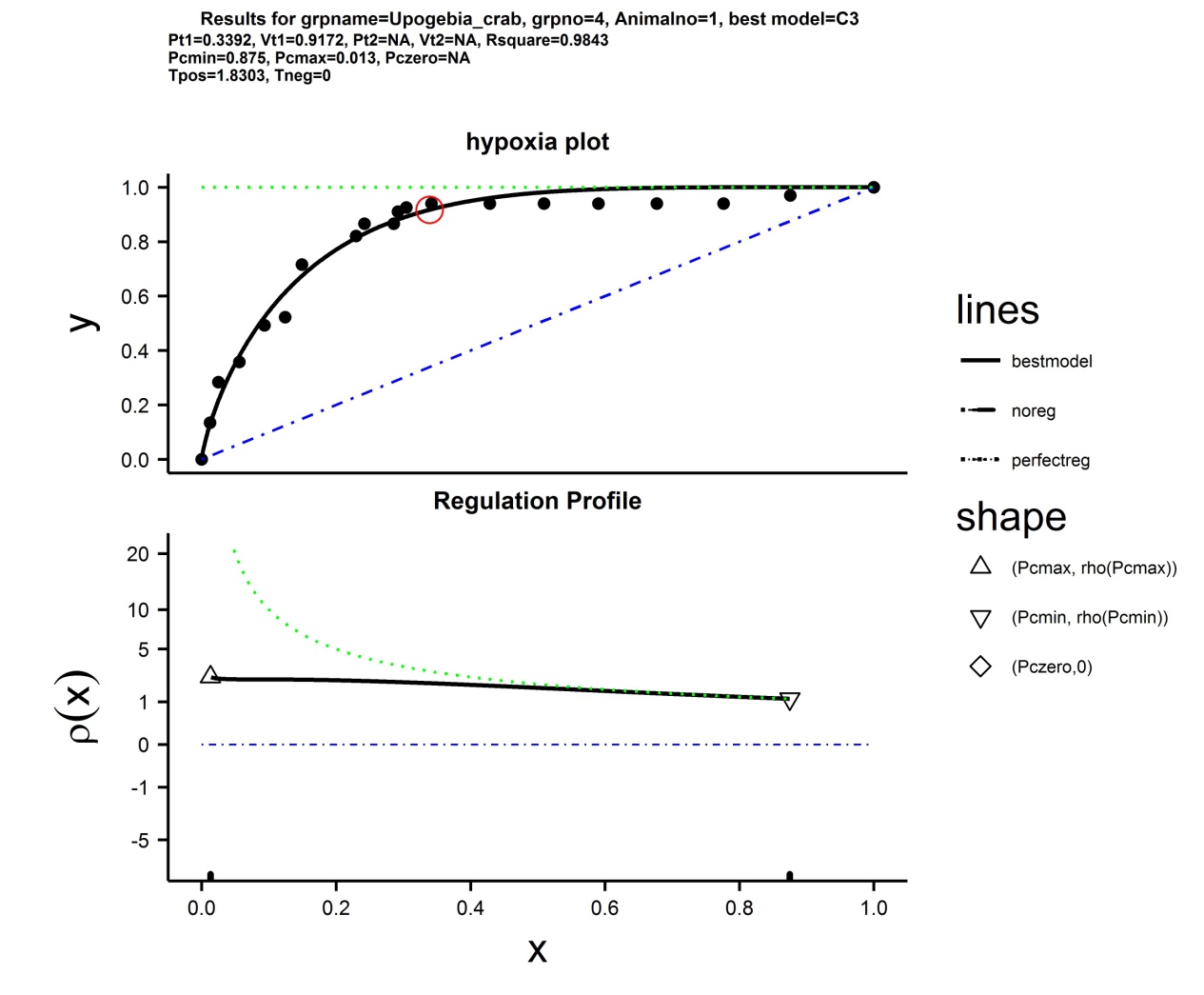

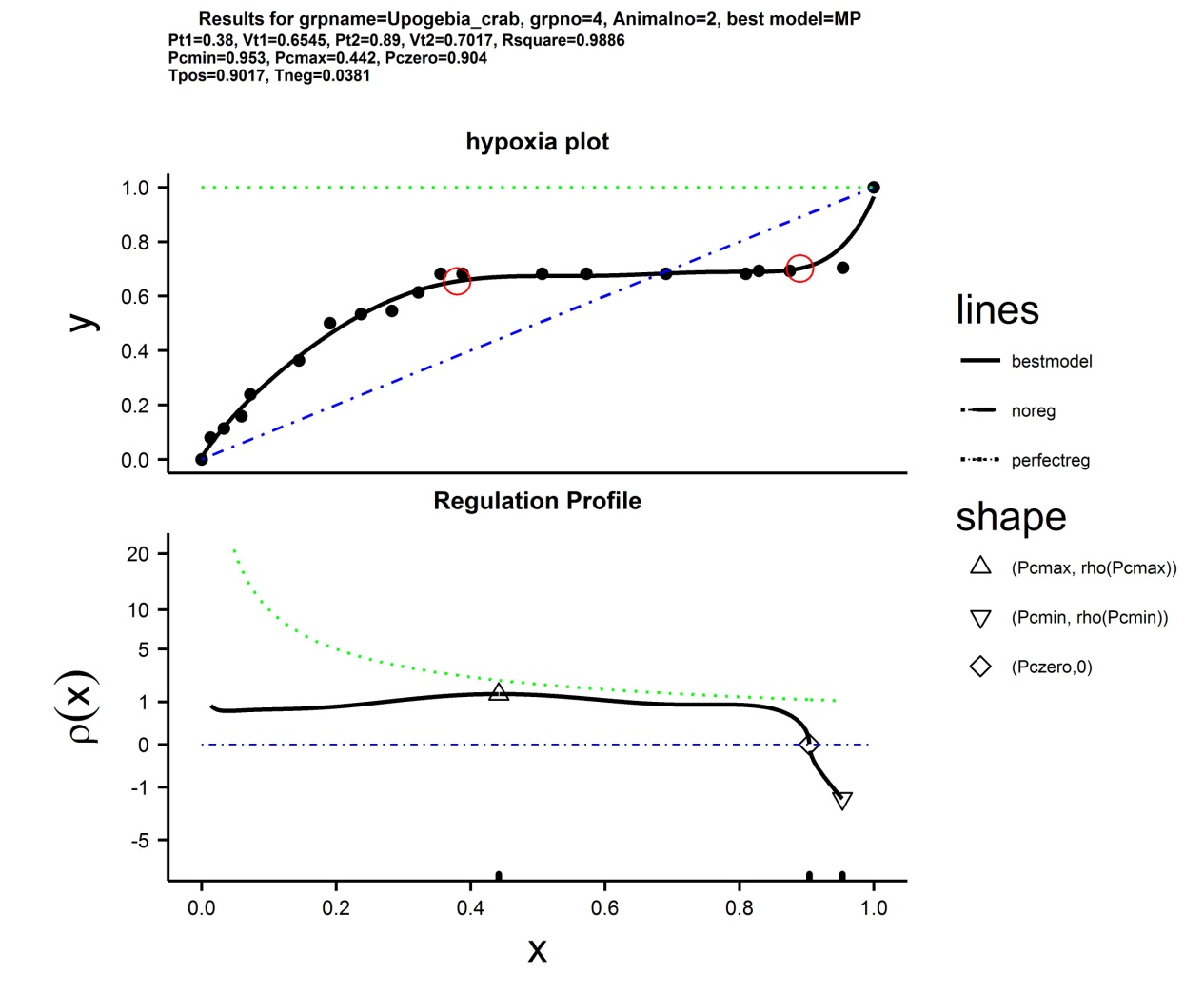

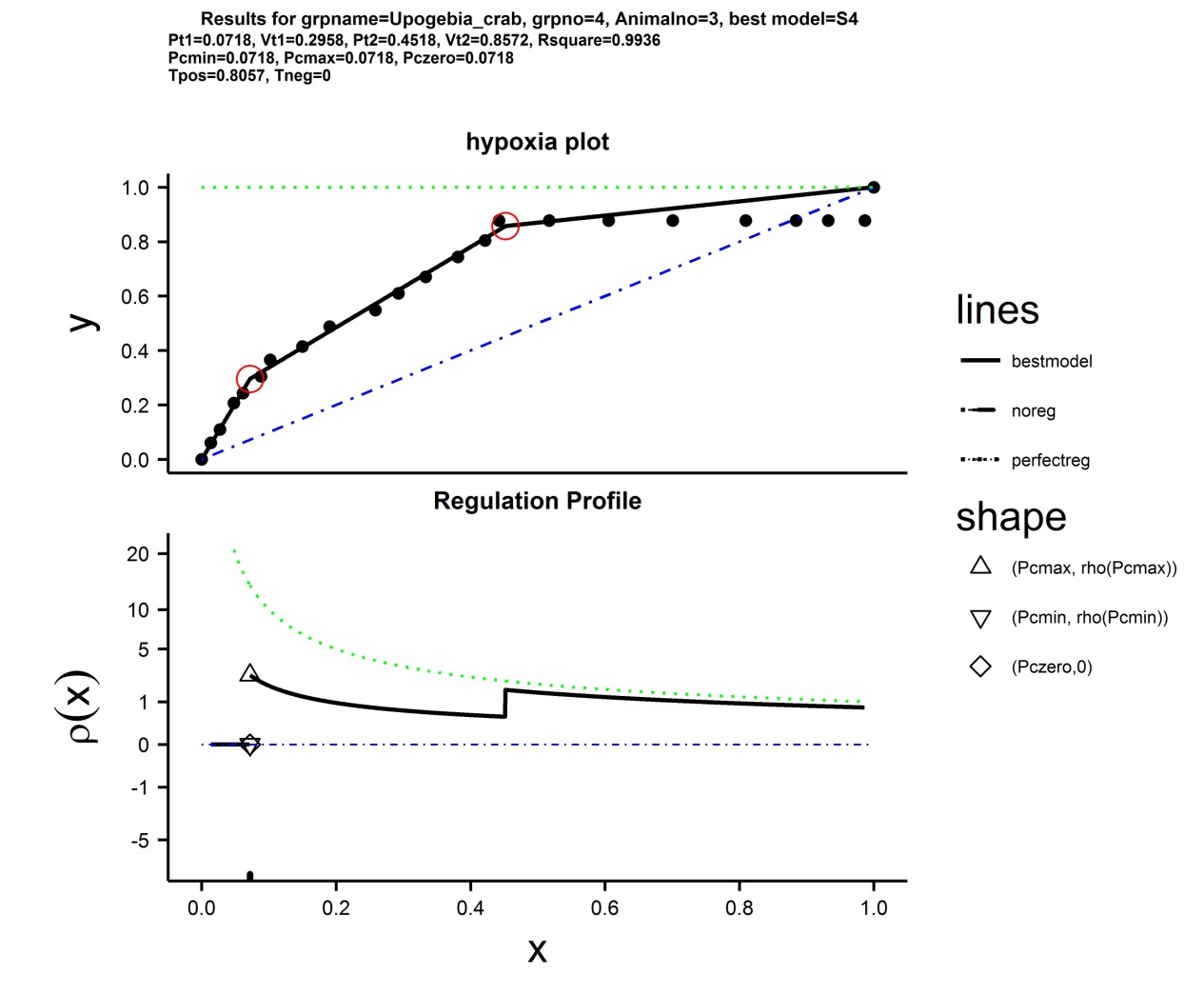

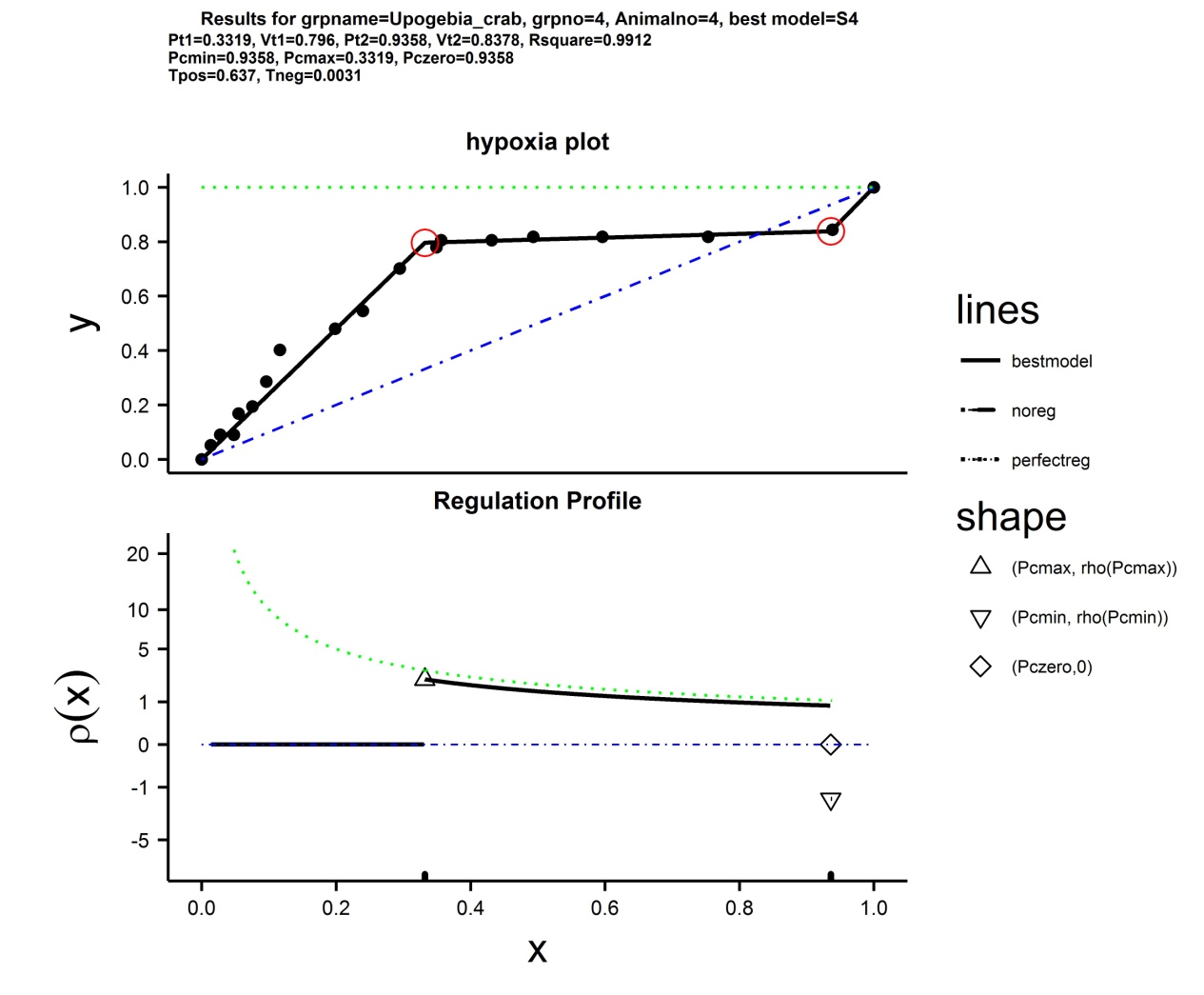

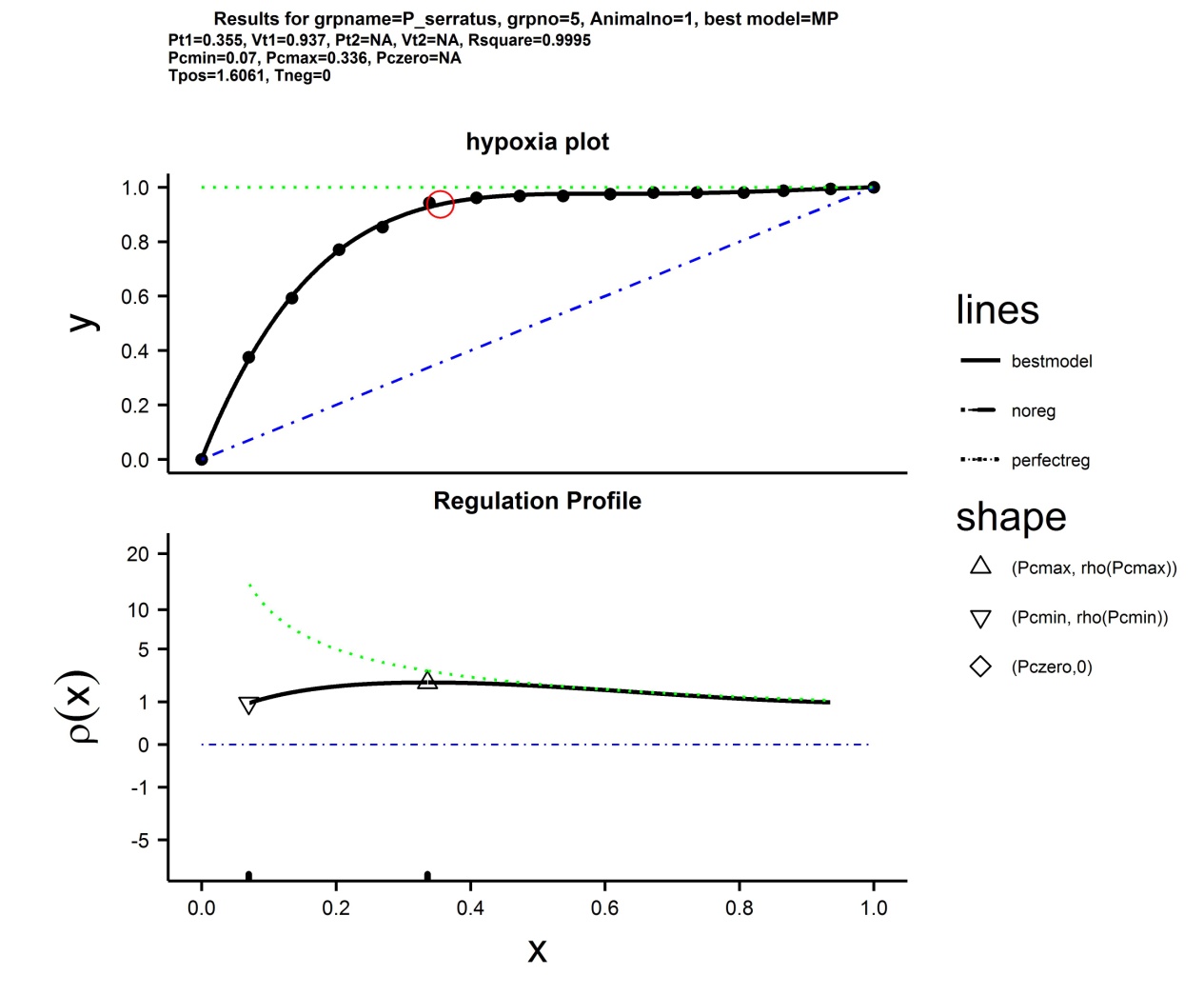

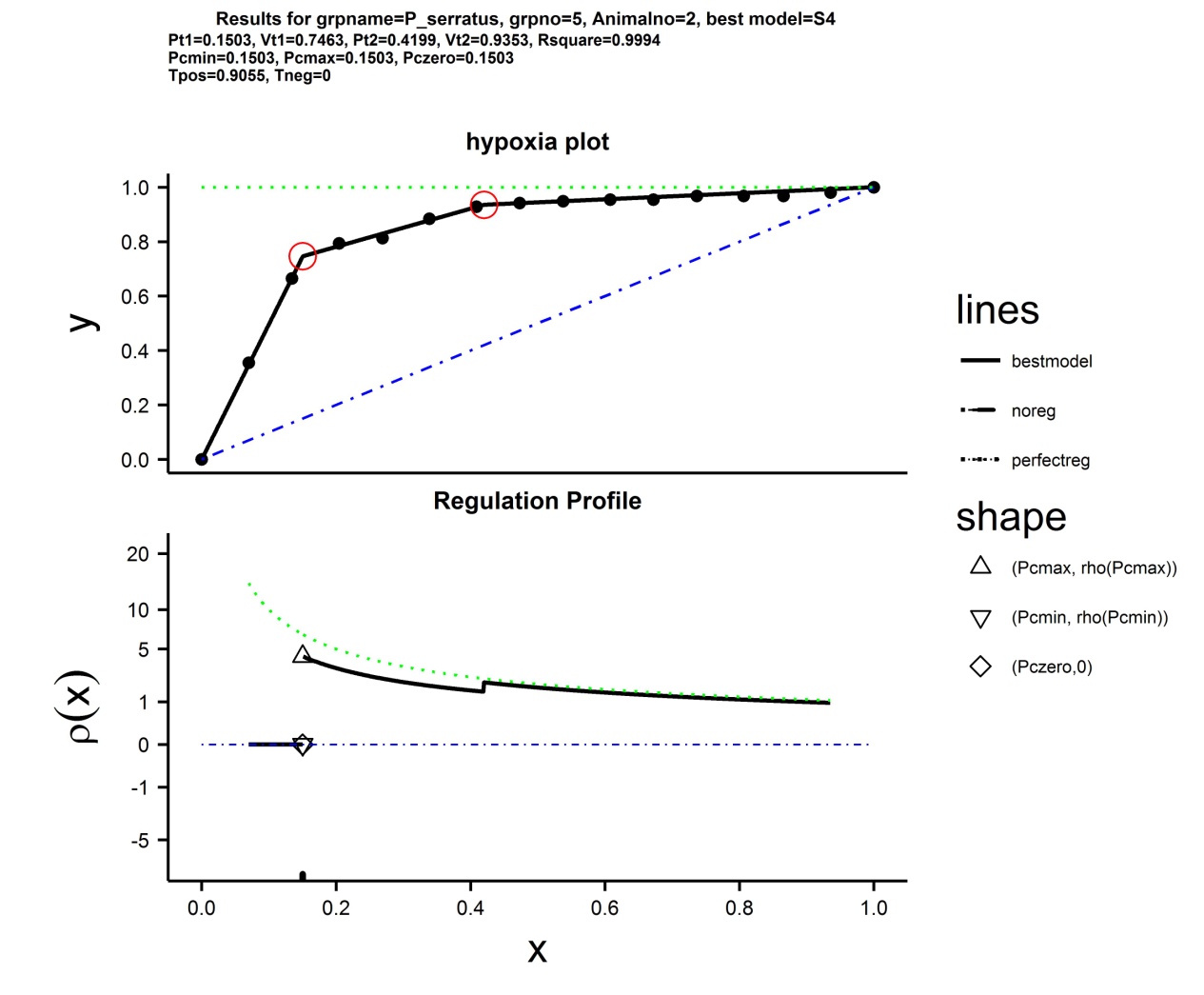

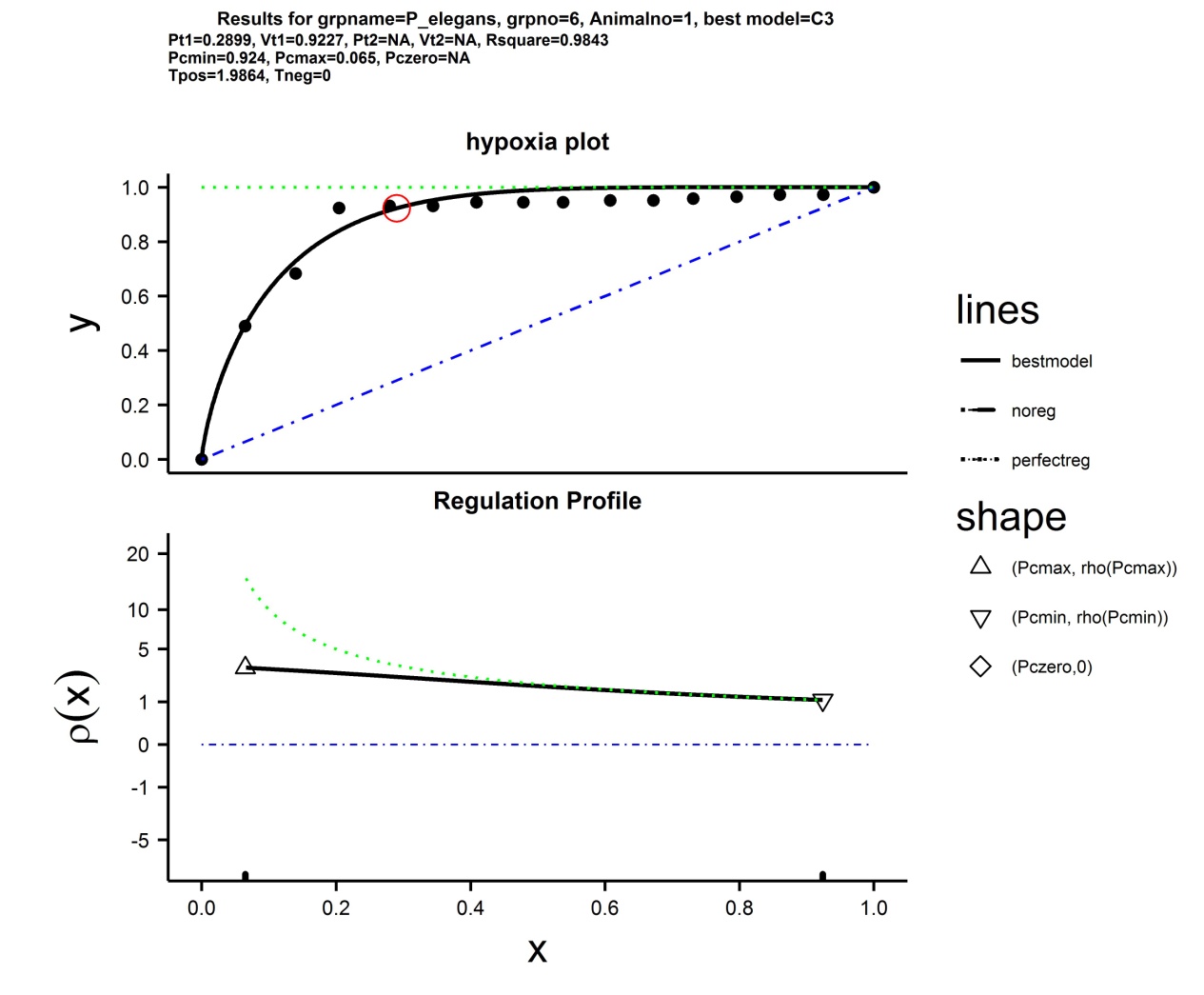

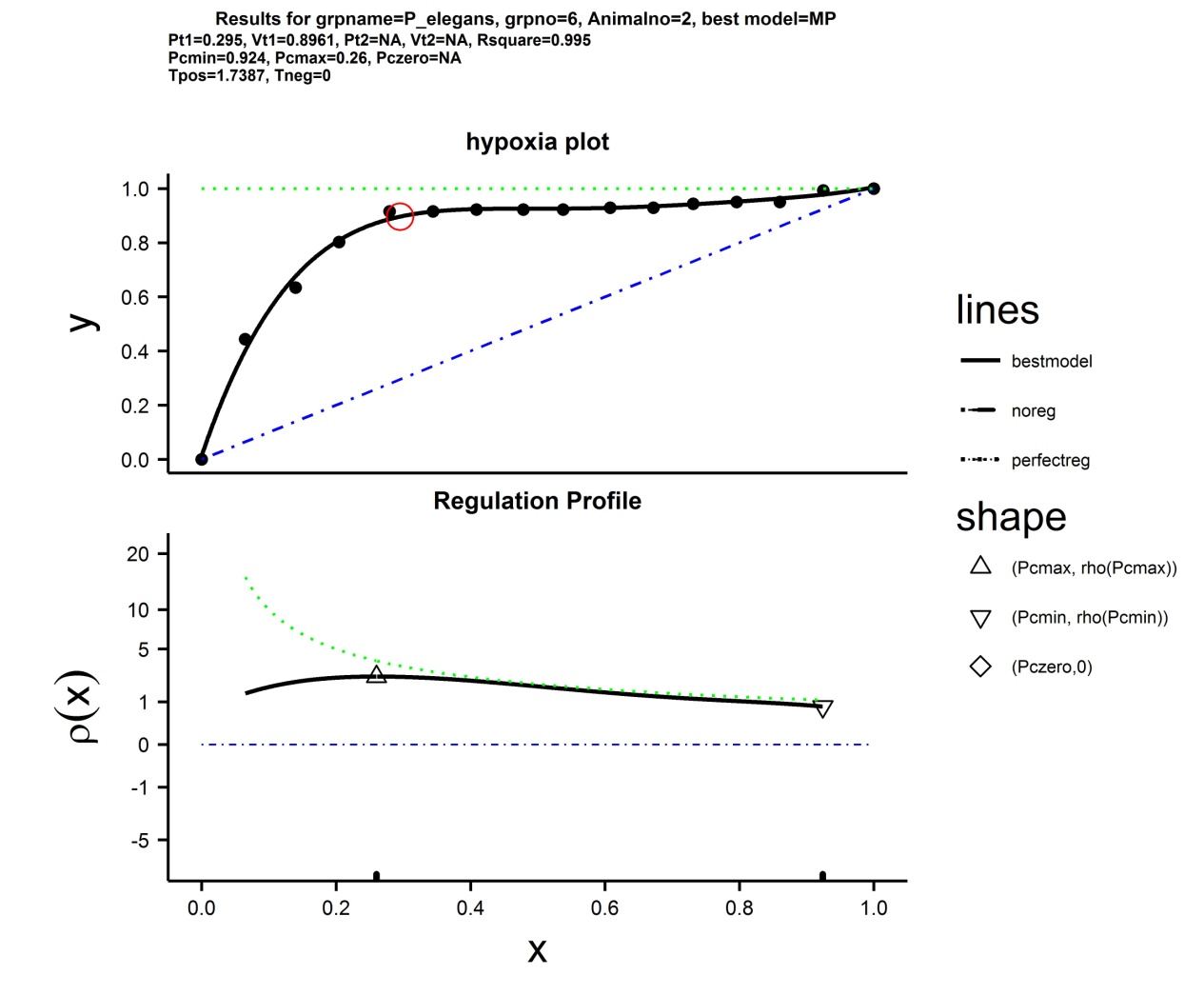

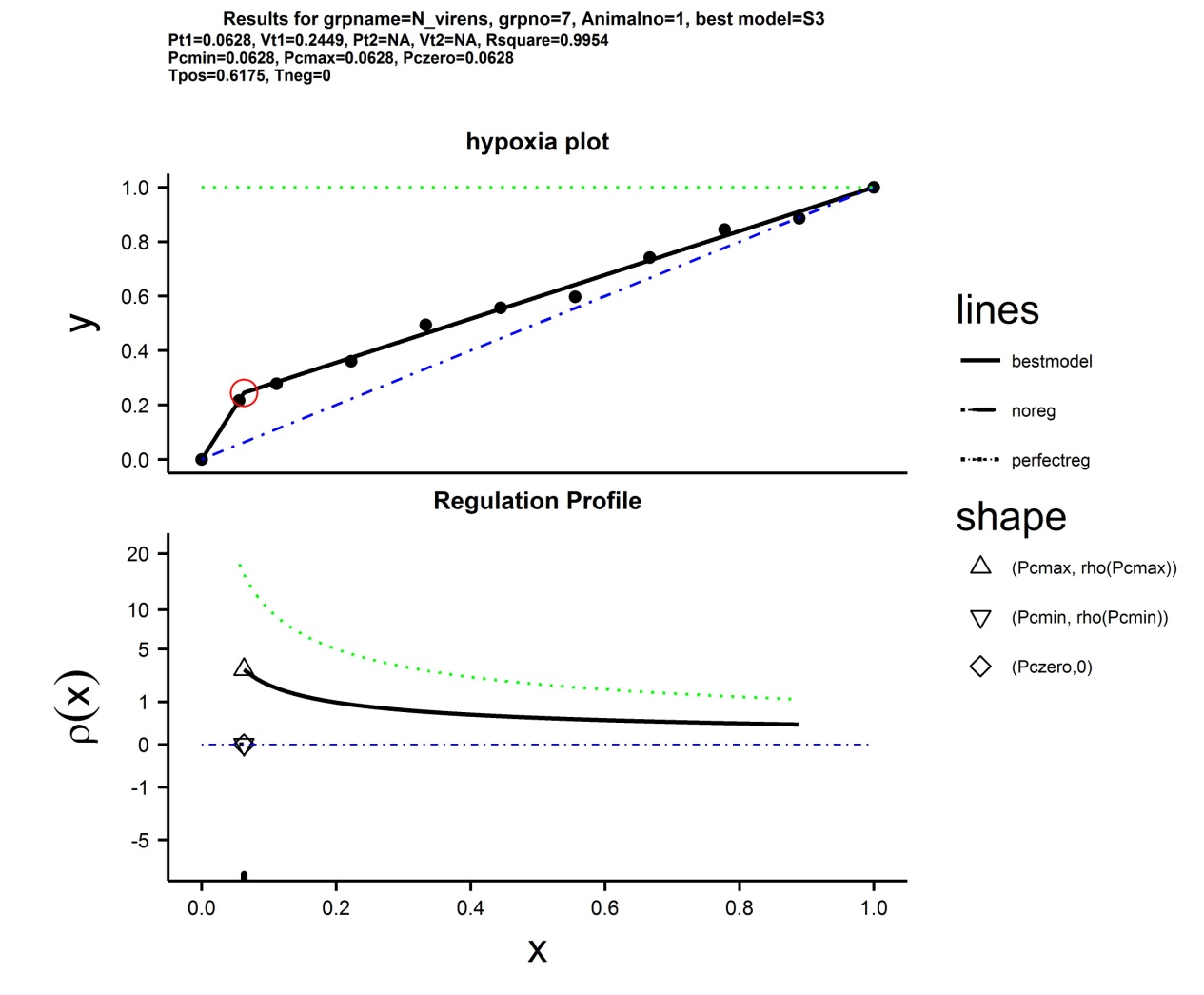

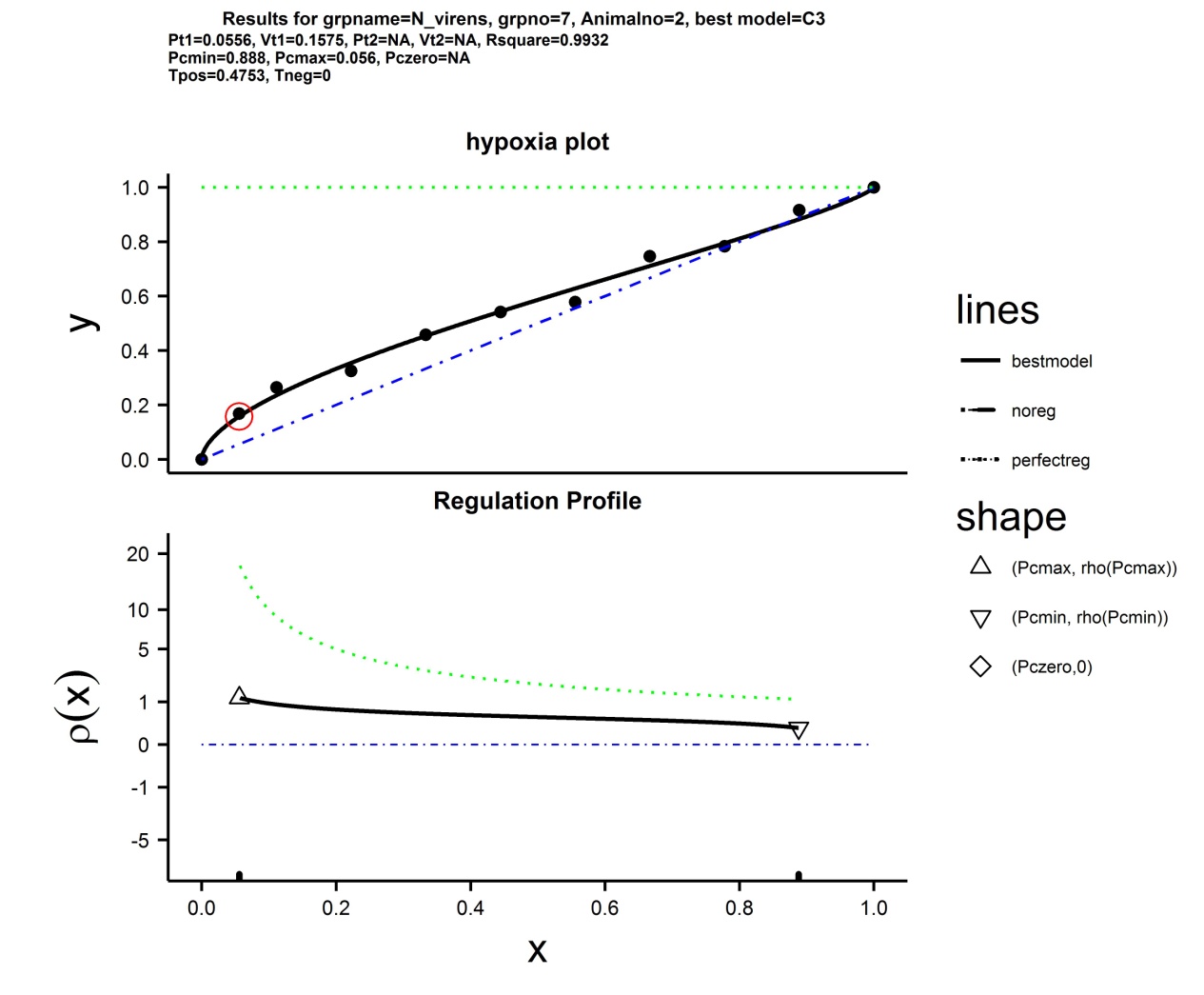

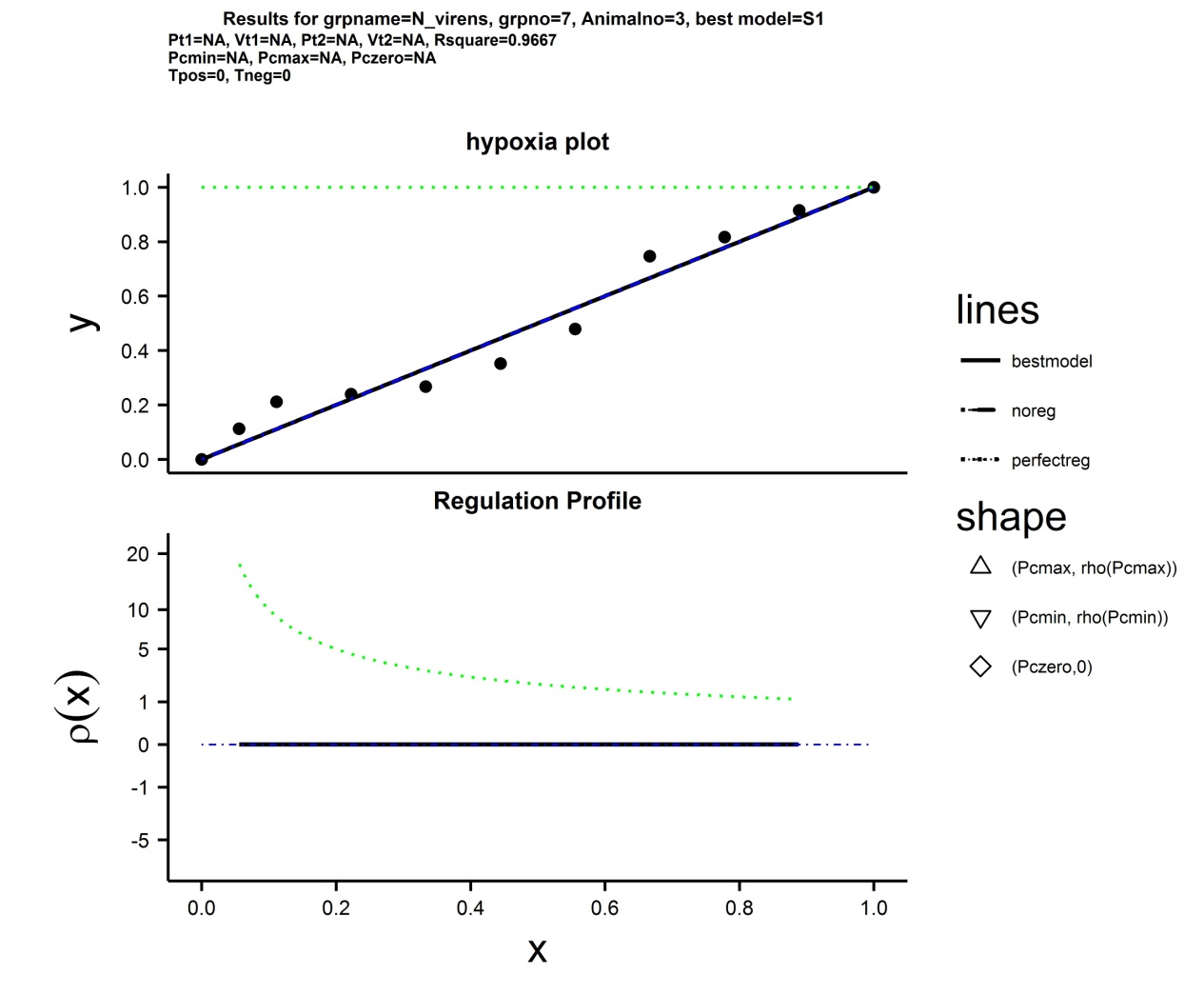

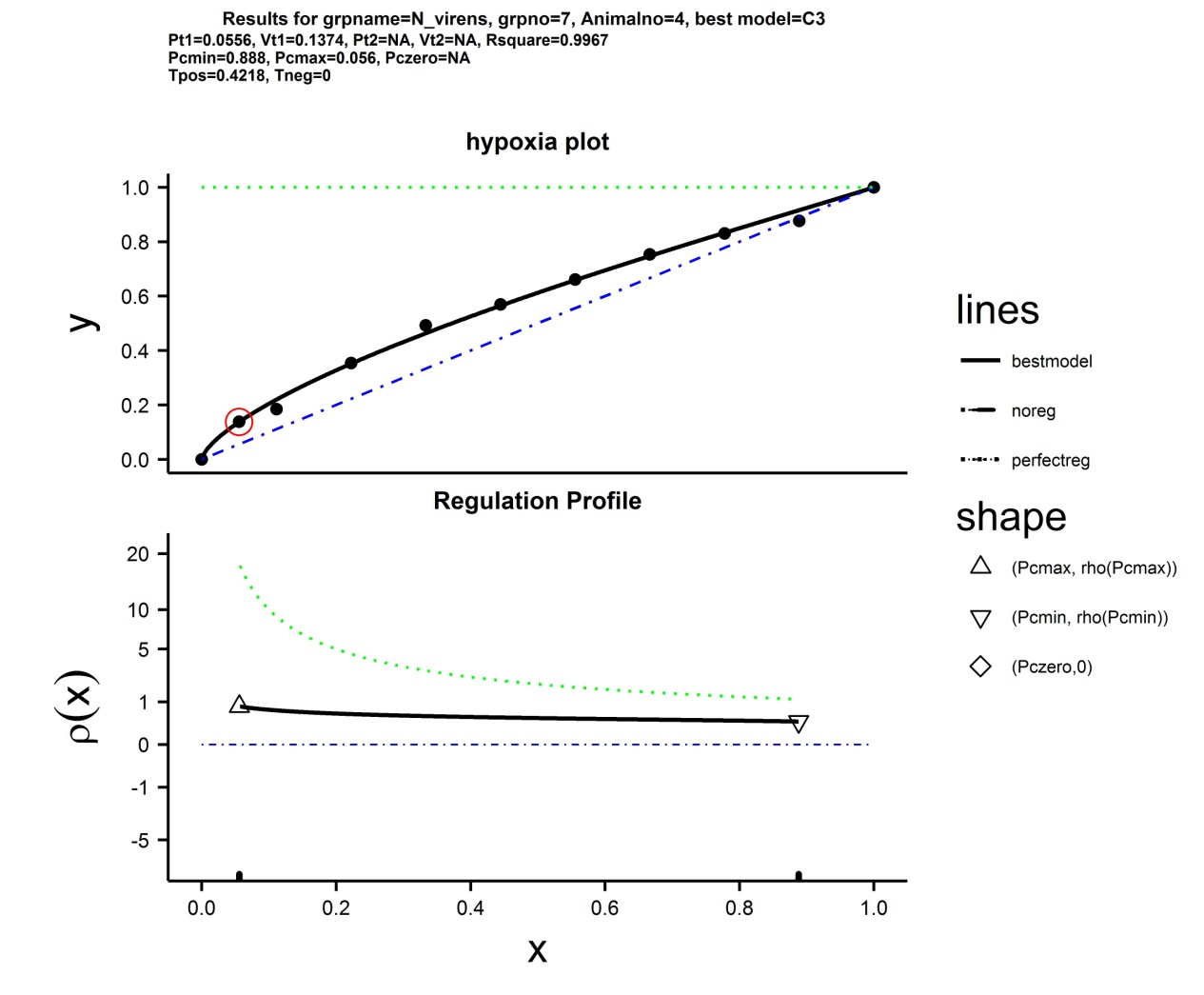

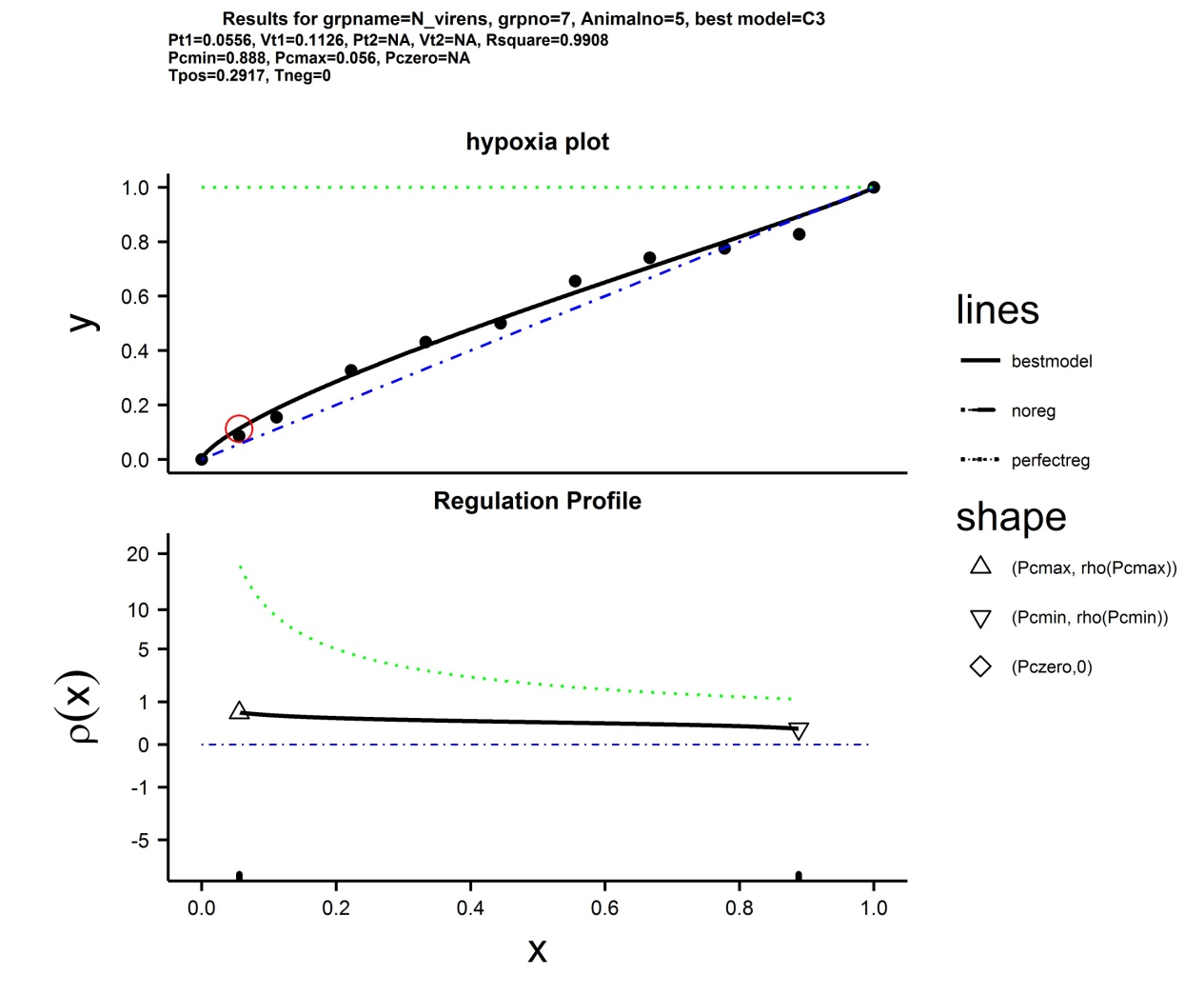

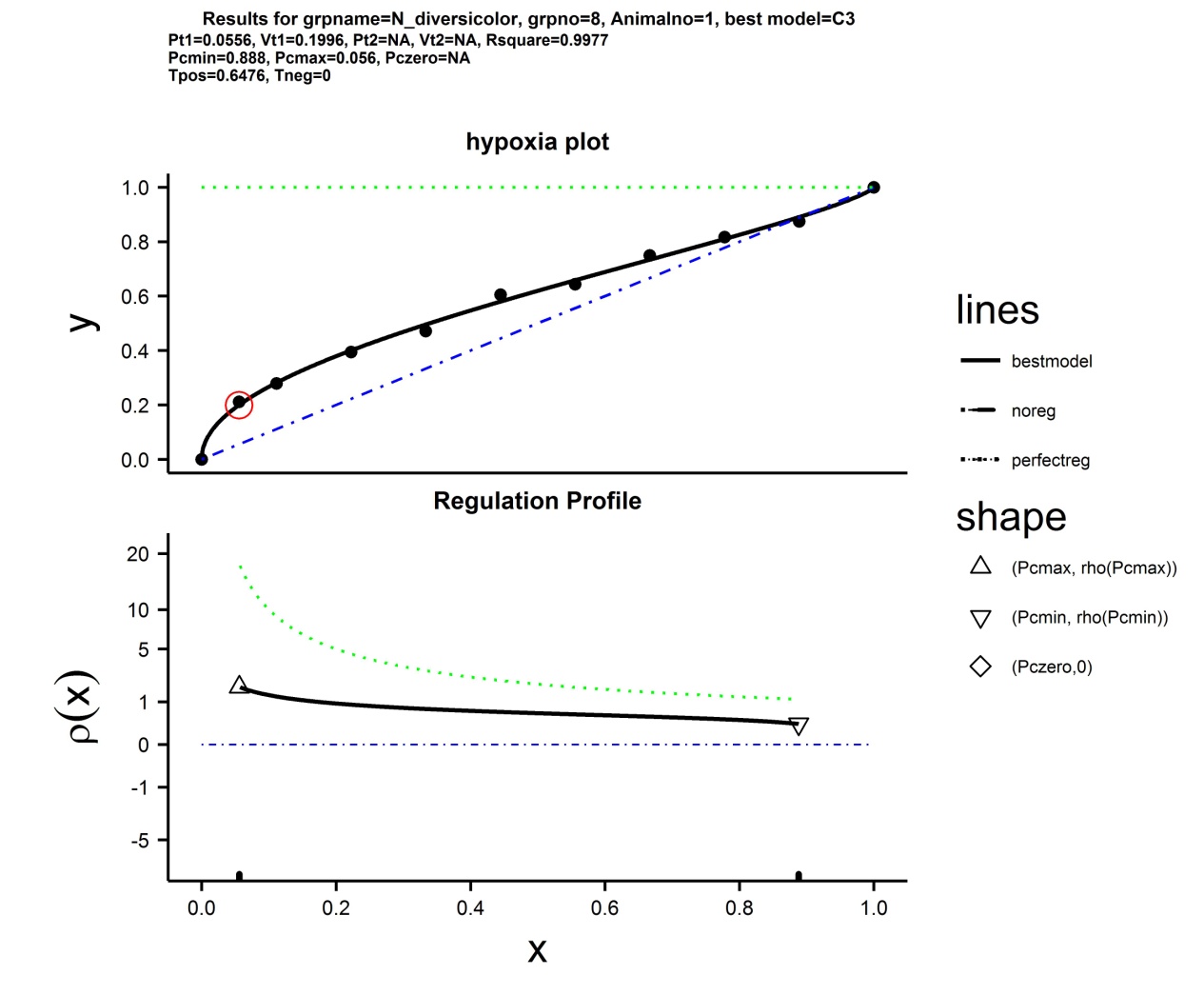

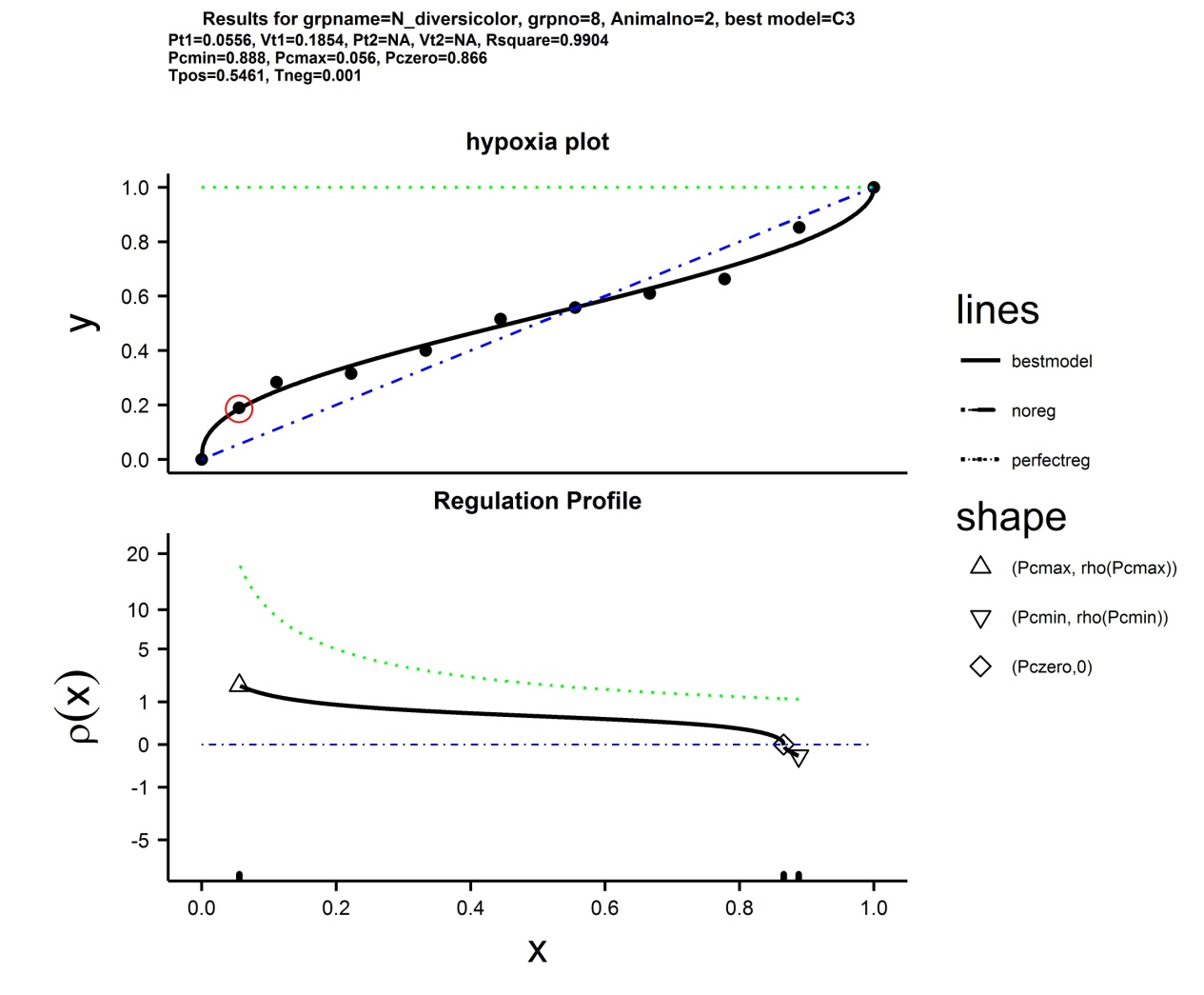

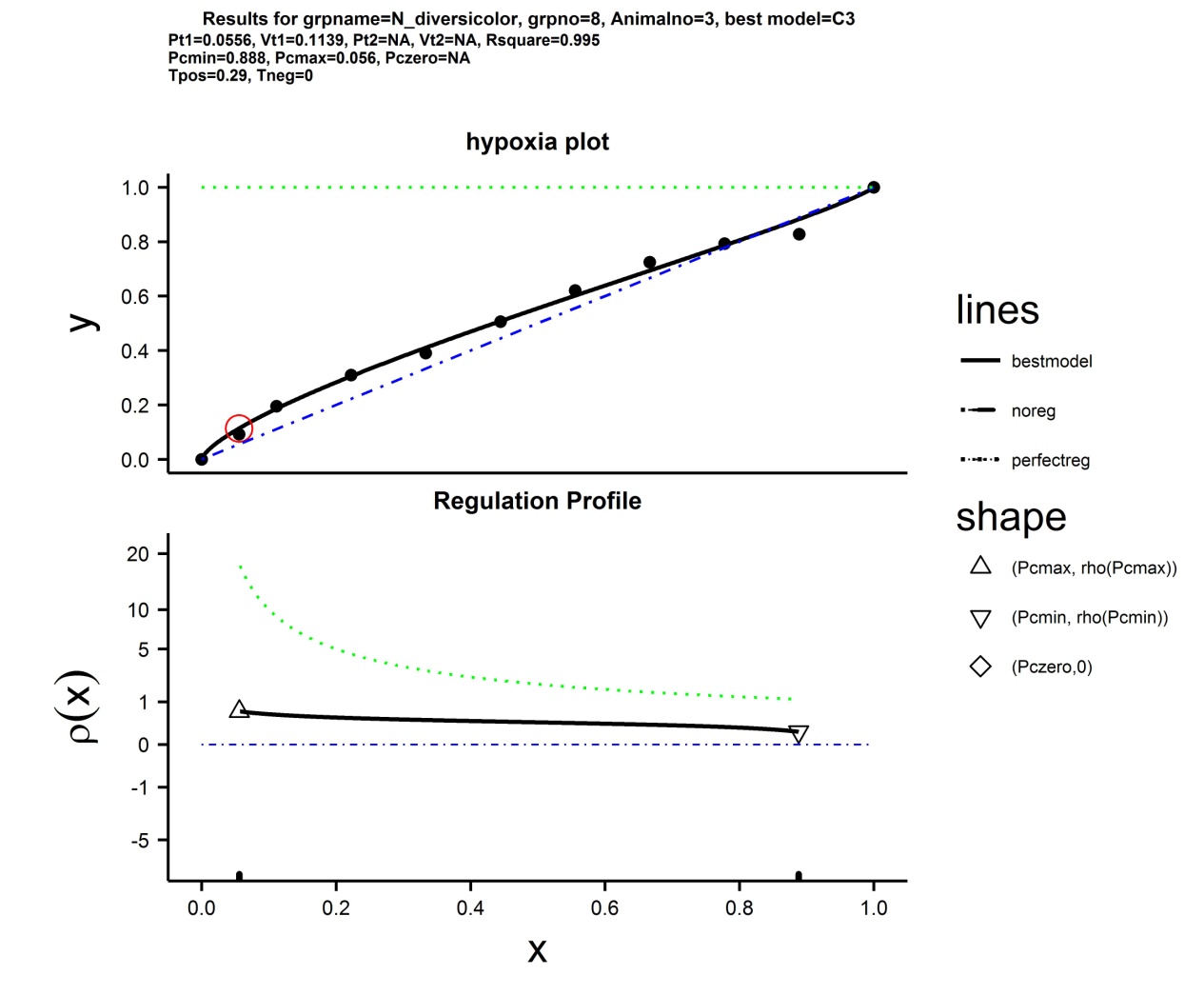

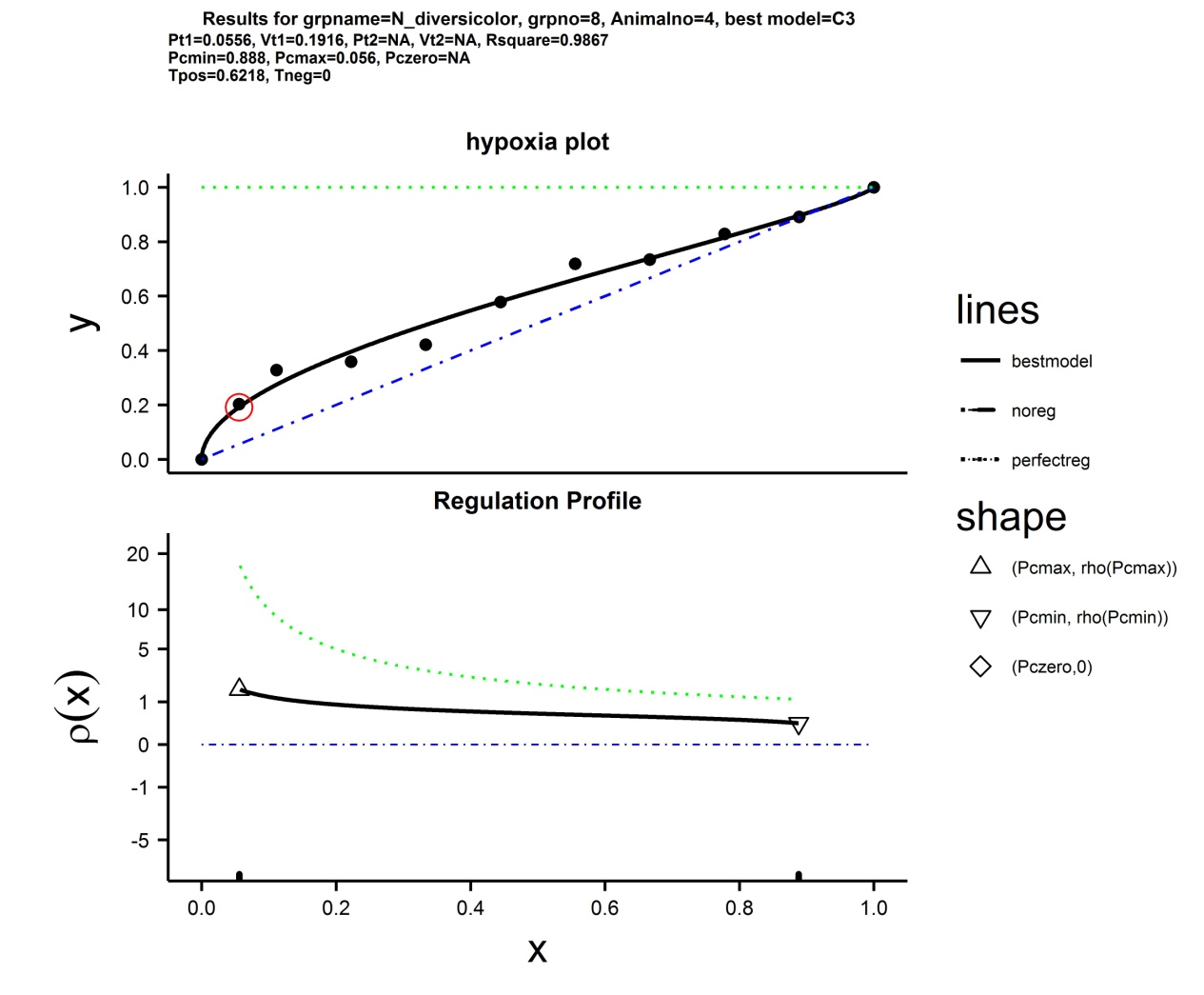

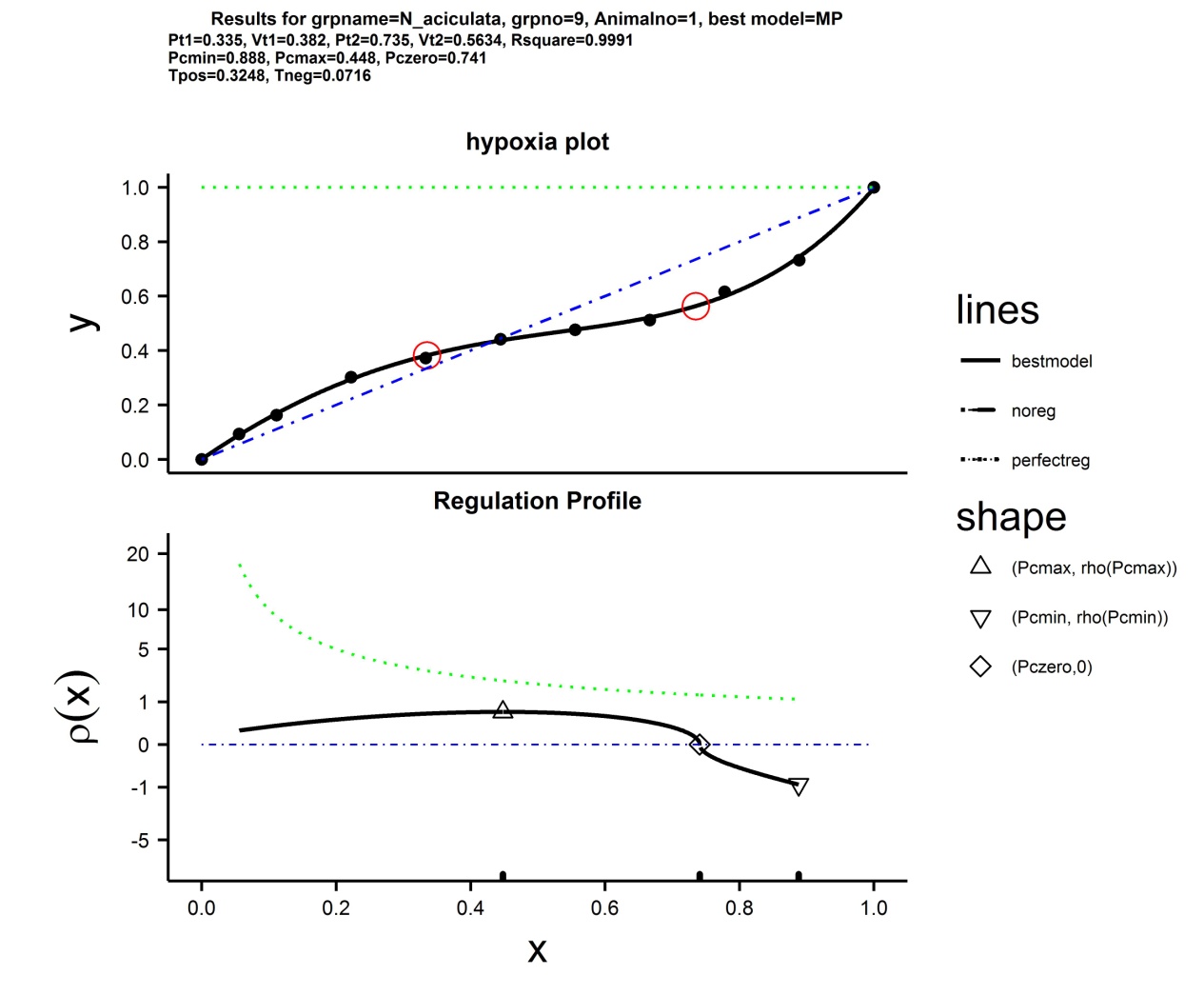

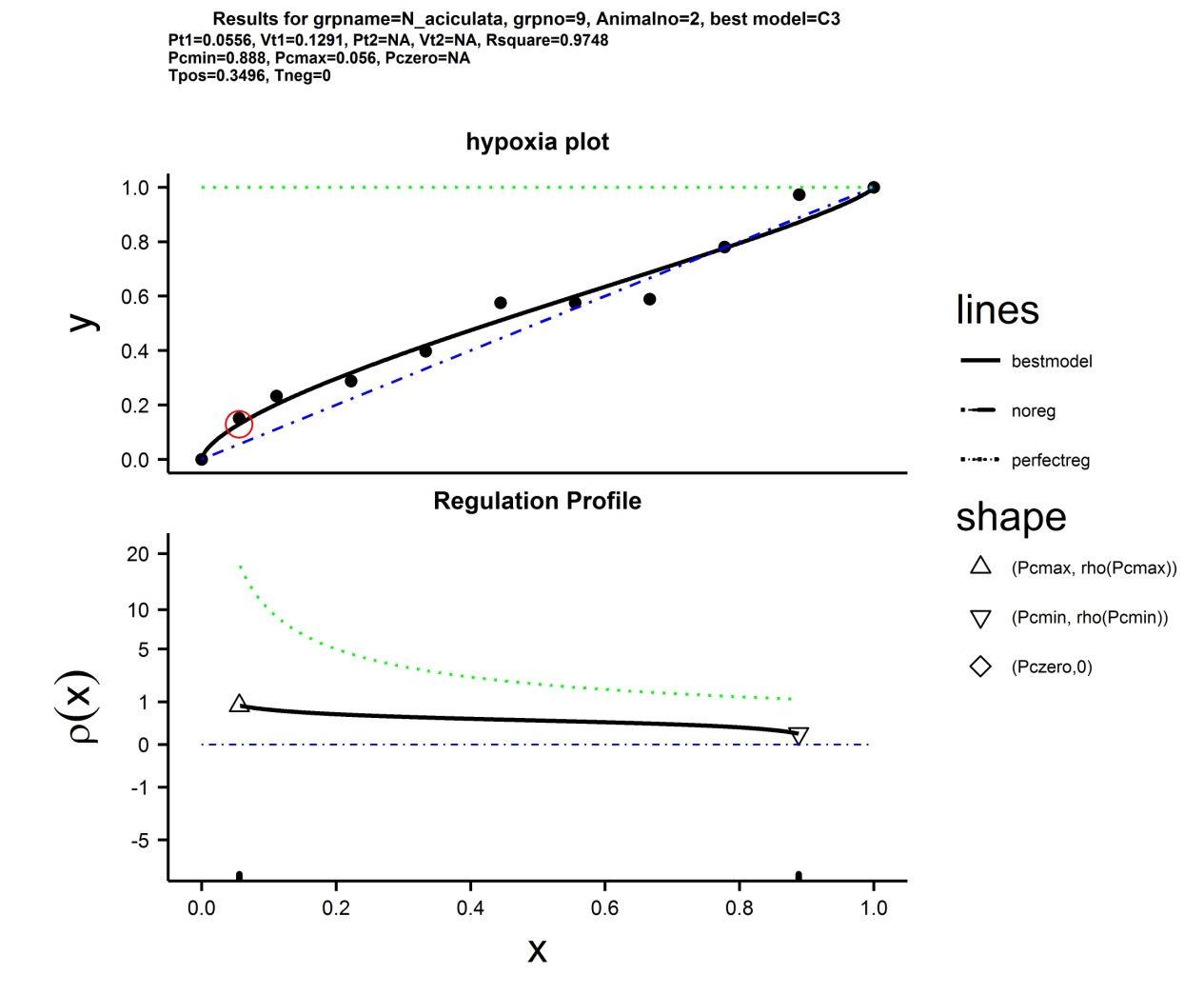

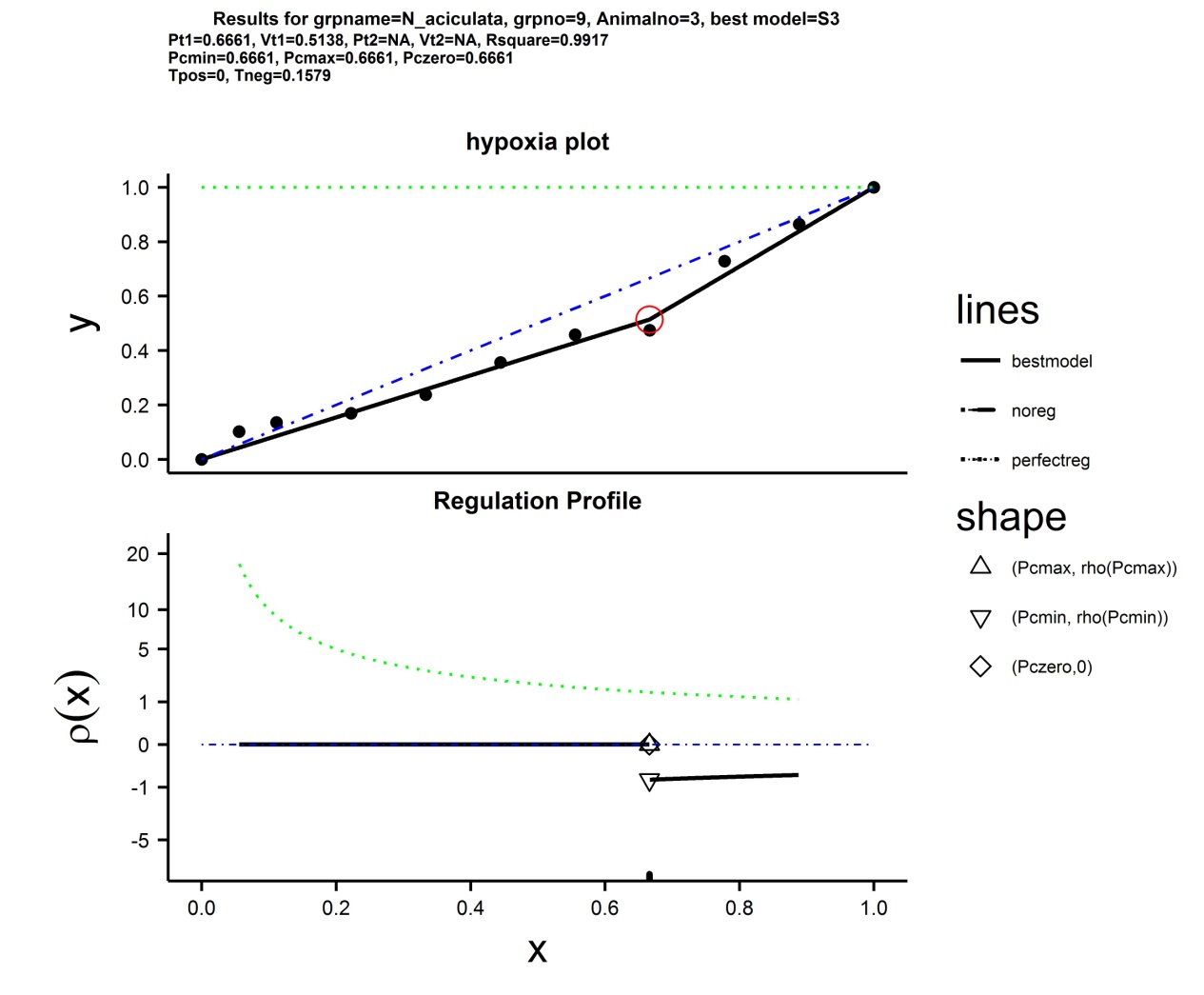

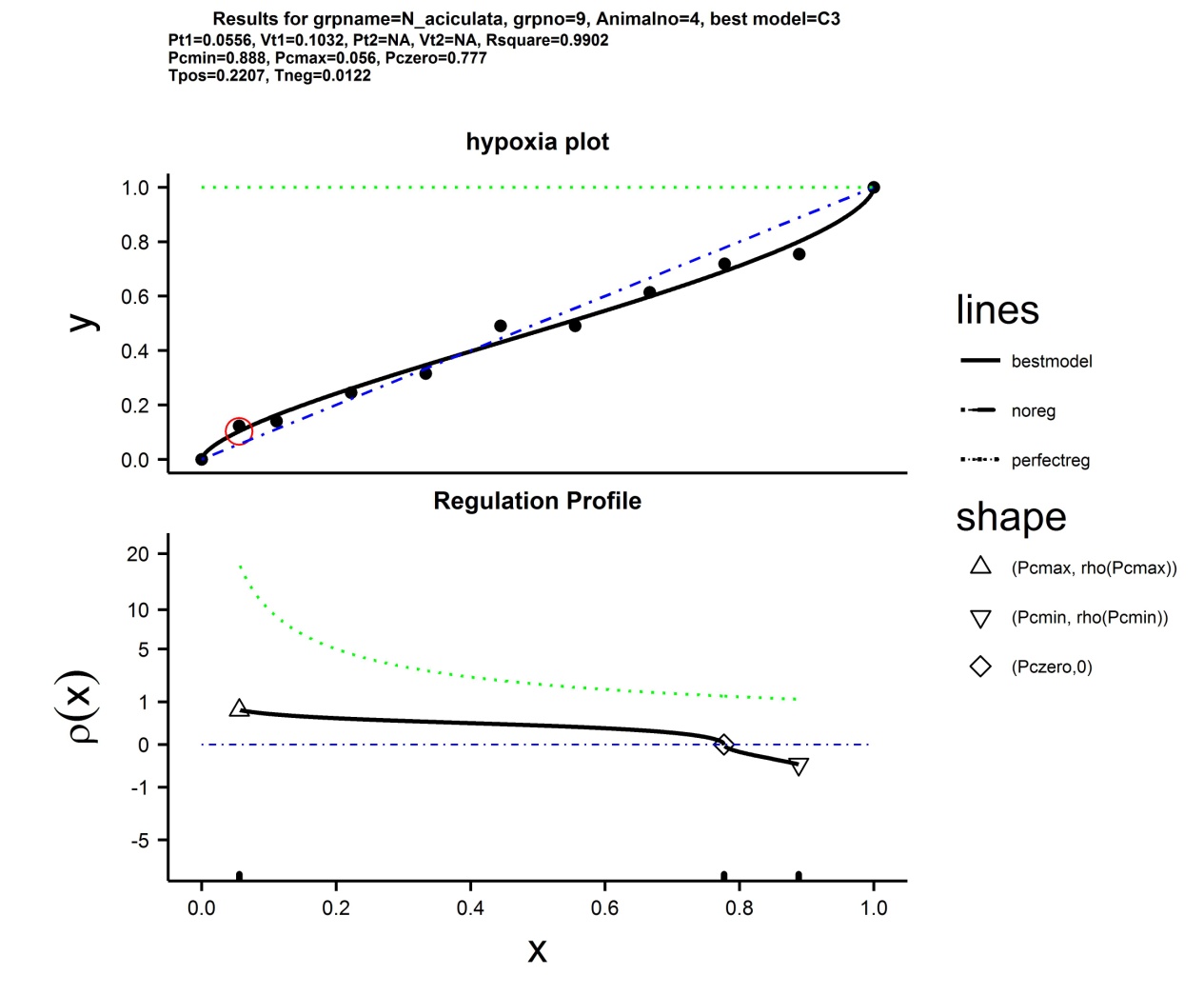

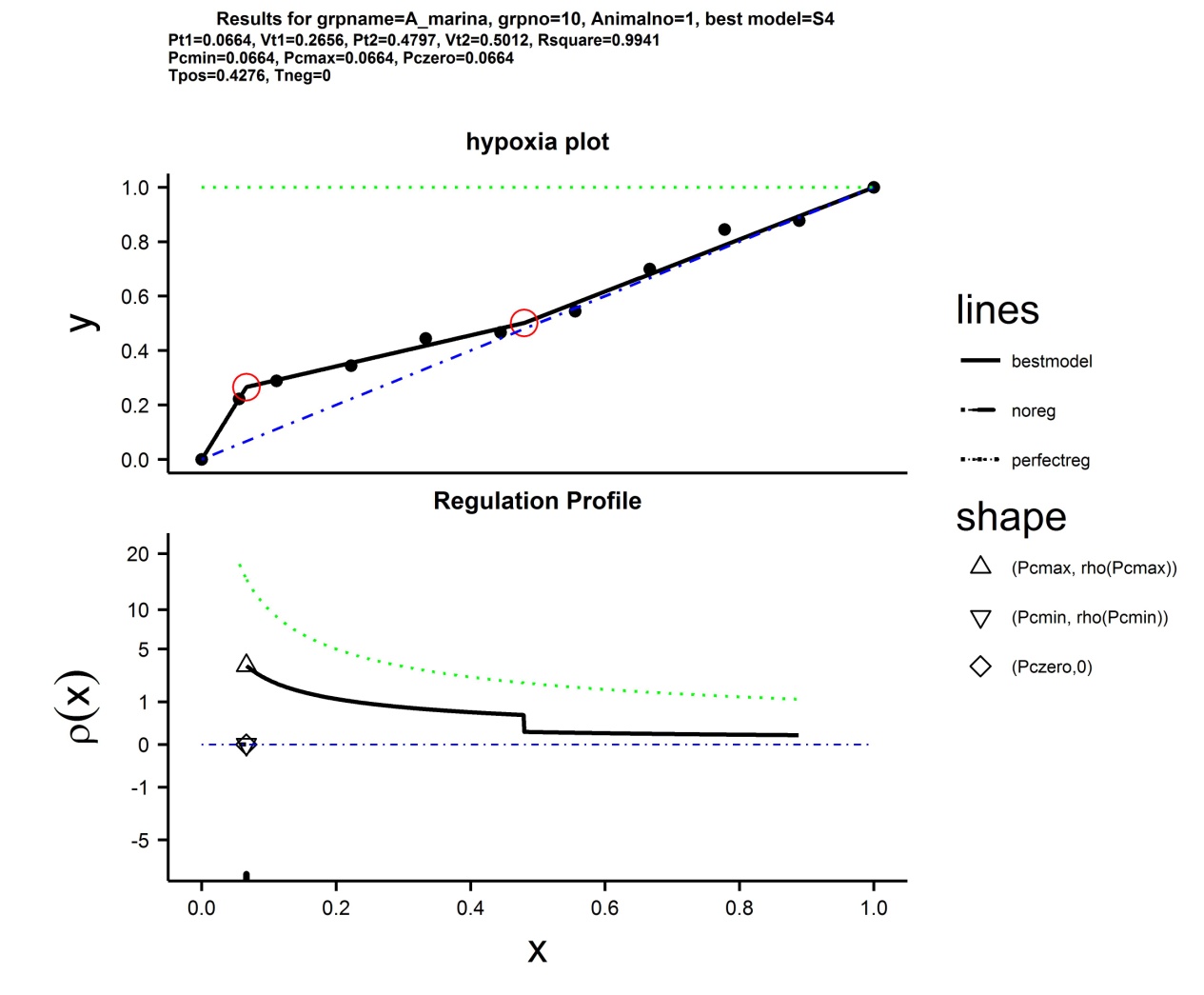

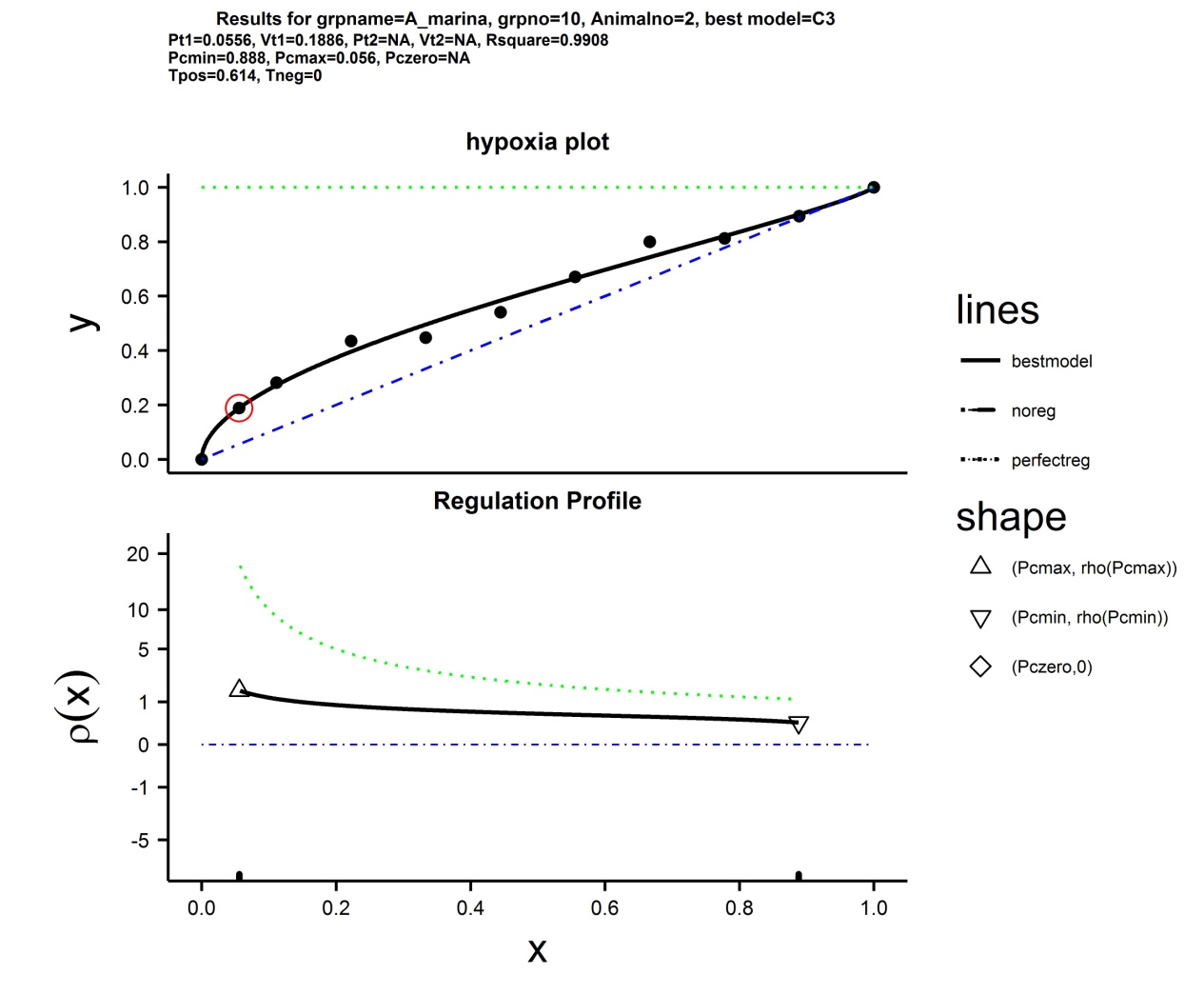

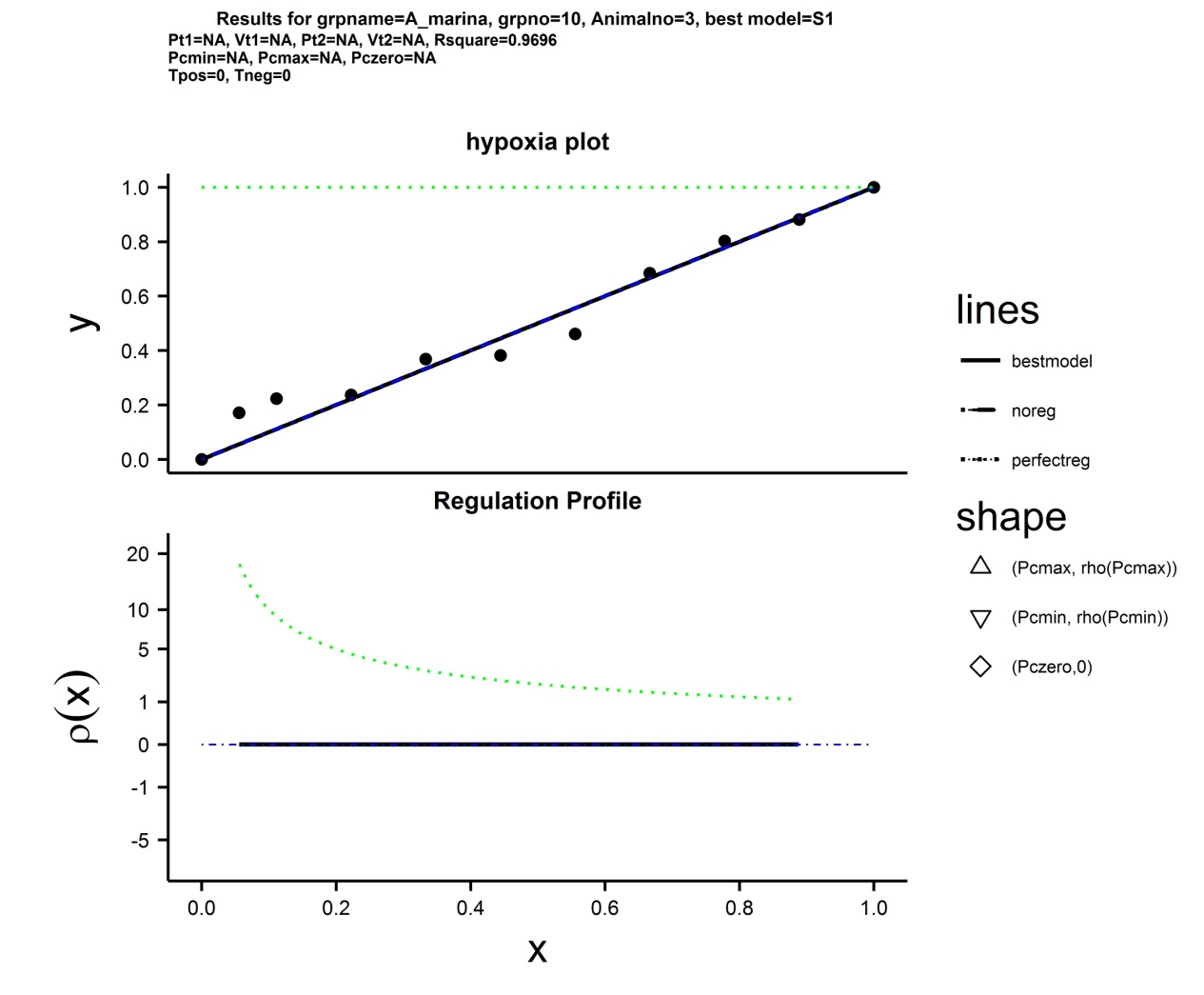

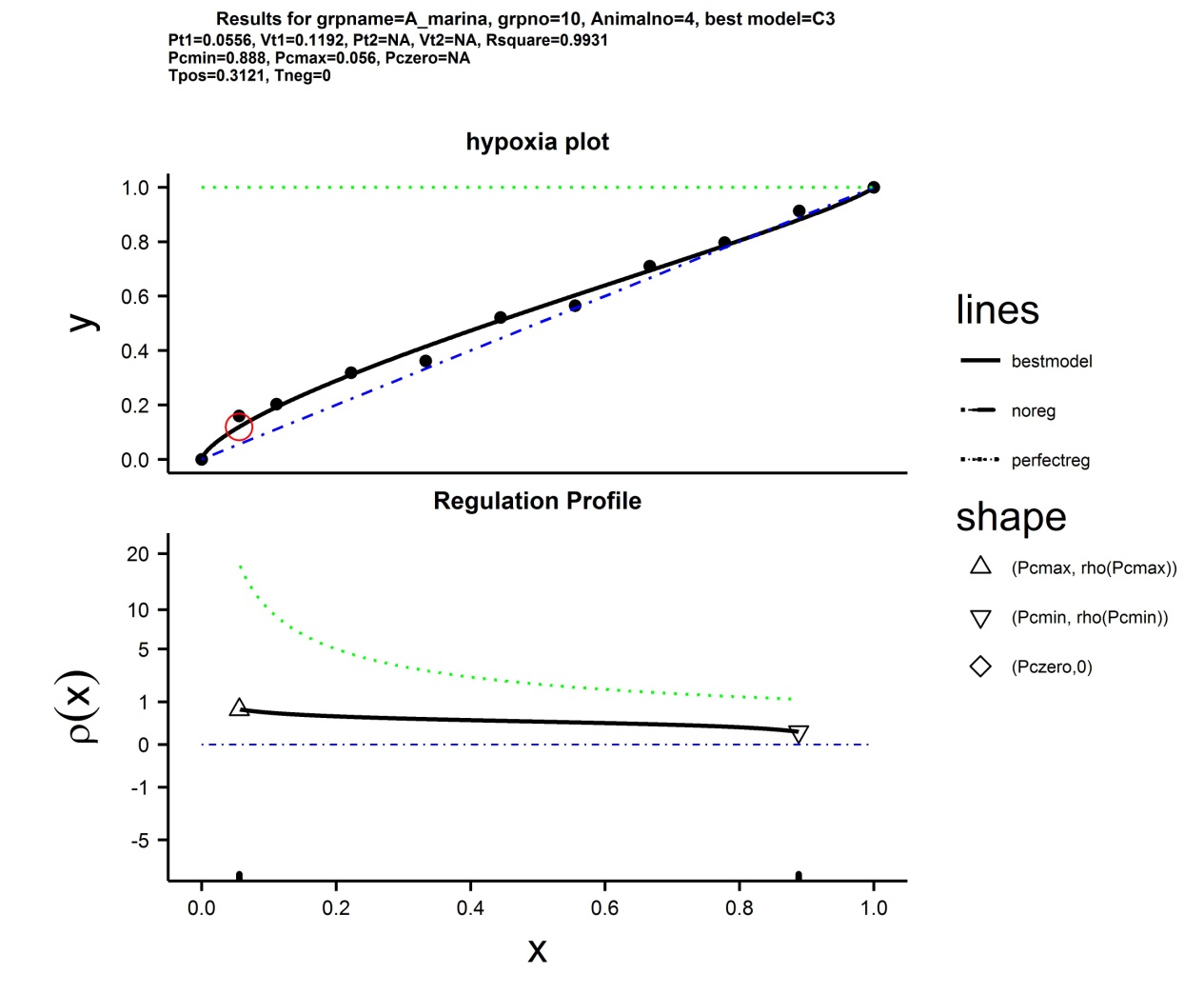

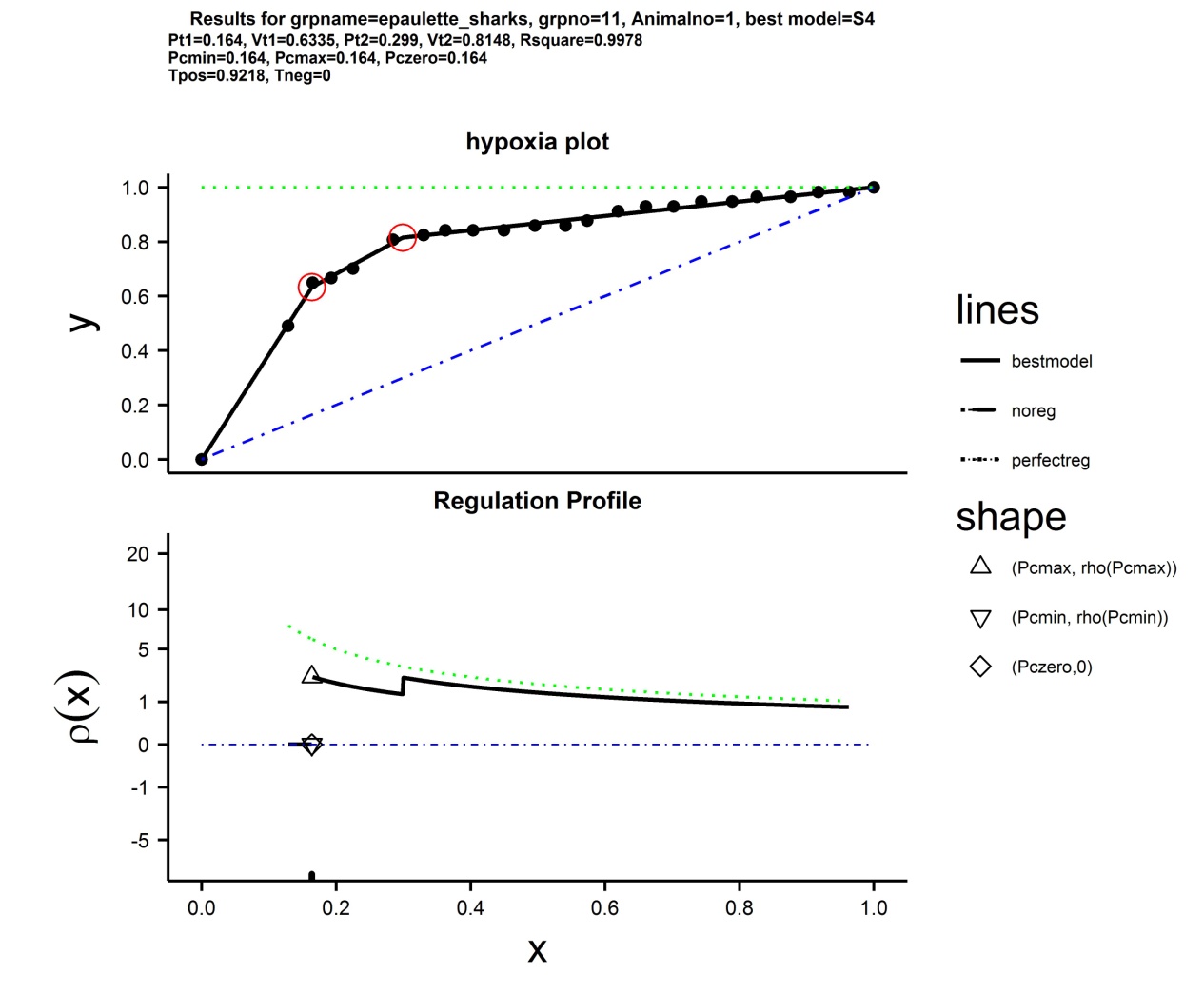

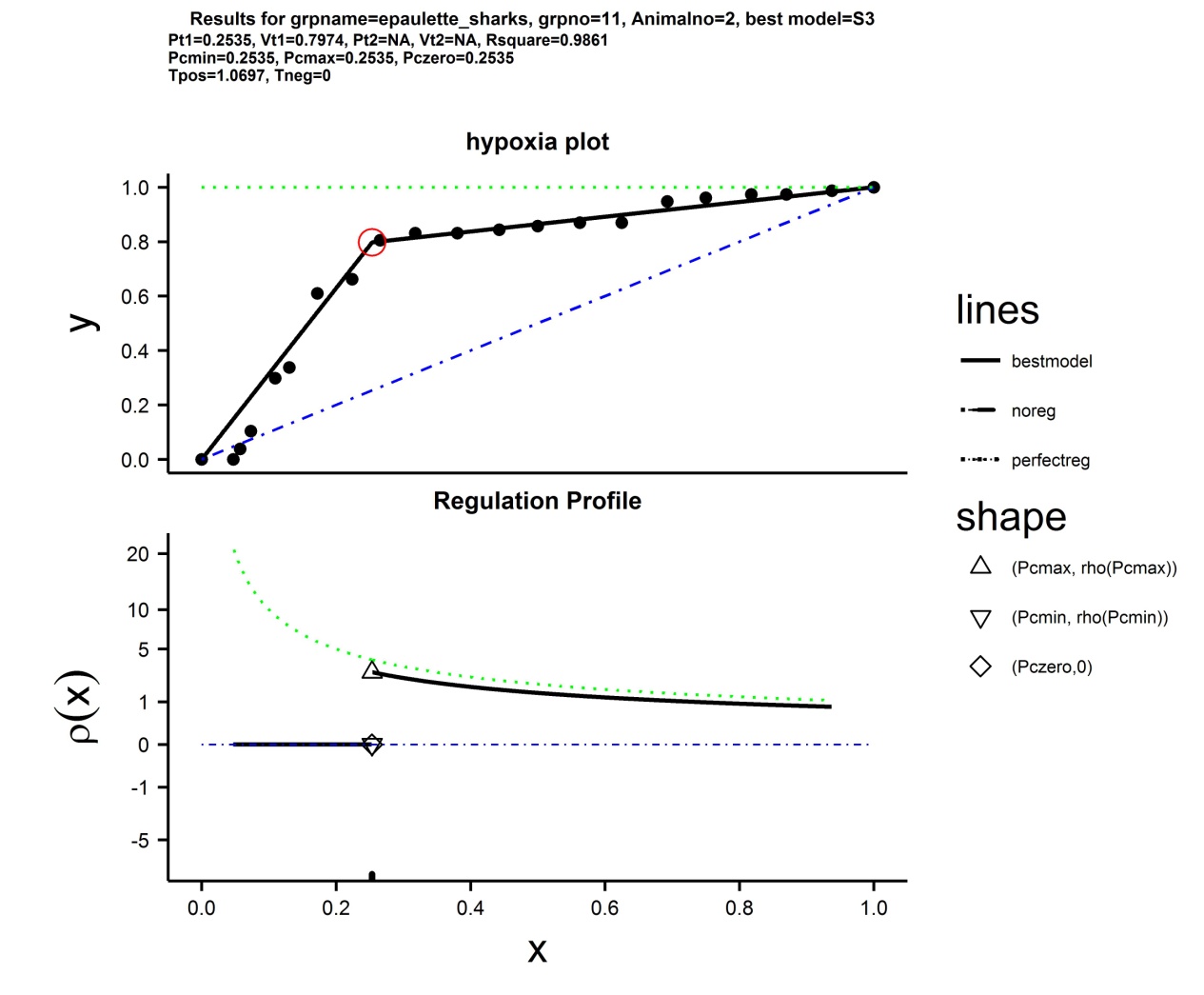

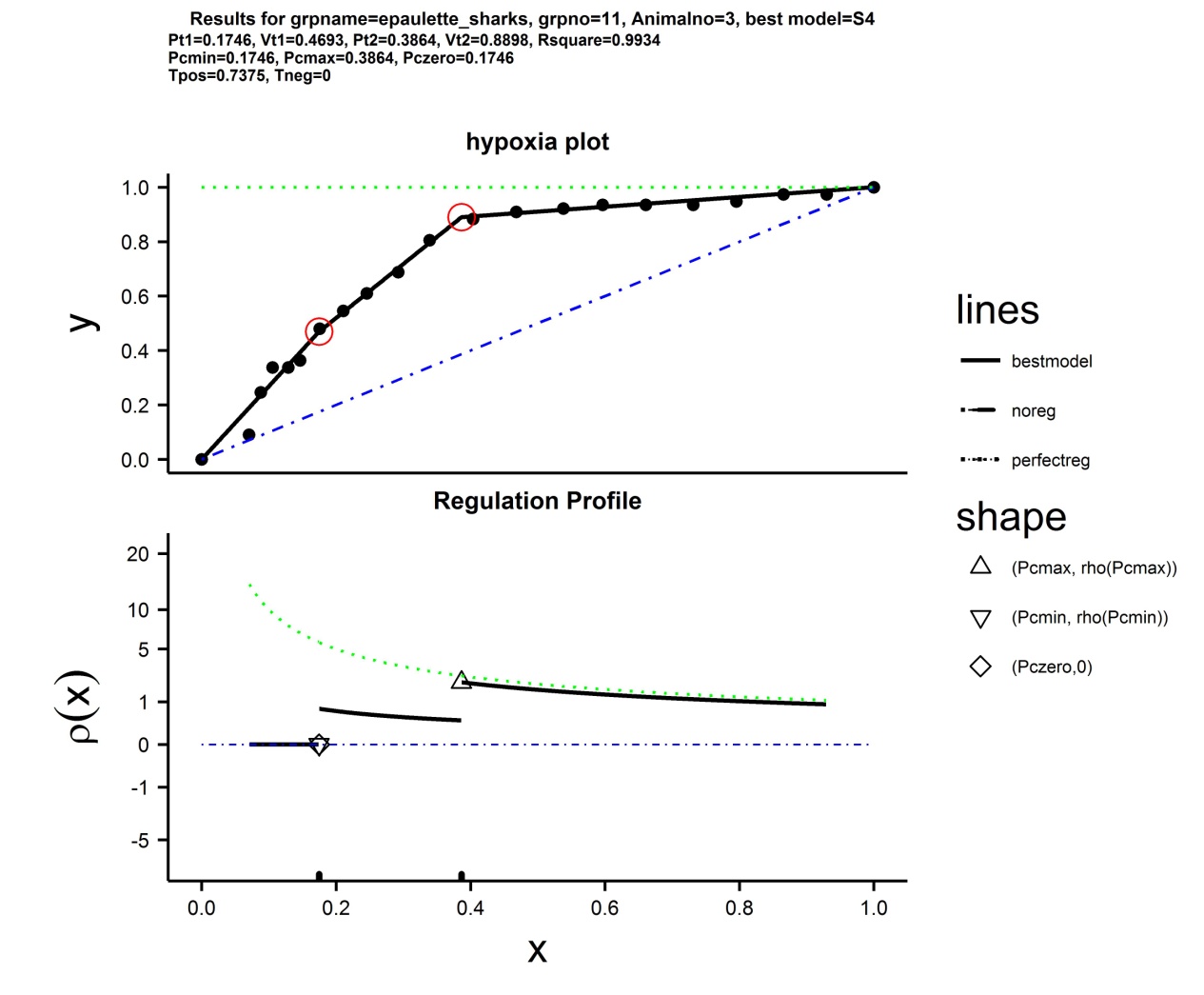

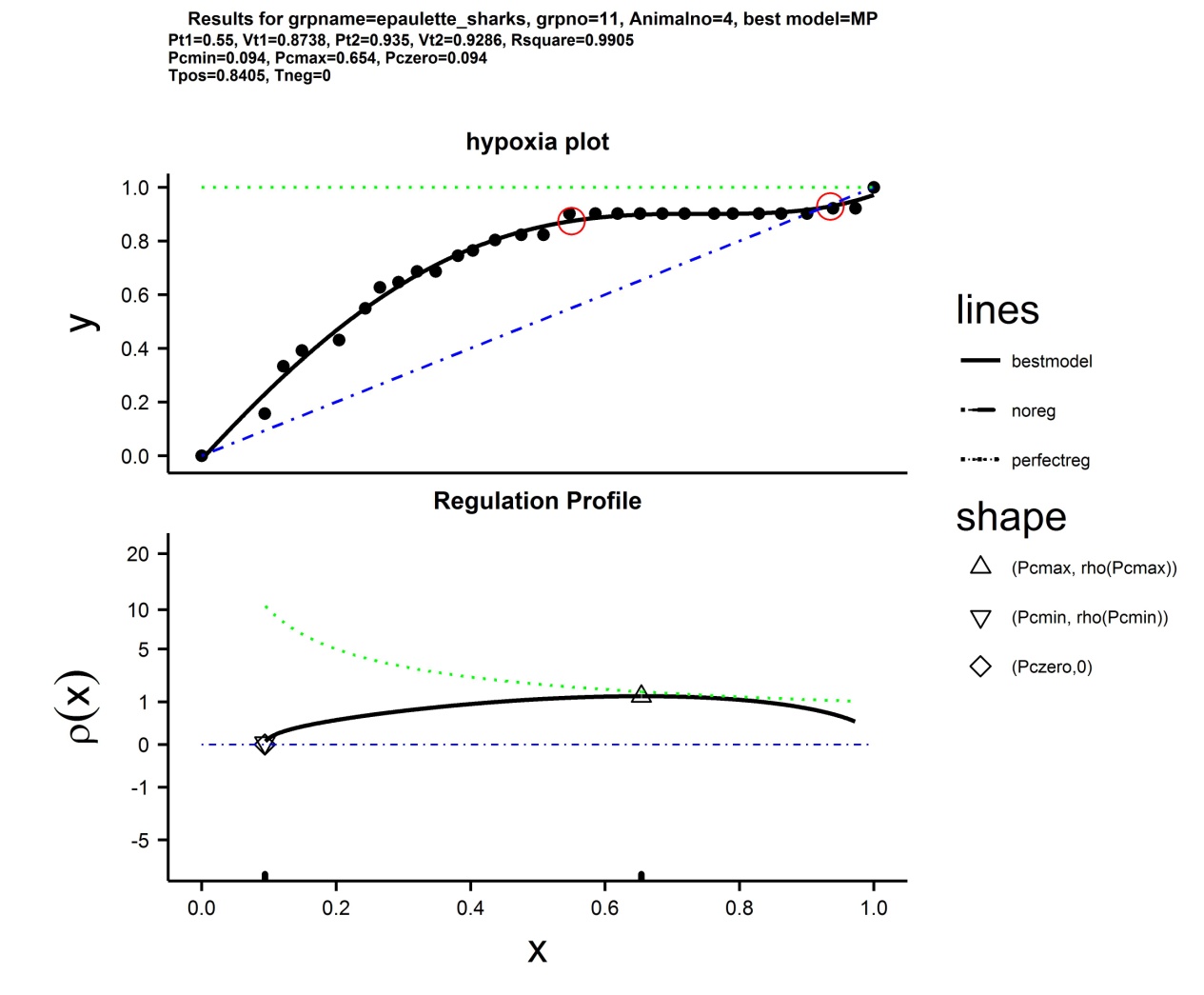

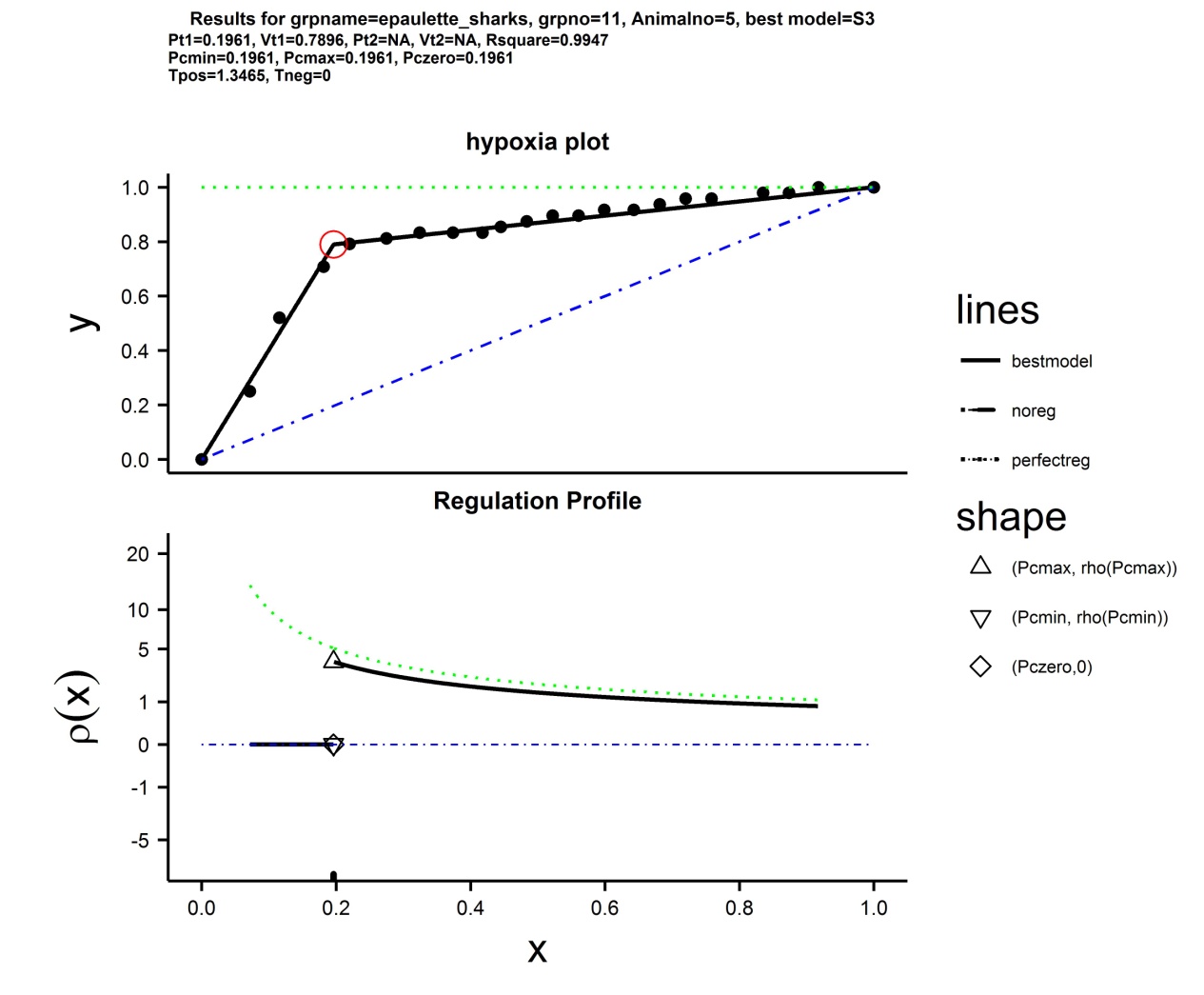

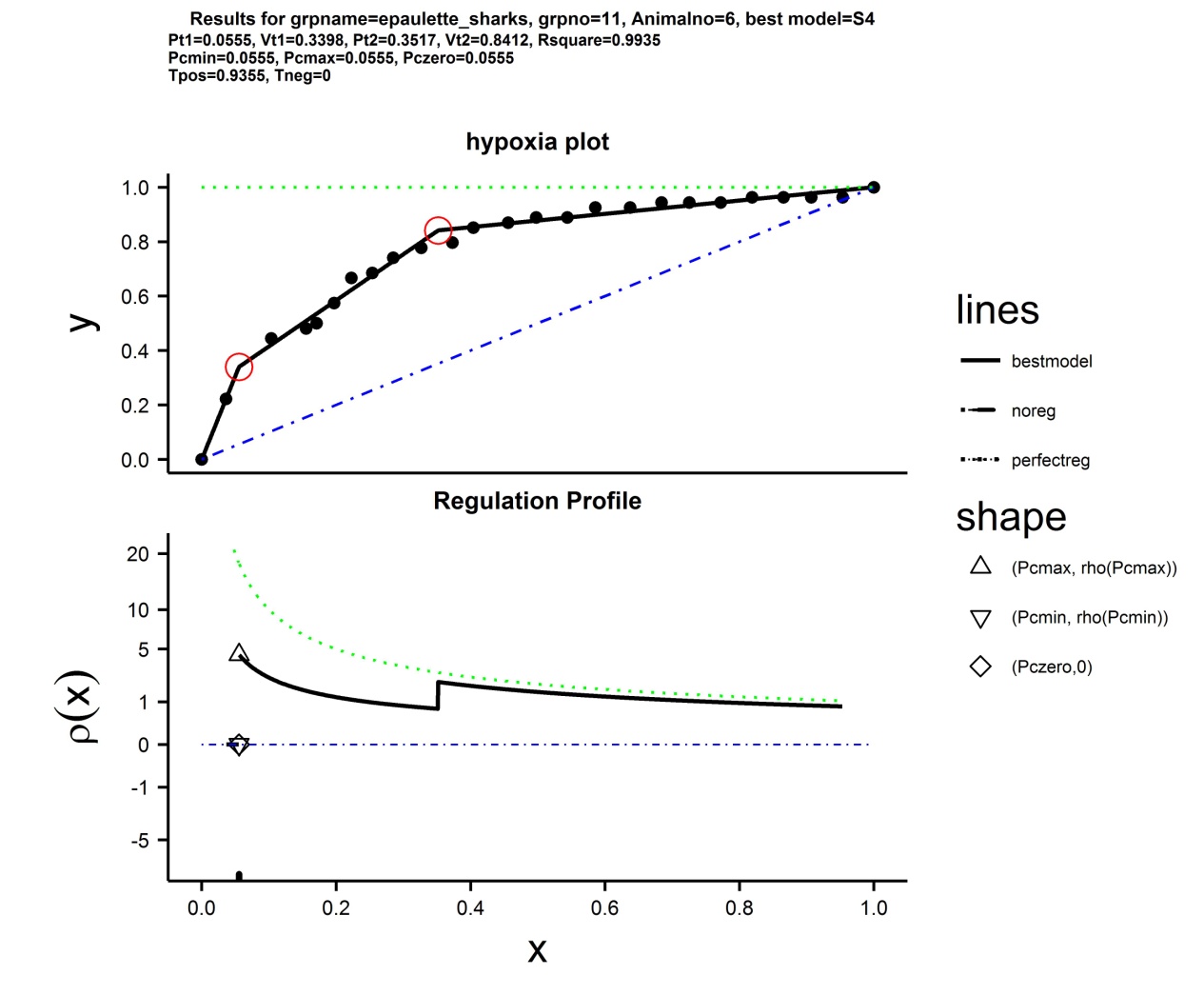

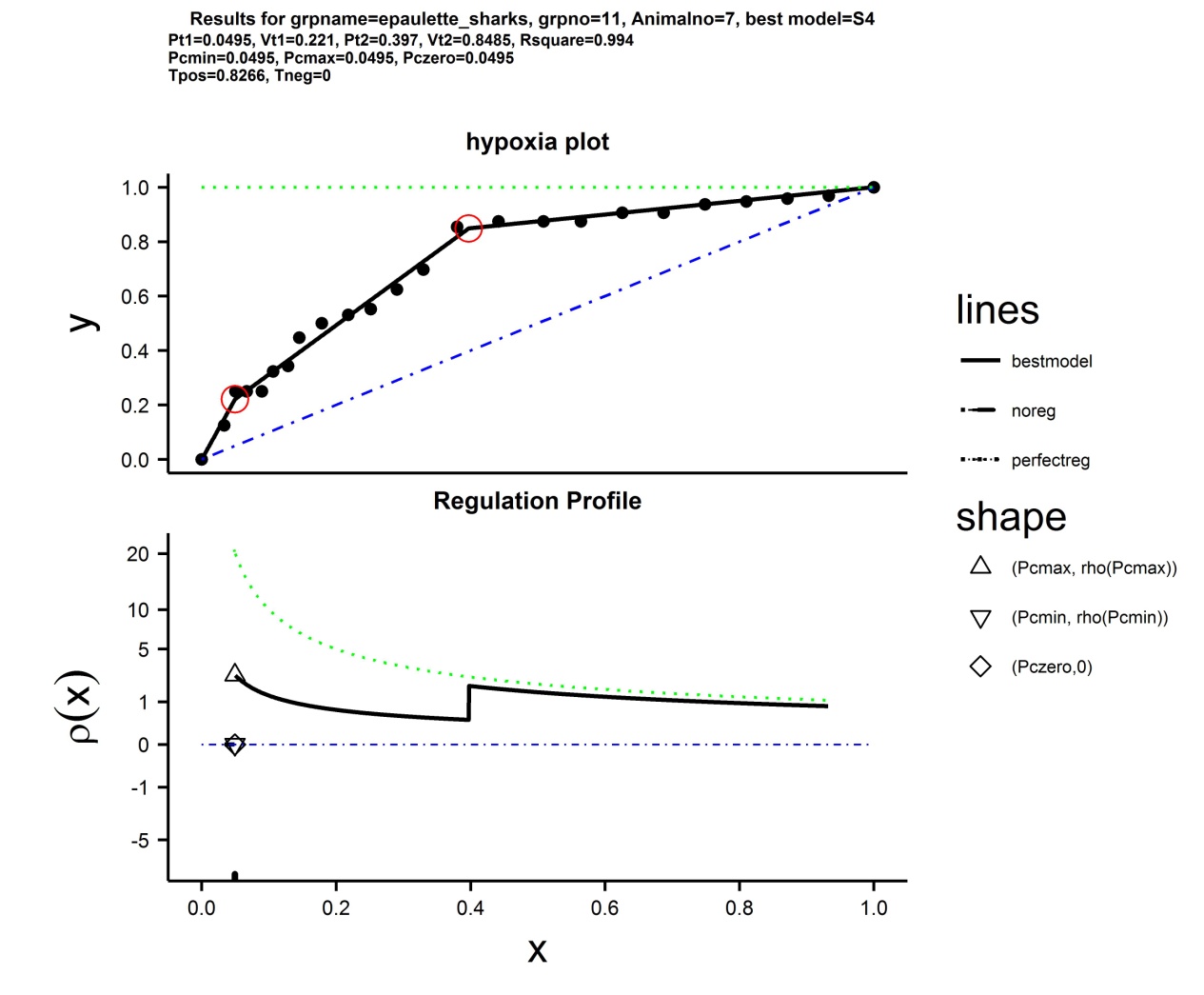

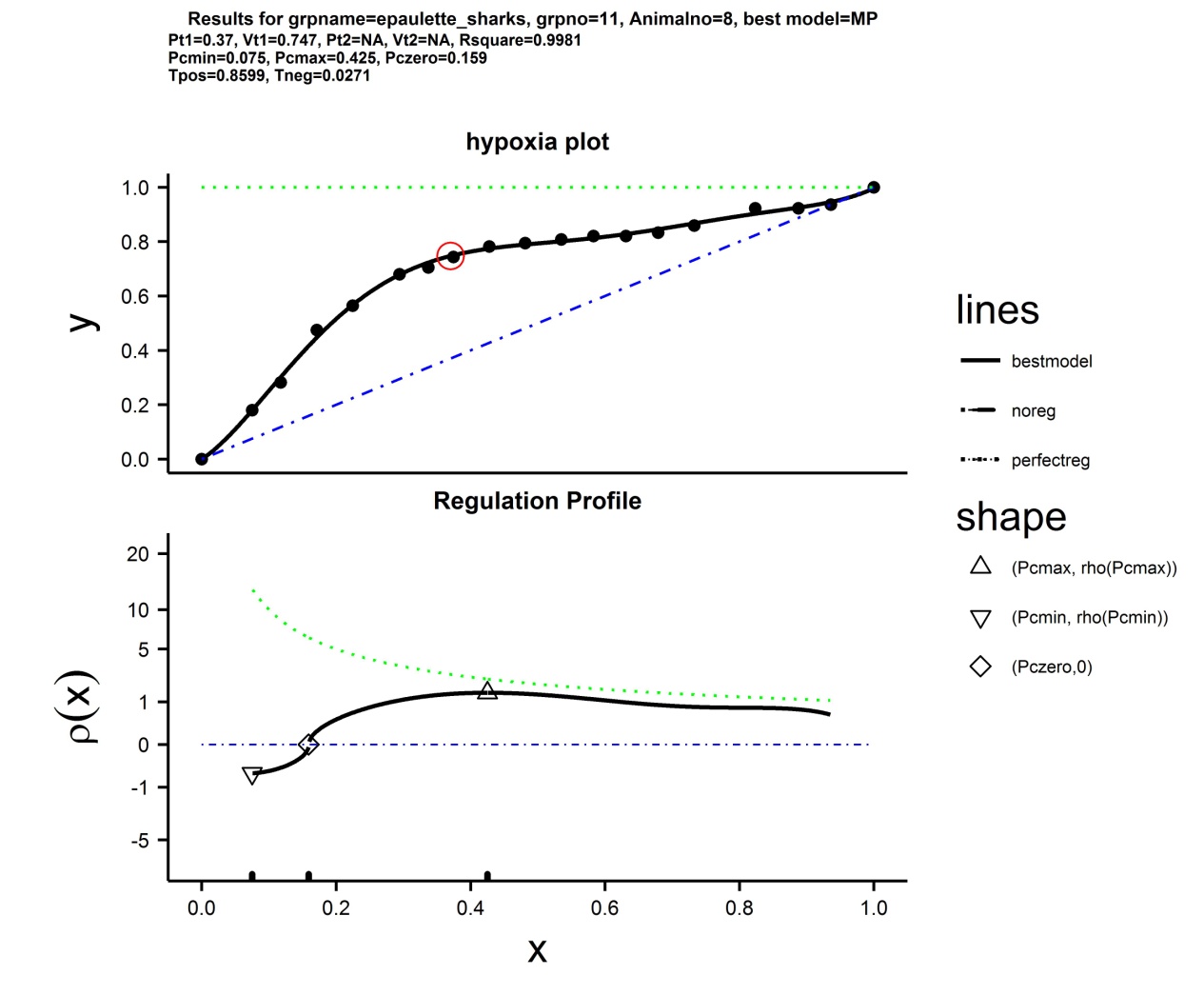

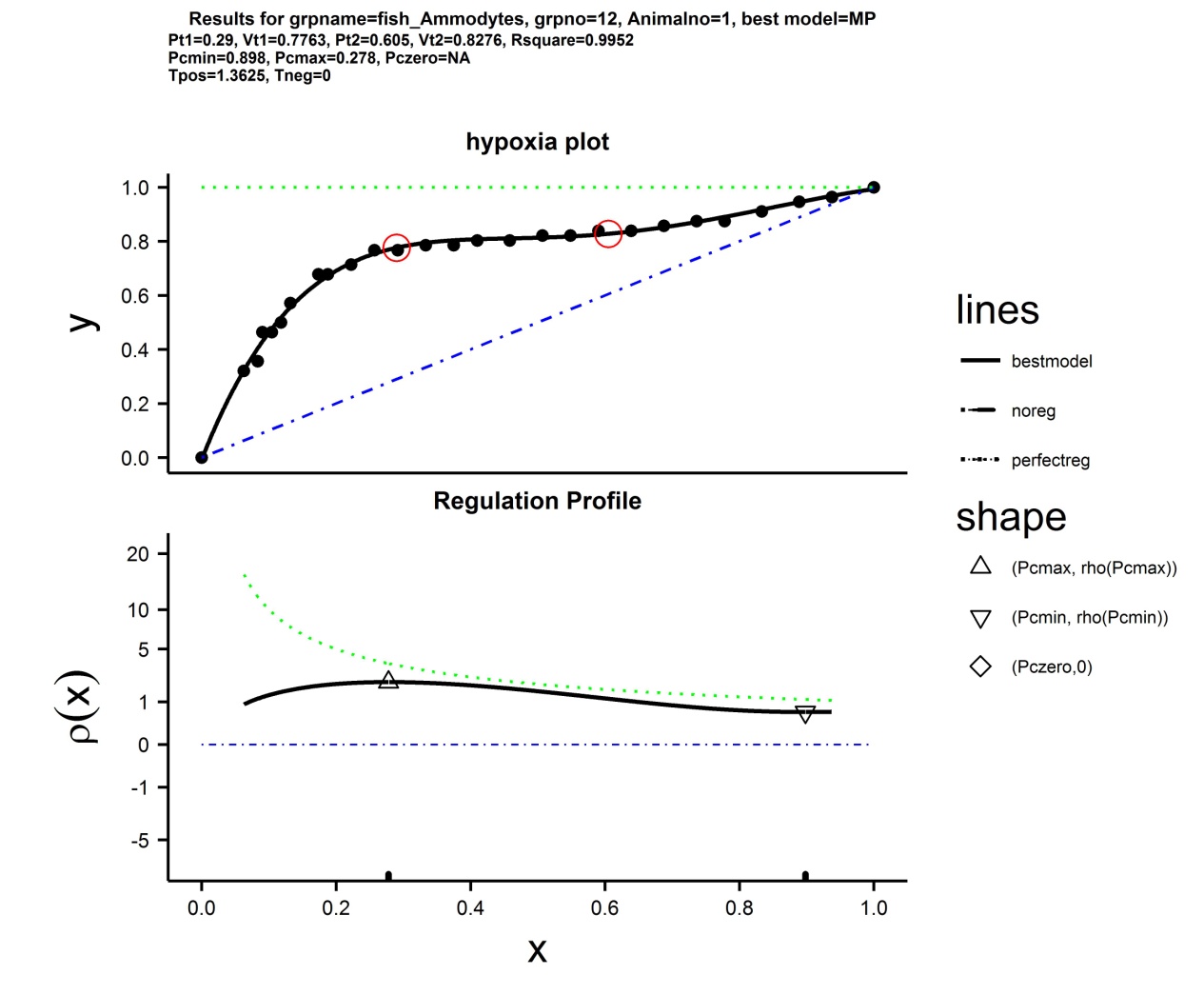

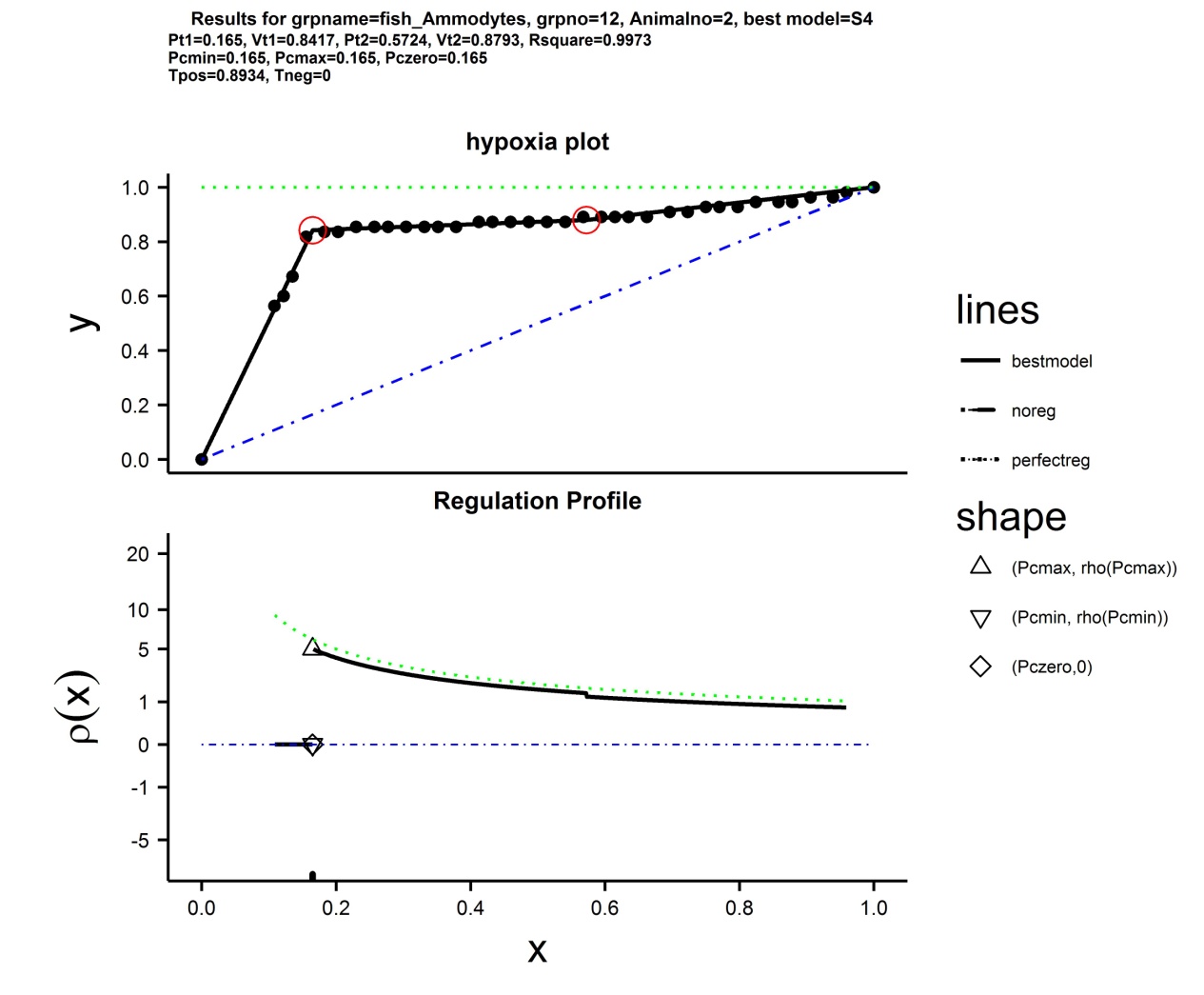

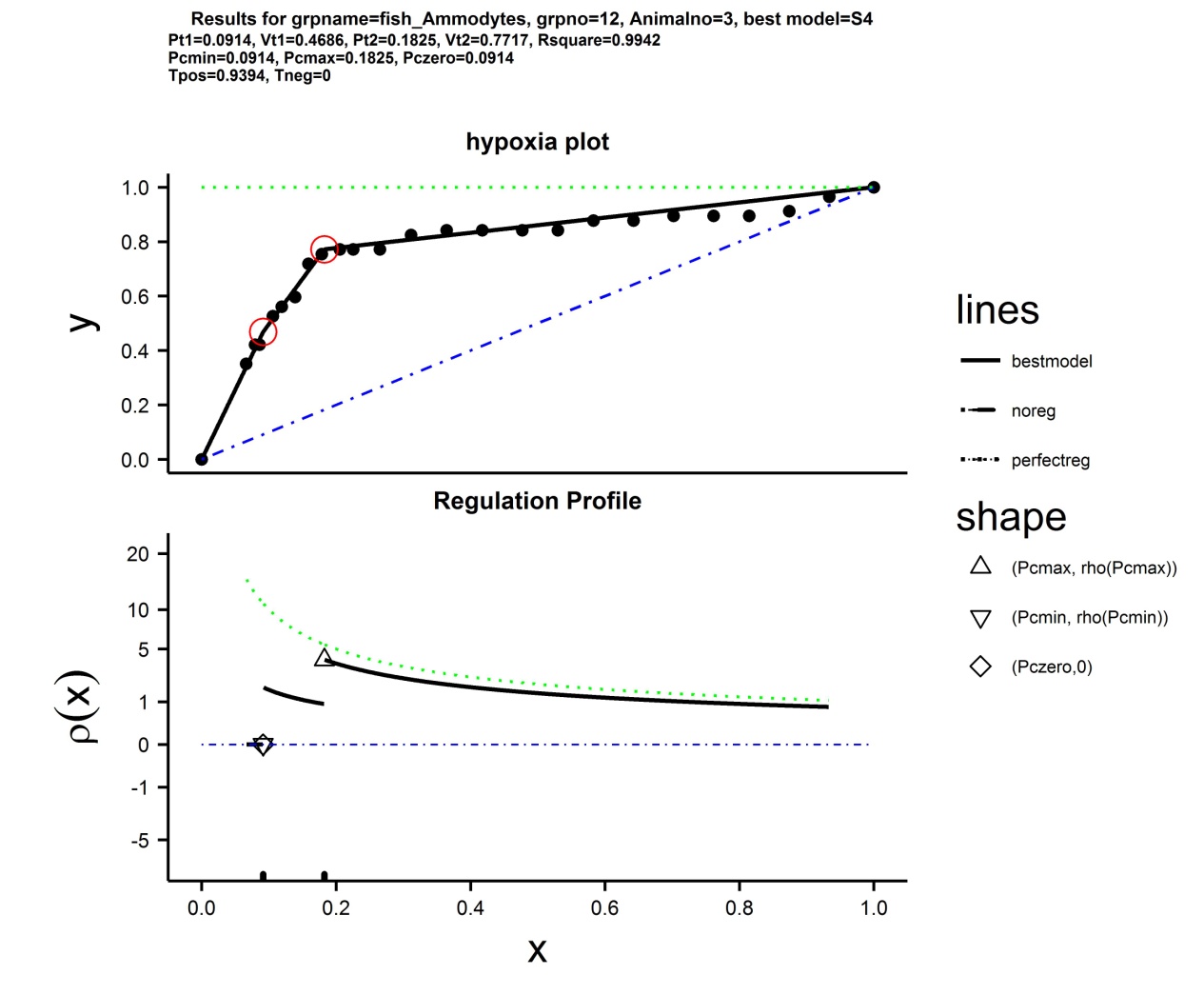

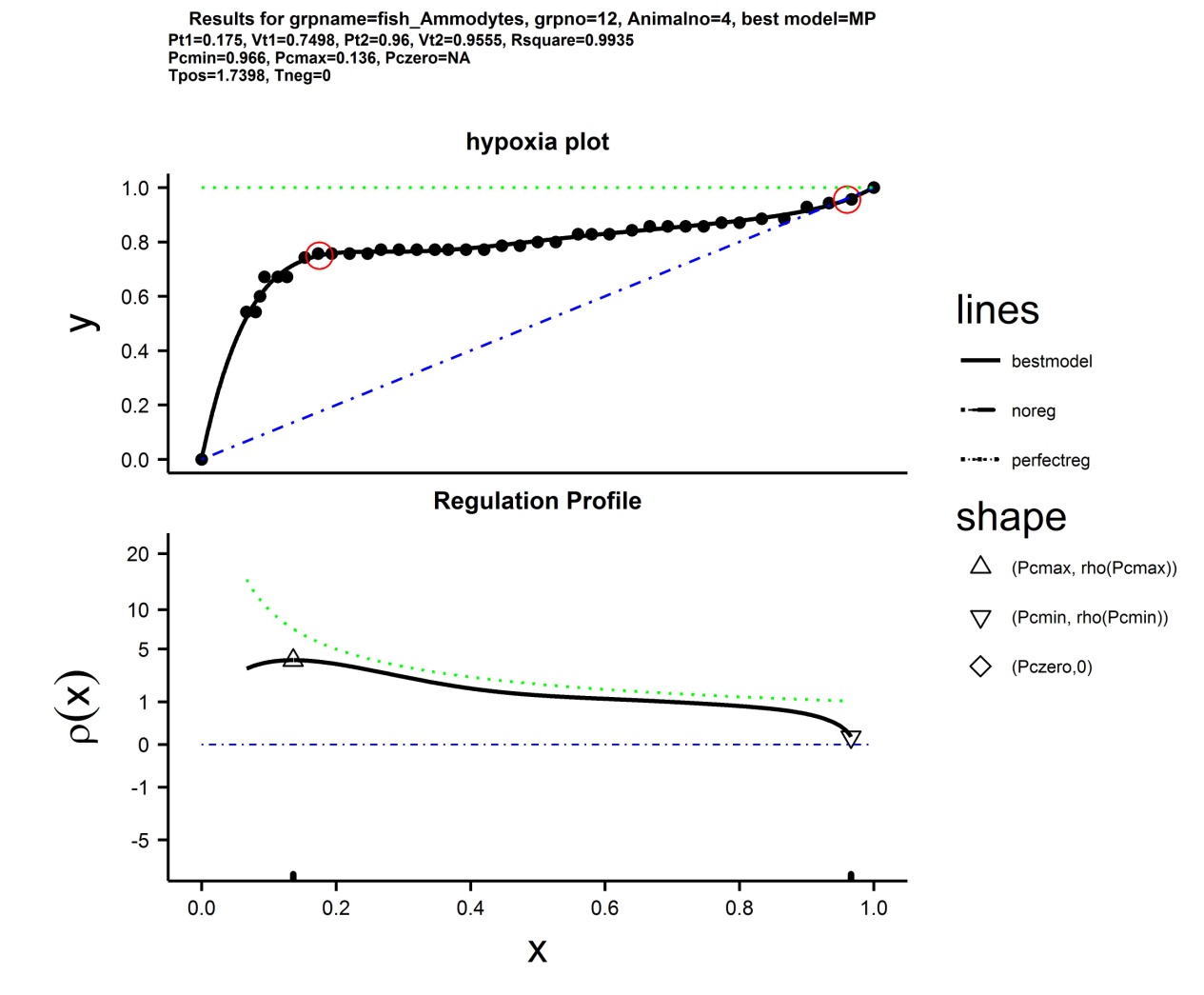

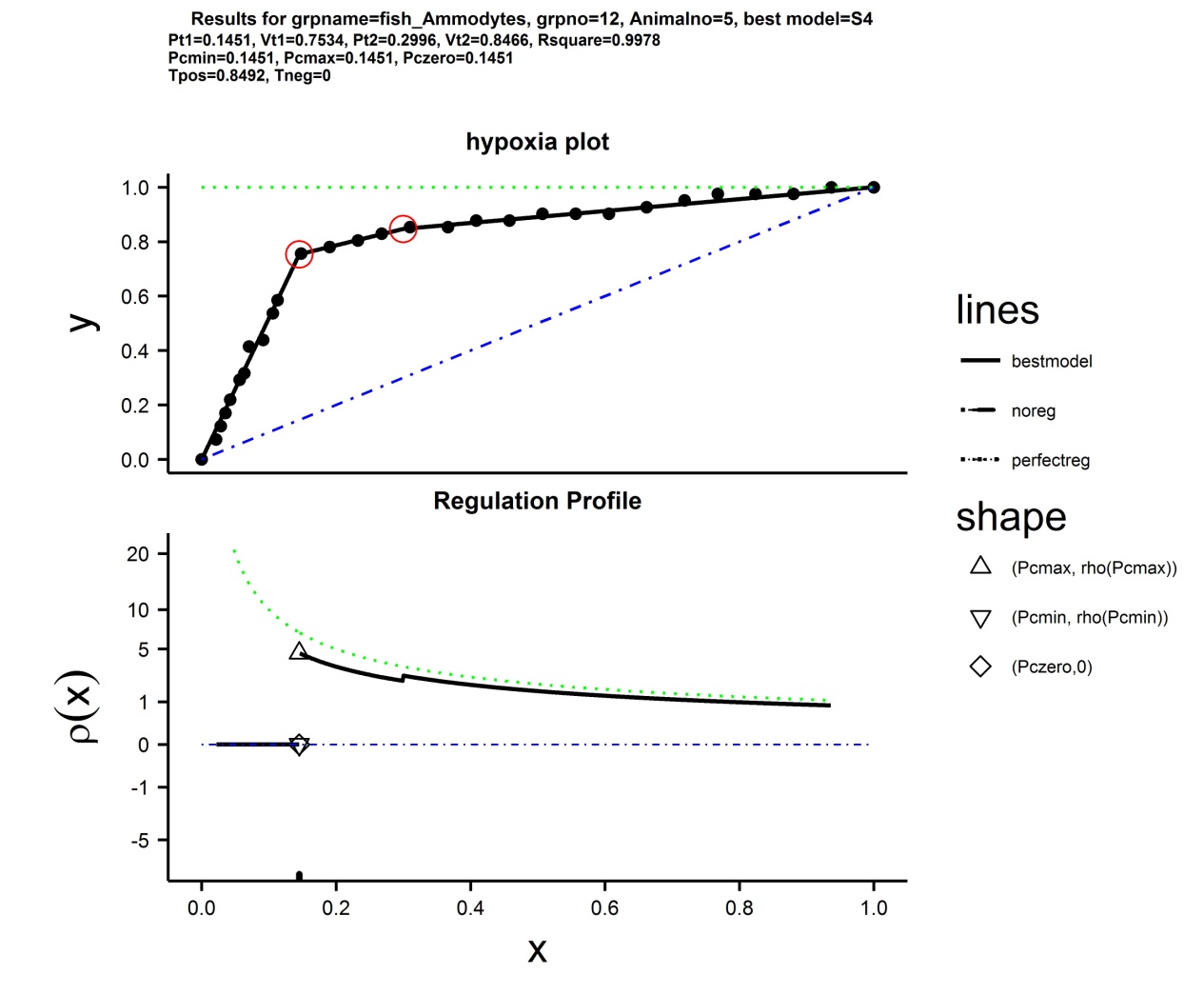

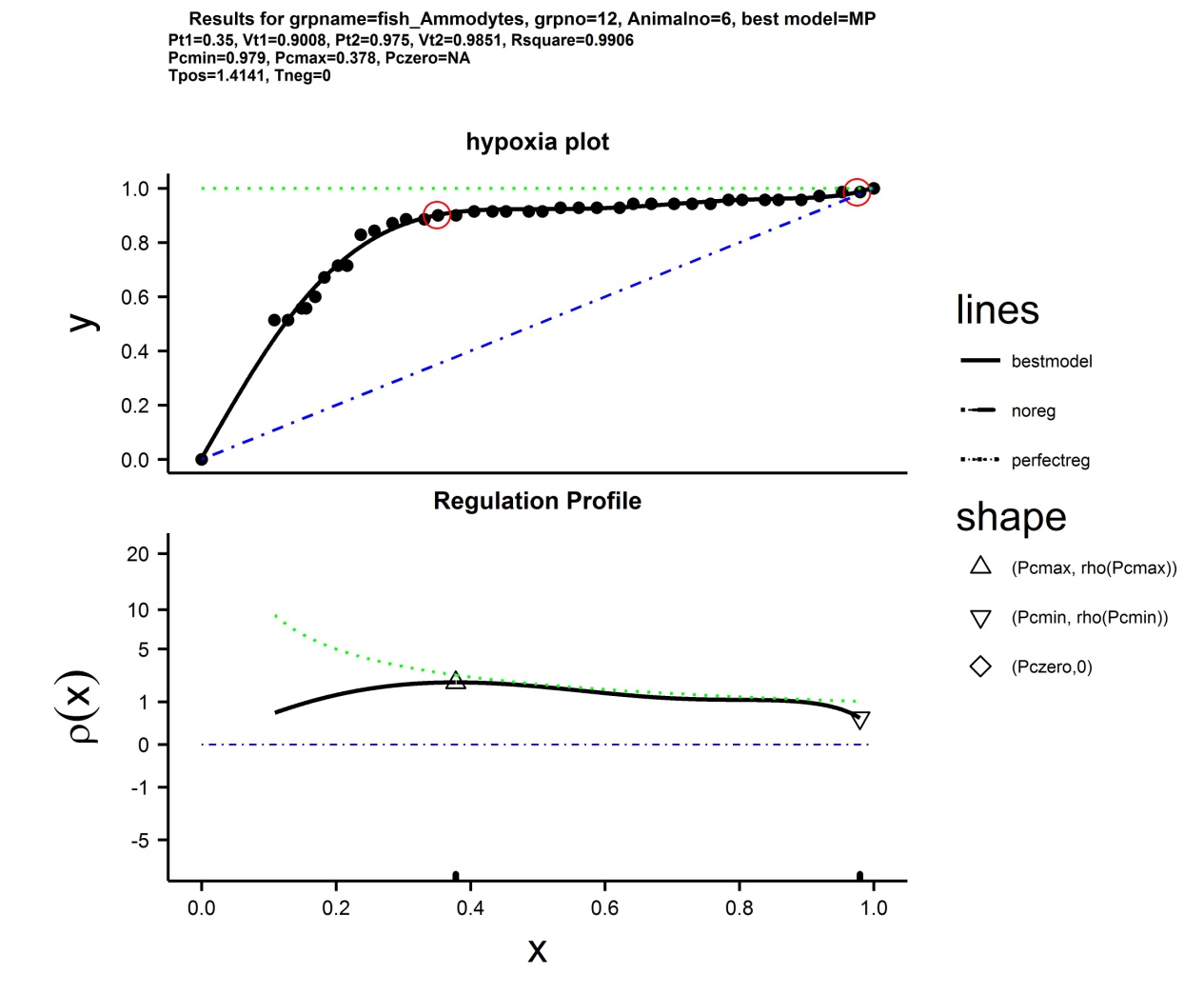

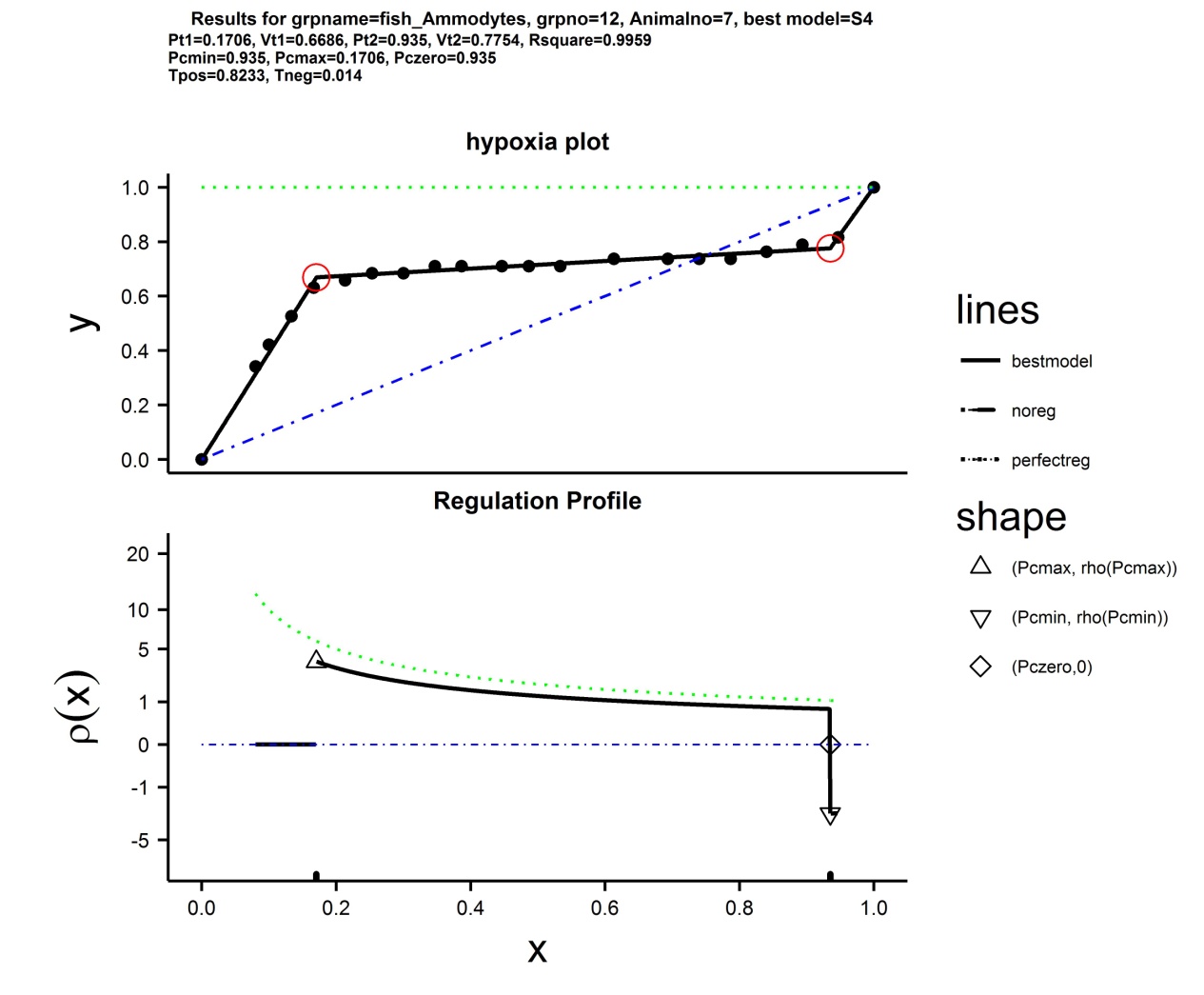

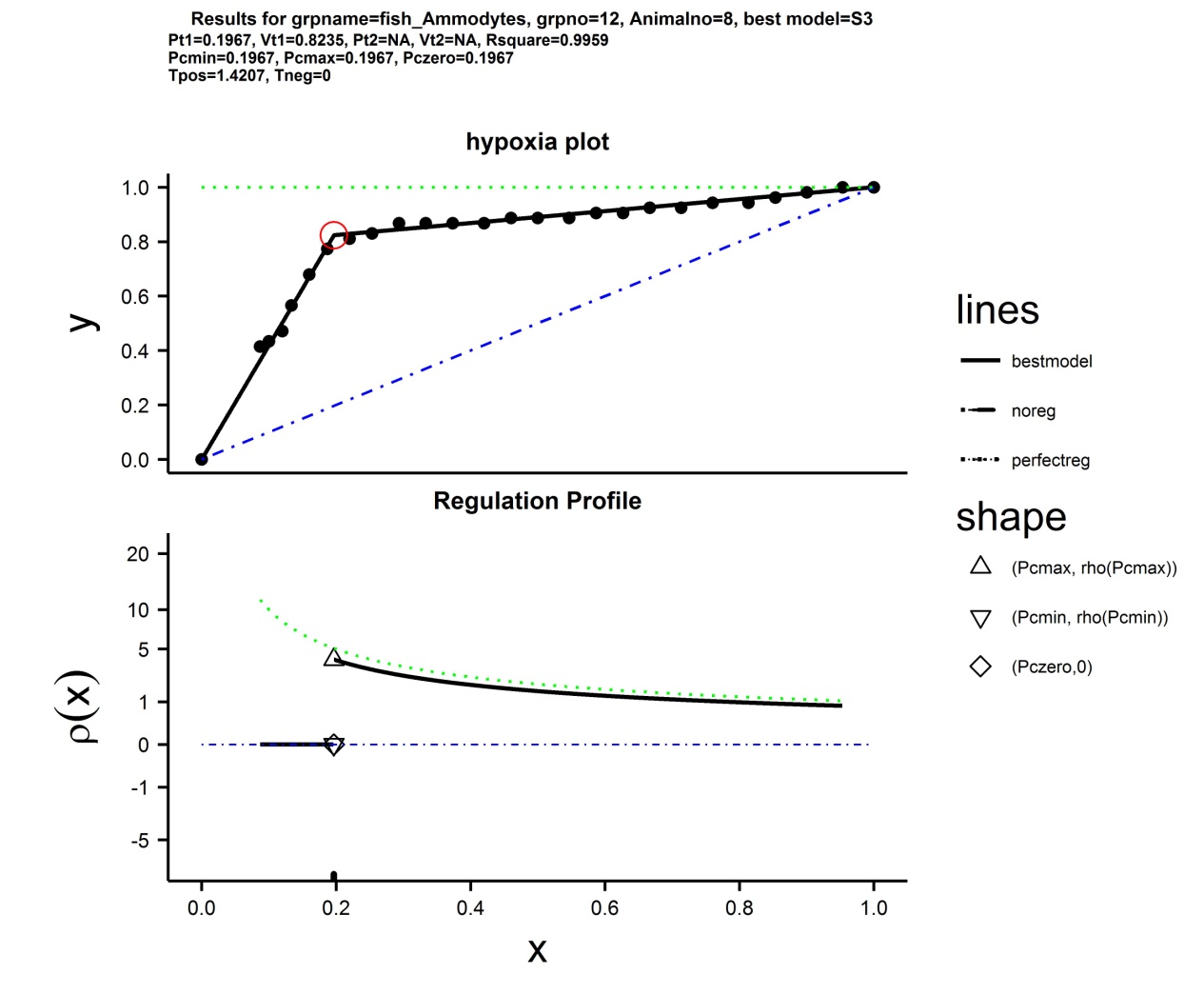

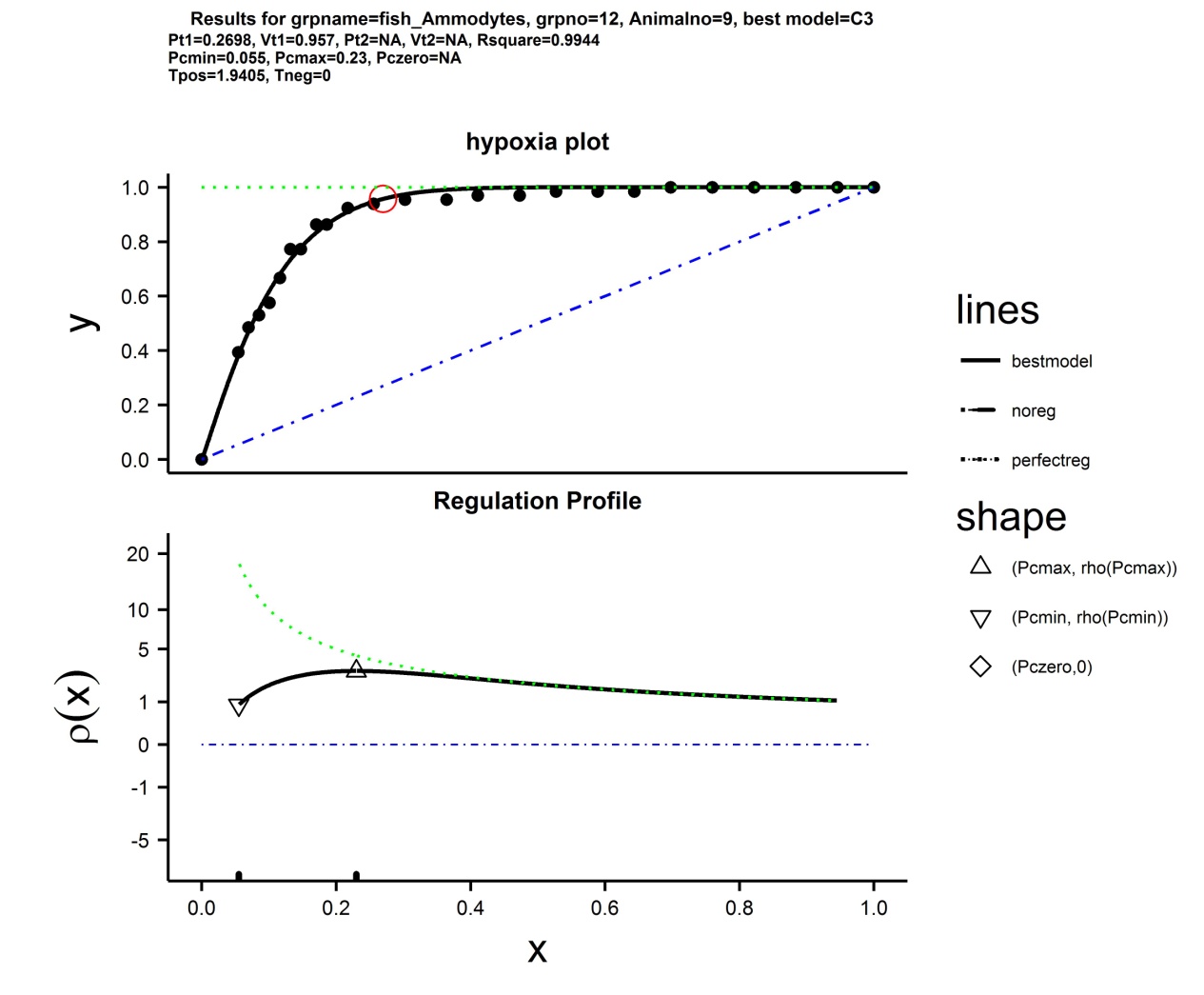

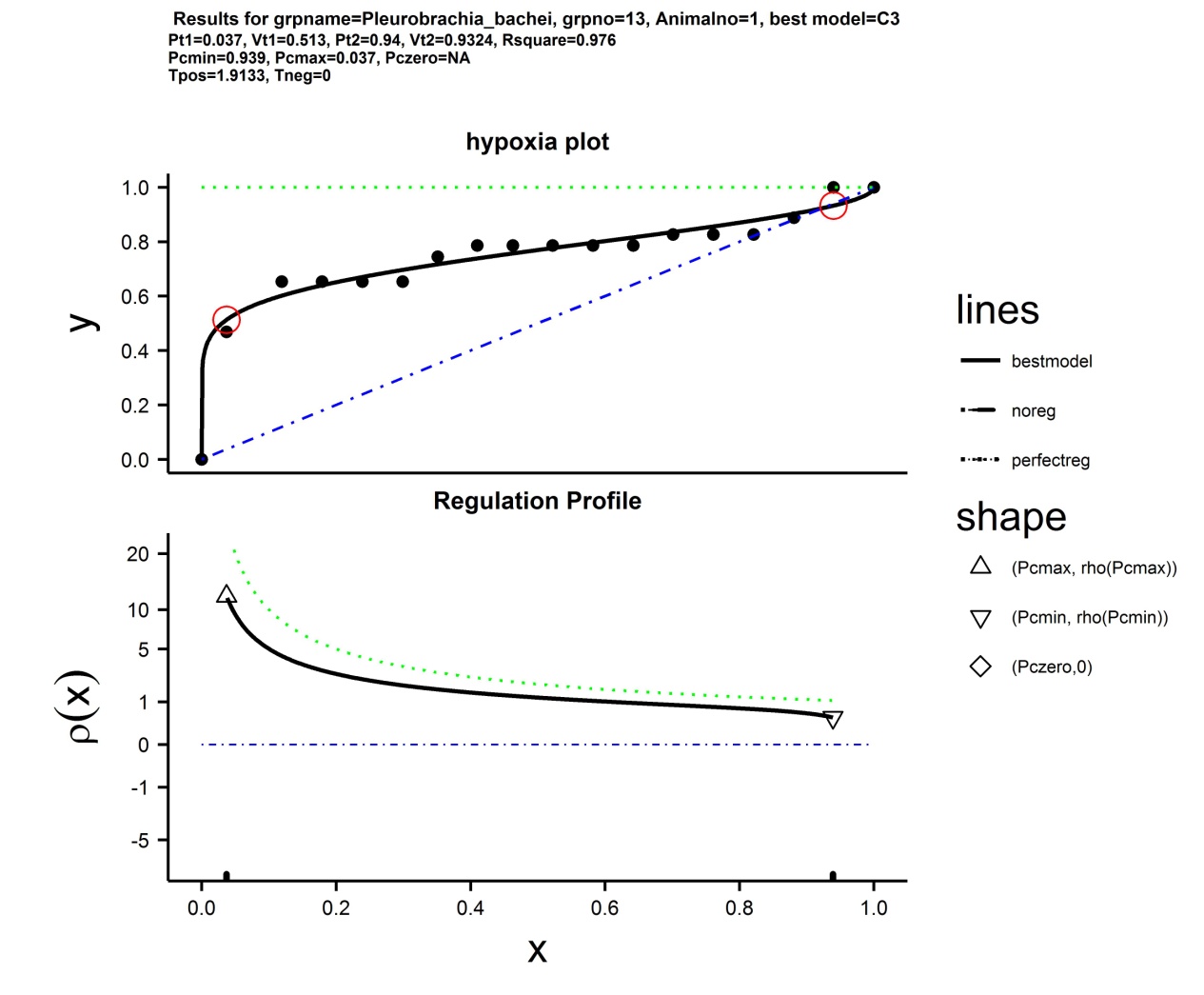

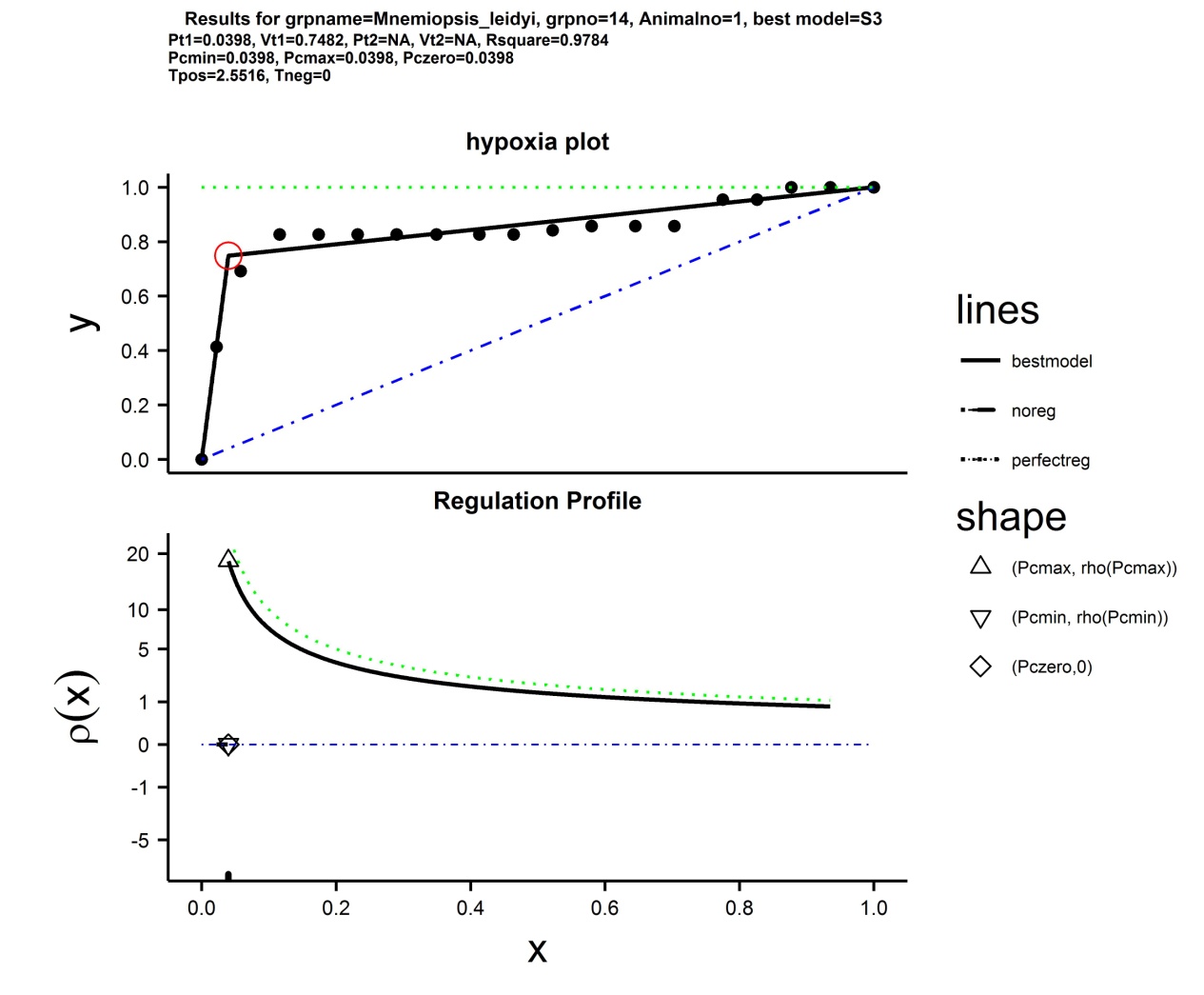

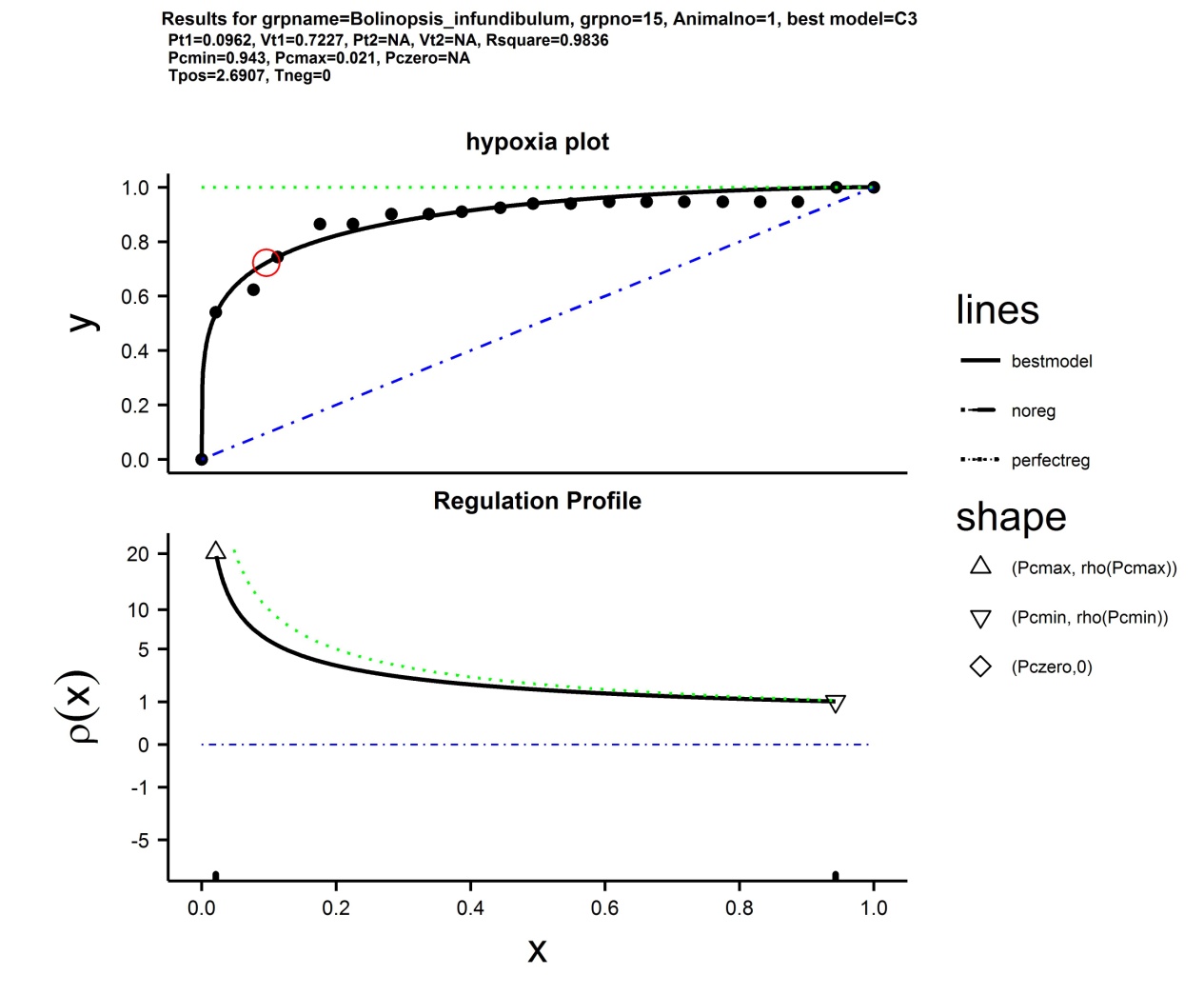

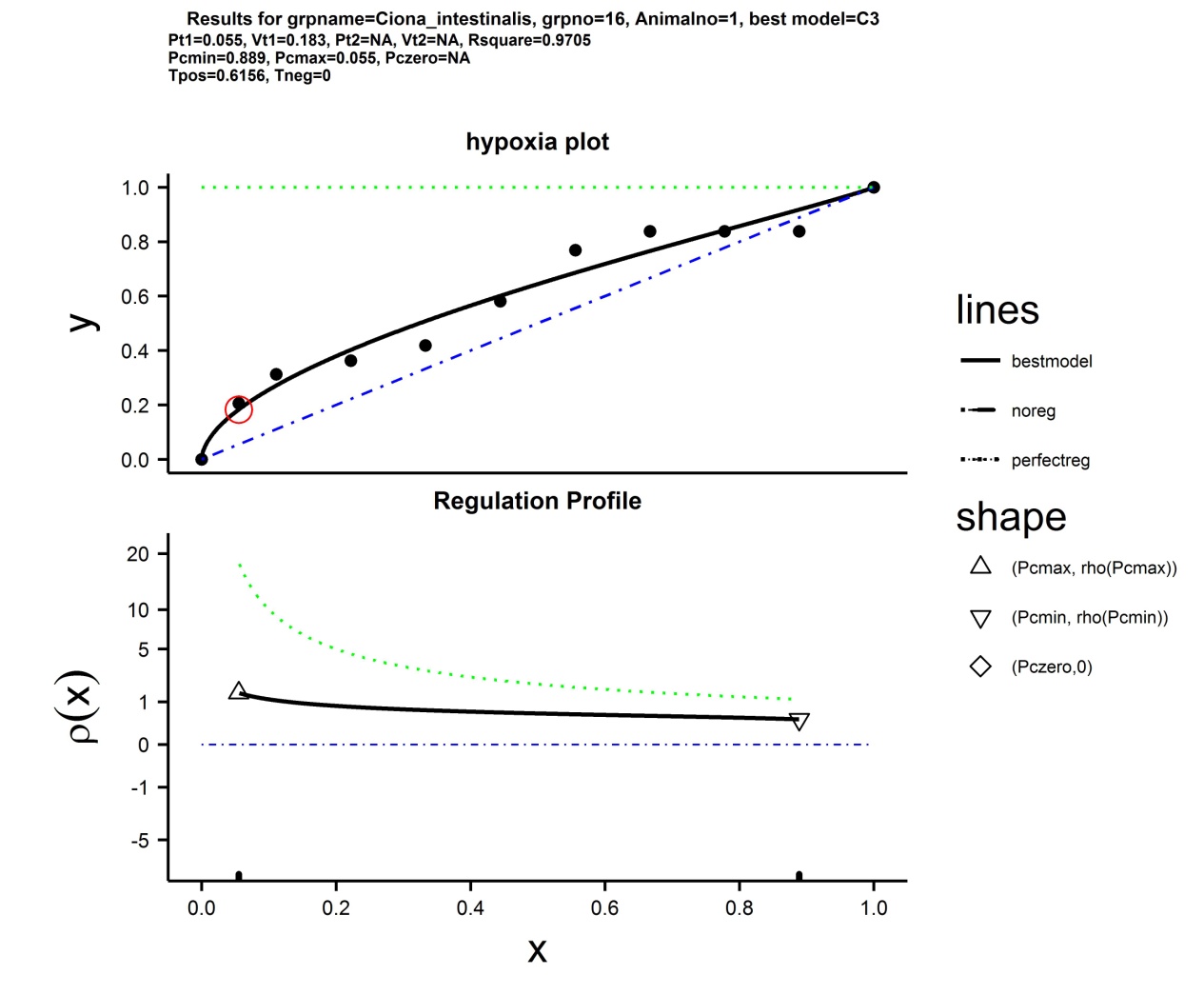

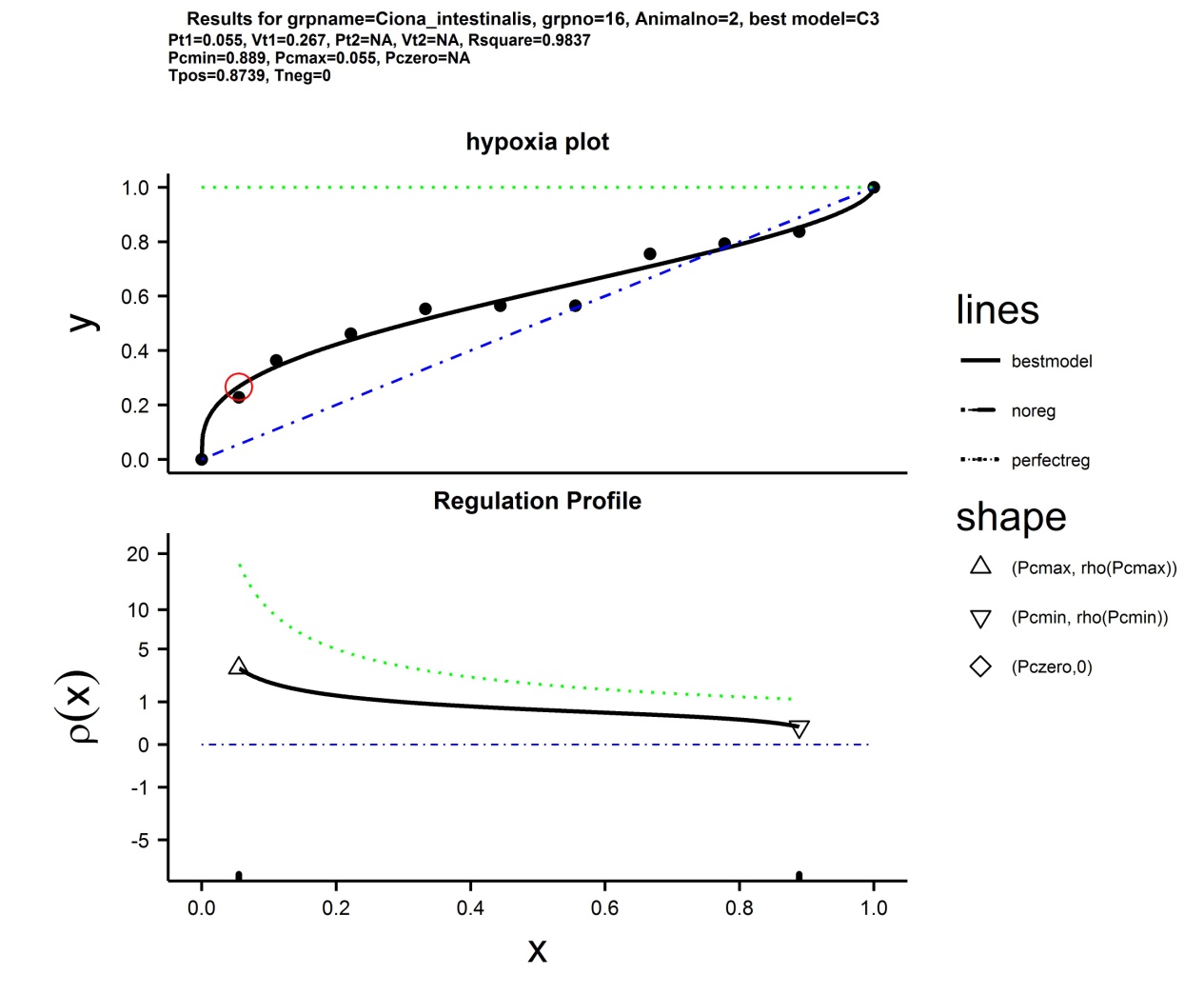

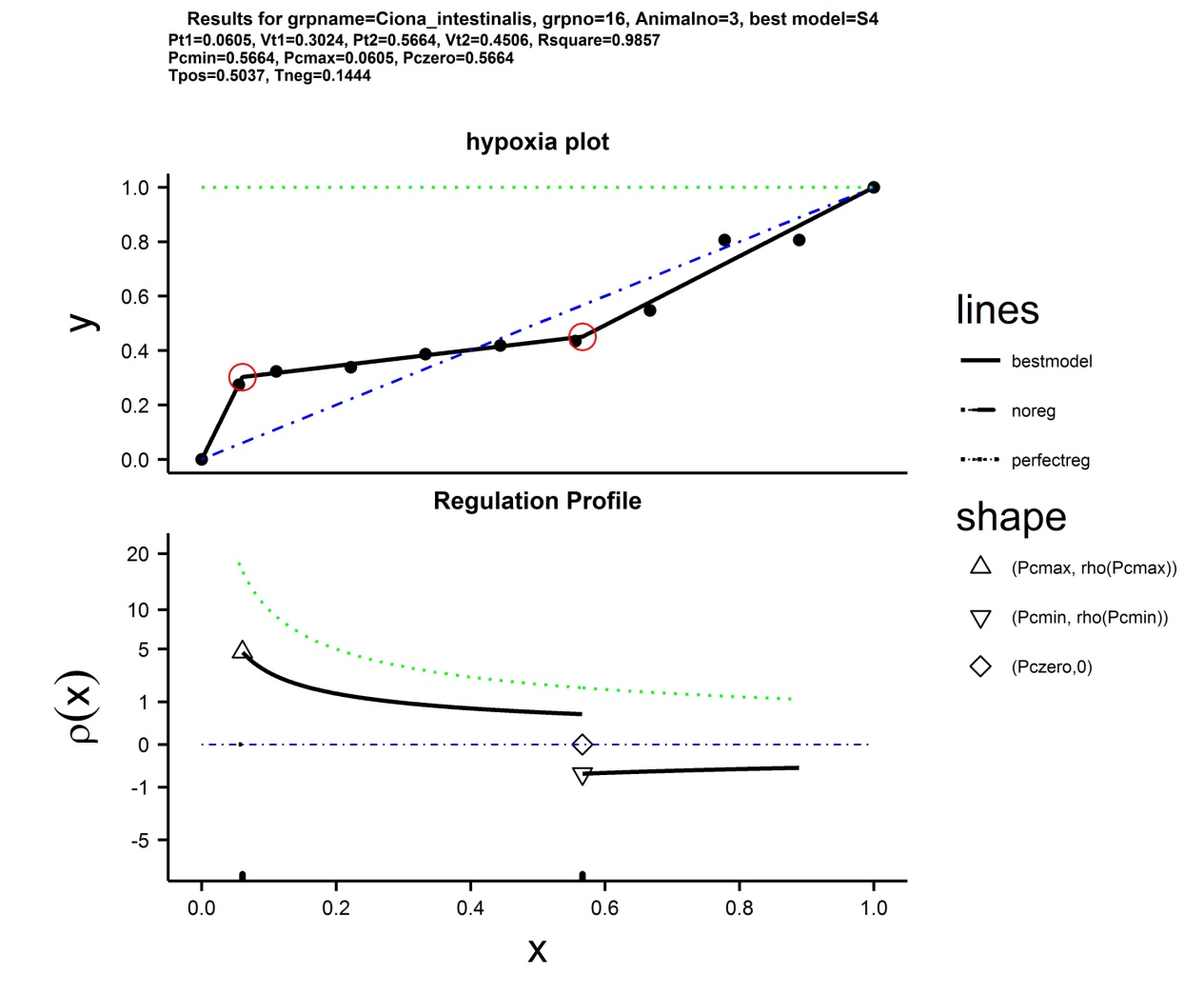

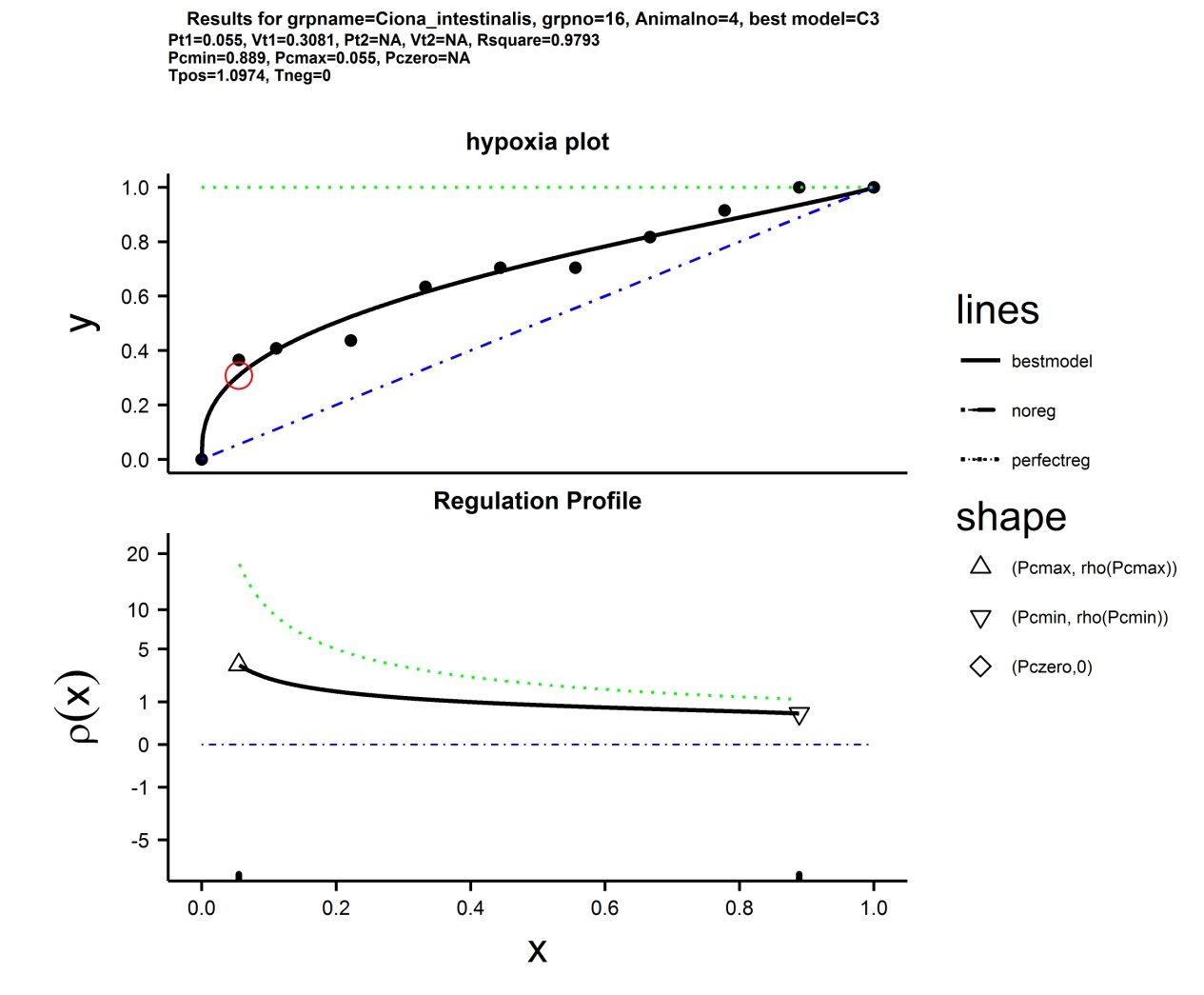

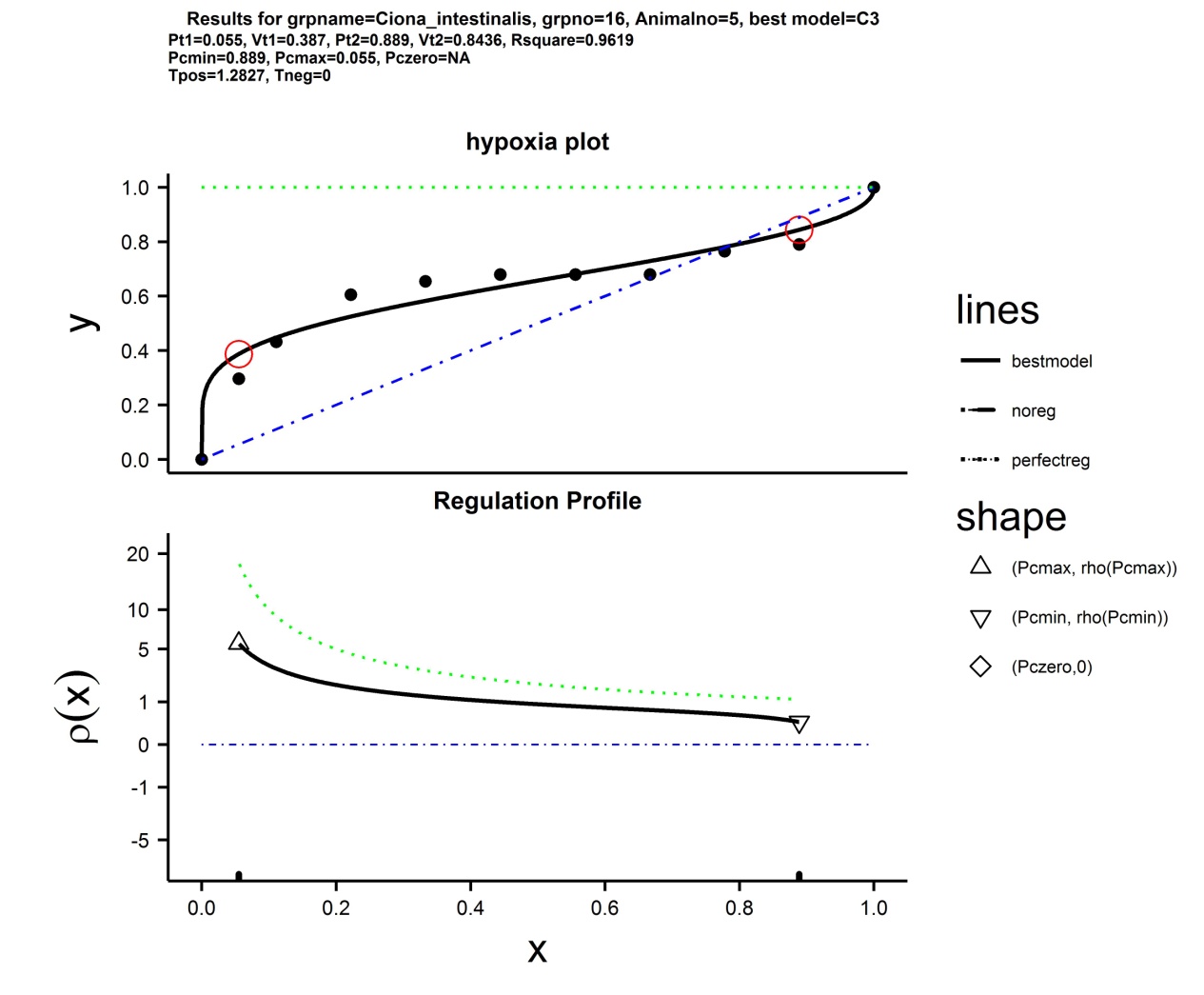

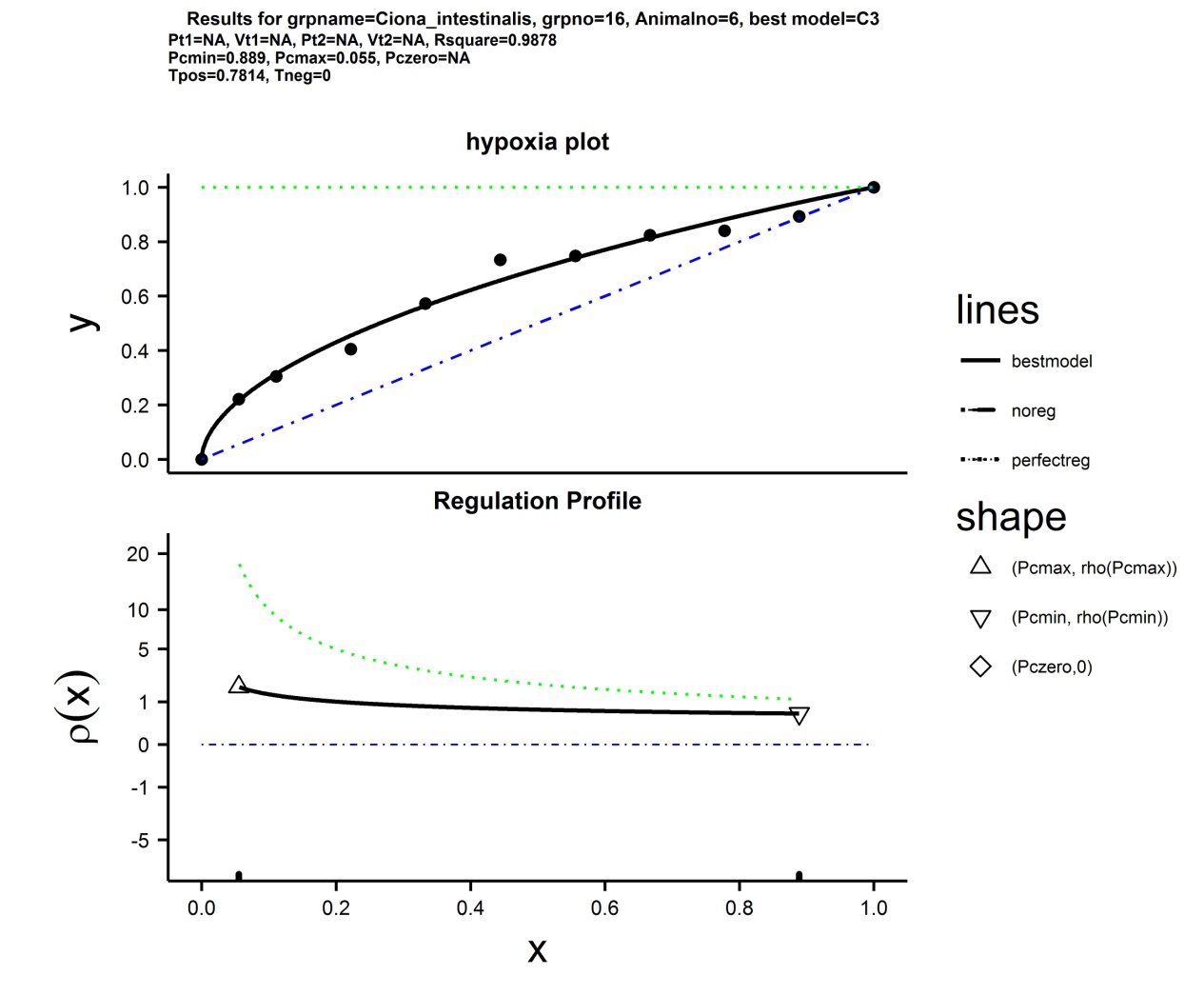

Supplement: S1 Results — (DOCX) [file pone.0208836.s002.docx]
