# Supplementary material for: Assessment of oxygen consumption in response to progressive hypoxia
Source: PLoS One. 2018 Dec 21;13(12):e0208836. doi: 10.1371/journal.pone.0208836 (PMC6303046; doi:10.1371/journal.pone.0208836)
Supplement: S1 Data — (DOC) [file pone.0208836.s003.doc]

groupname groupno animal relPO2 relVO2

1 zebra_5deg 1 12 1.00000000 1.00000000

2 zebra_5deg 1 12 0.95000000 1.00000000

3 zebra_5deg 1 12 0.90000000 1.00000000

4 zebra_5deg 1 12 0.85000000 1.00000000

5 zebra_5deg 1 12 0.80000000 1.00000000

6 zebra_5deg 1 12 0.75000000 0.85454546

7 zebra_5deg 1 12 0.70000000 0.85454546

8 zebra_5deg 1 12 0.65000000 0.85454546

9 zebra_5deg 1 12 0.60000000 0.85454546

10 zebra_5deg 1 12 0.55000000 0.70909091

11 zebra_5deg 1 12 0.50000000 0.69090909

12 zebra_5deg 1 12 0.45000000 0.69090909

13 zebra_5deg 1 12 0.40000000 0.69090909

14 zebra_5deg 1 12 0.35000000 0.63636364

15 zebra_5deg 1 12 0.30000000 0.63636364

16 zebra_5deg 1 12 0.25000000 0.63636364

17 zebra_5deg 1 12 0.20000000 0.52727273

18 zebra_5deg 1 12 0.15000000 0.52727273

19 zebra_5deg 1 12 0.10000000 0.50909091

20 zebra_5deg 1 12 0.05000000 0.29090909

21 zebra_5deg 1 12 0.00000000 0.00000000

22 zebra_5deg 1 13 1.00000000 1.00000000

23 zebra_5deg 1 13 0.95000000 1.00000000

24 zebra_5deg 1 13 0.90000000 0.88095238

25 zebra_5deg 1 13 0.85000000 0.73809524

26 zebra_5deg 1 13 0.80000000 0.73809524

27 zebra_5deg 1 13 0.75000000 0.73809524

28 zebra_5deg 1 13 0.70000000 0.73809524

29 zebra_5deg 1 13 0.65000000 0.73809524

30 zebra_5deg 1 13 0.60000000 0.73809524

31 zebra_5deg 1 13 0.55000000 0.64285714

32 zebra_5deg 1 13 0.50000000 0.61904762

33 zebra_5deg 1 13 0.45000000 0.61904762

34 zebra_5deg 1 13 0.40000000 0.61904762

35 zebra_5deg 1 13 0.35000000 0.61904762

36 zebra_5deg 1 13 0.30000000 0.57142857

37 zebra_5deg 1 13 0.25000000 0.57142857

38 zebra_5deg 1 13 0.20000000 0.57142857

39 zebra_5deg 1 13 0.15000000 0.50000000

40 zebra_5deg 1 13 0.10000000 0.50000000

41 zebra_5deg 1 13 0.05000000 0.42857143

42 zebra_5deg 1 13 0.00000000 0.00000000

43 zebra_5deg 1 14 1.00000000 1.00000000

44 zebra_5deg 1 14 0.95000000 1.00000000

45 zebra_5deg 1 14 0.90000000 1.00000000

46 zebra_5deg 1 14 0.85000000 1.00000000

47 zebra_5deg 1 14 0.80000000 1.00000000

48 zebra_5deg 1 14 0.75000000 0.80434783

49 zebra_5deg 1 14 0.70000000 0.80434783

50 zebra_5deg 1 14 0.65000000 0.80434783

51 zebra_5deg 1 14 0.60000000 0.80434783

52 zebra_5deg 1 14 0.55000000 0.73913043

53 zebra_5deg 1 14 0.50000000 0.73913043

54 zebra_5deg 1 14 0.45000000 0.73913043

55 zebra_5deg 1 14 0.40000000 0.73913043

56 zebra_5deg 1 14 0.35000000 0.73913043

57 zebra_5deg 1 14 0.30000000 0.73913043

58 zebra_5deg 1 14 0.25000000 0.63043478

59 zebra_5deg 1 14 0.20000000 0.63043478

60 zebra_5deg 1 14 0.15000000 0.56521739

61 zebra_5deg 1 14 0.10000000 0.56521739

62 zebra_5deg 1 14 0.05000000 0.34782609

63 zebra_5deg 1 14 0.00000000 0.00000000

64 zebra_5deg 1 15 1.00000000 1.00000000

65 zebra_5deg 1 15 0.95000000 1.00000000

66 zebra_5deg 1 15 0.90000000 1.00000000

67 zebra_5deg 1 15 0.85000000 1.00000000

68 zebra_5deg 1 15 0.80000000 1.00000000

69 zebra_5deg 1 15 0.75000000 1.00000000

70 zebra_5deg 1 15 0.70000000 1.00000000

71 zebra_5deg 1 15 0.65000000 0.79487180

72 zebra_5deg 1 15 0.60000000 0.87179487

73 zebra_5deg 1 15 0.55000000 0.84615385

74 zebra_5deg 1 15 0.50000000 0.79487180

75 zebra_5deg 1 15 0.45000000 0.79487180

76 zebra_5deg 1 15 0.40000000 0.79487180

77 zebra_5deg 1 15 0.35000000 0.82051282

78 zebra_5deg 1 15 0.30000000 0.66666667

79 zebra_5deg 1 15 0.25000000 0.66666667

80 zebra_5deg 1 15 0.20000000 0.71794872

81 zebra_5deg 1 15 0.15000000 0.66666667

82 zebra_5deg 1 15 0.10000000 0.66666667

83 zebra_5deg 1 15 0.05000000 0.43589744

84 zebra_5deg 1 15 0.00000000 0.00000000

85 zebra_5deg 1 16 1.00000000 1.00000000

86 zebra_5deg 1 16 0.95000000 1.00000000

87 zebra_5deg 1 16 0.90000000 1.00000000

88 zebra_5deg 1 16 0.85000000 1.00000000

89 zebra_5deg 1 16 0.80000000 1.00000000

90 zebra_5deg 1 16 0.75000000 1.00000000

91 zebra_5deg 1 16 0.70000000 0.86440678

92 zebra_5deg 1 16 0.65000000 0.79661017

93 zebra_5deg 1 16 0.60000000 0.79661017

94 zebra_5deg 1 16 0.55000000 0.79661017

95 zebra_5deg 1 16 0.50000000 0.64406780

96 zebra_5deg 1 16 0.45000000 0.64406780

97 zebra_5deg 1 16 0.40000000 0.64406780

98 zebra_5deg 1 16 0.35000000 0.64406780

99 zebra_5deg 1 16 0.30000000 0.55932203

100 zebra_5deg 1 16 0.25000000 0.55932203

101 zebra_5deg 1 16 0.20000000 0.55932203

102 zebra_5deg 1 16 0.15000000 0.42372881

103 zebra_5deg 1 16 0.10000000 0.42372881

104 zebra_5deg 1 16 0.05000000 0.42372881

105 zebra_5deg 1 16 0.00000000 0.00000000

106 zebra_5deg 1 17 1.00000000 1.00000000

107 zebra_5deg 1 17 0.95000000 1.00000000

108 zebra_5deg 1 17 0.90000000 1.00000000

109 zebra_5deg 1 17 0.85000000 0.74829932

110 zebra_5deg 1 17 0.80000000 0.74829932

111 zebra_5deg 1 17 0.75000000 0.60544218

112 zebra_5deg 1 17 0.70000000 0.60544218

113 zebra_5deg 1 17 0.65000000 0.60544218

114 zebra_5deg 1 17 0.60000000 0.60544218

115 zebra_5deg 1 17 0.55000000 0.60544218

116 zebra_5deg 1 17 0.50000000 0.50340136

117 zebra_5deg 1 17 0.45000000 0.50340136

118 zebra_5deg 1 17 0.40000000 0.45578231

119 zebra_5deg 1 17 0.35000000 0.45578231

120 zebra_5deg 1 17 0.30000000 0.38775510

121 zebra_5deg 1 17 0.25000000 0.38775510

122 zebra_5deg 1 17 0.20000000 0.38775510

123 zebra_5deg 1 17 0.15000000 0.29931973

124 zebra_5deg 1 17 0.10000000 0.29931973

125 zebra_5deg 1 17 0.05000000 0.25850340

126 zebra_5deg 1 17 0.00000000 0.00000000

127 zebra_15deg 2 6 1.00000000 1.00000000

128 zebra_15deg 2 6 0.95000000 1.00000000

129 zebra_15deg 2 6 0.90000000 1.00000000

130 zebra_15deg 2 6 0.85000000 1.00000000

131 zebra_15deg 2 6 0.80000000 1.00000000

132 zebra_15deg 2 6 0.75000000 0.80000000

133 zebra_15deg 2 6 0.70000000 0.80000000

134 zebra_15deg 2 6 0.65000000 0.80000000

135 zebra_15deg 2 6 0.60000000 0.80000000

136 zebra_15deg 2 6 0.55000000 0.70526316

137 zebra_15deg 2 6 0.50000000 0.70526316

138 zebra_15deg 2 6 0.45000000 0.70526316

139 zebra_15deg 2 6 0.40000000 0.70526316

140 zebra_15deg 2 6 0.35000000 0.64210526

141 zebra_15deg 2 6 0.30000000 0.64210526

142 zebra_15deg 2 6 0.25000000 0.48421053

143 zebra_15deg 2 6 0.20000000 0.48421053

144 zebra_15deg 2 6 0.15000000 0.04210526

145 zebra_15deg 2 6 0.10000000 0.04210526

146 zebra_15deg 2 6 0.05000000 0.00000000

147 zebra_15deg 2 6 0.00000000 0.00000000

148 zebra_15deg 2 7 1.00000000 1.00000000

149 zebra_15deg 2 7 0.95000000 1.00000000

150 zebra_15deg 2 7 0.90000000 1.00000000

151 zebra_15deg 2 7 0.85000000 1.00000000

152 zebra_15deg 2 7 0.80000000 1.00000000

153 zebra_15deg 2 7 0.75000000 1.00000000

154 zebra_15deg 2 7 0.70000000 0.86666667

155 zebra_15deg 2 7 0.65000000 0.86666667

156 zebra_15deg 2 7 0.60000000 0.86666667

157 zebra_15deg 2 7 0.55000000 0.86666667

158 zebra_15deg 2 7 0.50000000 0.86666667

159 zebra_15deg 2 7 0.45000000 0.86666667

160 zebra_15deg 2 7 0.40000000 0.83333333

161 zebra_15deg 2 7 0.35000000 0.83333333

162 zebra_15deg 2 7 0.30000000 0.83333333

163 zebra_15deg 2 7 0.25000000 0.83333333

164 zebra_15deg 2 7 0.20000000 0.83333333

165 zebra_15deg 2 7 0.15000000 0.80000000

166 zebra_15deg 2 7 0.10000000 0.73333333

167 zebra_15deg 2 7 0.05000000 0.48333333

168 zebra_15deg 2 7 0.00000000 0.00000000

169 zebra_15deg 2 8 1.00000000 1.00000000

170 zebra_15deg 2 8 0.95000000 1.00000000

171 zebra_15deg 2 8 0.90000000 1.00000000

172 zebra_15deg 2 8 0.85000000 1.00000000

173 zebra_15deg 2 8 0.80000000 1.00000000

174 zebra_15deg 2 8 0.75000000 1.00000000

175 zebra_15deg 2 8 0.70000000 1.00000000

176 zebra_15deg 2 8 0.65000000 1.00000000

177 zebra_15deg 2 8 0.60000000 1.00000000

178 zebra_15deg 2 8 0.55000000 1.00000000

179 zebra_15deg 2 8 0.50000000 0.88607595

180 zebra_15deg 2 8 0.45000000 0.88607595

181 zebra_15deg 2 8 0.40000000 0.88607595

182 zebra_15deg 2 8 0.35000000 0.72151899

183 zebra_15deg 2 8 0.30000000 0.72151899

184 zebra_15deg 2 8 0.25000000 0.72151899

185 zebra_15deg 2 8 0.20000000 0.72151899

186 zebra_15deg 2 8 0.15000000 0.63291139

187 zebra_15deg 2 8 0.10000000 0.21518987

188 zebra_15deg 2 8 0.05000000 0.11392405

189 zebra_15deg 2 8 0.00000000 0.00000000

190 zebra_15deg 2 9 1.00000000 1.00000000

191 zebra_15deg 2 9 0.95000000 1.00000000

192 zebra_15deg 2 9 0.90000000 1.00000000

193 zebra_15deg 2 9 0.85000000 1.00000000

194 zebra_15deg 2 9 0.80000000 1.00000000

195 zebra_15deg 2 9 0.75000000 1.00000000

196 zebra_15deg 2 9 0.70000000 1.00000000

197 zebra_15deg 2 9 0.65000000 1.00000000

198 zebra_15deg 2 9 0.60000000 1.00000000

199 zebra_15deg 2 9 0.55000000 0.88679245

200 zebra_15deg 2 9 0.50000000 0.71069182

201 zebra_15deg 2 9 0.45000000 0.64150943

202 zebra_15deg 2 9 0.40000000 0.59119497

203 zebra_15deg 2 9 0.35000000 0.59119497

204 zebra_15deg 2 9 0.30000000 0.49685535

205 zebra_15deg 2 9 0.25000000 0.49685535

206 zebra_15deg 2 9 0.20000000 0.39622641

207 zebra_15deg 2 9 0.15000000 0.05031447

208 zebra_15deg 2 9 0.10000000 0.01886793

209 zebra_15deg 2 9 0.05000000 0.01886793

210 zebra_15deg 2 9 0.00000000 0.00000000

211 zebra_15deg 2 10 1.00000000 1.00000000

212 zebra_15deg 2 10 0.95000000 1.00000000

213 zebra_15deg 2 10 0.90000000 1.00000000

214 zebra_15deg 2 10 0.85000000 1.00000000

215 zebra_15deg 2 10 0.80000000 1.00000000

216 zebra_15deg 2 10 0.75000000 1.00000000

217 zebra_15deg 2 10 0.70000000 1.00000000

218 zebra_15deg 2 10 0.65000000 0.35526316

219 zebra_15deg 2 10 0.60000000 0.35526316

220 zebra_15deg 2 10 0.55000000 0.35526316

221 zebra_15deg 2 10 0.50000000 0.35526316

222 zebra_15deg 2 10 0.45000000 0.35526316

223 zebra_15deg 2 10 0.40000000 0.35526316

224 zebra_15deg 2 10 0.35000000 0.23684210

225 zebra_15deg 2 10 0.30000000 0.23684210

226 zebra_15deg 2 10 0.25000000 0.25000000

227 zebra_15deg 2 10 0.20000000 0.19736842

228 zebra_15deg 2 10 0.15000000 0.15789474

229 zebra_15deg 2 10 0.10000000 0.13157895

230 zebra_15deg 2 10 0.05000000 0.07894737

231 zebra_15deg 2 10 0.00000000 0.00000000

232 zebra_15deg 2 11 1.00000000 1.00000000

233 zebra_15deg 2 11 0.95000000 1.00000000

234 zebra_15deg 2 11 0.90000000 1.00000000

235 zebra_15deg 2 11 0.85000000 1.00000000

236 zebra_15deg 2 11 0.80000000 1.00000000

237 zebra_15deg 2 11 0.75000000 0.93023256

238 zebra_15deg 2 11 0.70000000 0.93023256

239 zebra_15deg 2 11 0.65000000 0.93023256

240 zebra_15deg 2 11 0.60000000 0.74418605

241 zebra_15deg 2 11 0.55000000 0.74418605

242 zebra_15deg 2 11 0.50000000 0.74418605

243 zebra_15deg 2 11 0.45000000 0.74418605

244 zebra_15deg 2 11 0.40000000 0.74418605

245 zebra_15deg 2 11 0.35000000 0.59302326

246 zebra_15deg 2 11 0.30000000 0.26744186

247 zebra_15deg 2 11 0.25000000 0.26744186

248 zebra_15deg 2 11 0.20000000 0.19767442

249 zebra_15deg 2 11 0.15000000 0.19767442

250 zebra_15deg 2 11 0.10000000 0.17441861

251 zebra_15deg 2 11 0.05000000 0.03488372

252 zebra_15deg 2 11 0.00000000 0.00000000

253 zebra_25deg 3 1 1.00000000 1.00000000

254 zebra_25deg 3 1 0.95000000 1.00000000

255 zebra_25deg 3 1 0.90000000 1.00000000

256 zebra_25deg 3 1 0.85000000 1.00000000

257 zebra_25deg 3 1 0.80000000 1.00000000

258 zebra_25deg 3 1 0.75000000 0.88541667

259 zebra_25deg 3 1 0.70000000 0.88541667

260 zebra_25deg 3 1 0.65000000 0.88541667

261 zebra_25deg 3 1 0.60000000 0.88541667

262 zebra_25deg 3 1 0.55000000 0.83333333

263 zebra_25deg 3 1 0.50000000 0.83333333

264 zebra_25deg 3 1 0.45000000 0.72916667

265 zebra_25deg 3 1 0.40000000 0.72916667

266 zebra_25deg 3 1 0.35000000 0.72916667

267 zebra_25deg 3 1 0.30000000 0.72916667

268 zebra_25deg 3 1 0.25000000 0.72916667

269 zebra_25deg 3 1 0.20000000 0.63541667

270 zebra_25deg 3 1 0.15000000 0.59375000

271 zebra_25deg 3 1 0.10000000 0.43750000

272 zebra_25deg 3 1 0.05000000 0.00000000

273 zebra_25deg 3 1 0.00000000 0.00000000

274 zebra_25deg 3 2 1.00000000 1.00000000

275 zebra_25deg 3 2 0.95000000 1.00000000

276 zebra_25deg 3 2 0.90000000 1.00000000

277 zebra_25deg 3 2 0.85000000 1.00000000

278 zebra_25deg 3 2 0.80000000 0.93798450

279 zebra_25deg 3 2 0.75000000 0.84496124

280 zebra_25deg 3 2 0.70000000 0.84496124

281 zebra_25deg 3 2 0.65000000 0.84496124

282 zebra_25deg 3 2 0.60000000 0.84496124

283 zebra_25deg 3 2 0.55000000 0.71317829

284 zebra_25deg 3 2 0.50000000 0.62790698

285 zebra_25deg 3 2 0.45000000 0.62790698

286 zebra_25deg 3 2 0.40000000 0.52713178

287 zebra_25deg 3 2 0.35000000 0.17054264

288 zebra_25deg 3 2 0.30000000 0.00000000

289 zebra_25deg 3 2 0.25000000 0.00000000

290 zebra_25deg 3 2 0.20000000 0.00000000

291 zebra_25deg 3 2 0.15000000 0.00000000

292 zebra_25deg 3 2 0.10000000 0.00000000

293 zebra_25deg 3 2 0.05000000 0.00000000

294 zebra_25deg 3 2 0.00000000 0.00000000

295 zebra_25deg 3 3 1.00000000 1.00000000

296 zebra_25deg 3 3 0.95000000 1.00000000

297 zebra_25deg 3 3 0.90000000 0.83593750

298 zebra_25deg 3 3 0.85000000 0.83593750

299 zebra_25deg 3 3 0.80000000 0.83593750

300 zebra_25deg 3 3 0.75000000 0.83593750

301 zebra_25deg 3 3 0.70000000 0.72656250

302 zebra_25deg 3 3 0.65000000 0.72656250

303 zebra_25deg 3 3 0.60000000 0.72656250

304 zebra_25deg 3 3 0.55000000 0.72656250

305 zebra_25deg 3 3 0.50000000 0.60937500

306 zebra_25deg 3 3 0.45000000 0.60937500

307 zebra_25deg 3 3 0.40000000 0.60937500

308 zebra_25deg 3 3 0.35000000 0.51562500

309 zebra_25deg 3 3 0.30000000 0.51562500

310 zebra_25deg 3 3 0.25000000 0.51562500

311 zebra_25deg 3 3 0.20000000 0.42187500

312 zebra_25deg 3 3 0.15000000 0.00000000

313 zebra_25deg 3 3 0.10000000 0.00000000

314 zebra_25deg 3 3 0.05000000 0.00000000

315 zebra_25deg 3 3 0.00000000 0.00000000

316 zebra_25deg 3 4 1.00000000 1.00000000

317 zebra_25deg 3 4 0.95000000 1.00000000

318 zebra_25deg 3 4 0.90000000 1.00000000

319 zebra_25deg 3 4 0.85000000 0.75824176

320 zebra_25deg 3 4 0.80000000 0.75824176

321 zebra_25deg 3 4 0.75000000 0.75824176

322 zebra_25deg 3 4 0.70000000 0.75824176

323 zebra_25deg 3 4 0.65000000 0.65384615

324 zebra_25deg 3 4 0.60000000 0.65384615

325 zebra_25deg 3 4 0.55000000 0.65384615

326 zebra_25deg 3 4 0.50000000 0.69780220

327 zebra_25deg 3 4 0.45000000 0.69780220

328 zebra_25deg 3 4 0.40000000 0.69780220

329 zebra_25deg 3 4 0.35000000 0.69780220

330 zebra_25deg 3 4 0.30000000 0.69780220

331 zebra_25deg 3 4 0.25000000 0.69780220

332 zebra_25deg 3 4 0.20000000 0.69780220

333 zebra_25deg 3 4 0.15000000 0.48901099

334 zebra_25deg 3 4 0.10000000 0.48901099

335 zebra_25deg 3 4 0.05000000 0.23626374

336 zebra_25deg 3 4 0.00000000 0.00000000

337 zebra_25deg 3 5 1.00000000 1.00000000

338 zebra_25deg 3 5 0.95000000 1.00000000

339 zebra_25deg 3 5 0.90000000 1.00000000

340 zebra_25deg 3 5 0.85000000 1.00000000

341 zebra_25deg 3 5 0.80000000 1.00000000

342 zebra_25deg 3 5 0.75000000 1.00000000

343 zebra_25deg 3 5 0.70000000 0.93687708

344 zebra_25deg 3 5 0.65000000 0.93687708

345 zebra_25deg 3 5 0.60000000 0.93687708

346 zebra_25deg 3 5 0.55000000 0.93687708

347 zebra_25deg 3 5 0.50000000 0.84053156

348 zebra_25deg 3 5 0.45000000 0.84053156

349 zebra_25deg 3 5 0.40000000 0.84053156

350 zebra_25deg 3 5 0.35000000 0.84053156

351 zebra_25deg 3 5 0.30000000 0.84053156

352 zebra_25deg 3 5 0.25000000 0.84053156

353 zebra_25deg 3 5 0.20000000 0.68438538

354 zebra_25deg 3 5 0.15000000 0.51827243

355 zebra_25deg 3 5 0.10000000 0.41860465

356 zebra_25deg 3 5 0.05000000 0.00000000

357 zebra_25deg 3 5 0.00000000 0.00000000

358 Upogebia_crab 4 1 1.00000000 0.94029851

359 Upogebia_crab 4 1 0.87577640 0.94029851

360 Upogebia_crab 4 1 0.77639752 0.94029851

361 Upogebia_crab 4 1 0.67701863 1.00000000

362 Upogebia_crab 4 1 0.59006211 0.97014925

363 Upogebia_crab 4 1 0.50931677 0.94029851

364 Upogebia_crab 4 1 0.42857143 0.94029851

365 Upogebia_crab 4 1 0.34161491 0.94029851

366 Upogebia_crab 4 1 0.30434783 0.92537313

367 Upogebia_crab 4 1 0.29192547 0.91044776

368 Upogebia_crab 4 1 0.28571429 0.86567164

369 Upogebia_crab 4 1 0.24223602 0.86567164

370 Upogebia_crab 4 1 0.22981367 0.82089552

371 Upogebia_crab 4 1 0.14906832 0.71641791

372 Upogebia_crab 4 1 0.12422360 0.52238806

373 Upogebia_crab 4 1 0.09316770 0.49253731

374 Upogebia_crab 4 1 0.05590062 0.35820895

375 Upogebia_crab 4 1 0.02484472 0.28358209

376 Upogebia_crab 4 1 0.01242236 0.13432836

377 Upogebia_crab 4 1 0.00000000 0.00000000

378 Upogebia_crab 4 2 1.00000000 1.00000000

379 Upogebia_crab 4 2 0.95394737 0.70454545

380 Upogebia_crab 4 2 0.87500000 0.68181818

381 Upogebia_crab 4 2 0.82894737 0.68181818

382 Upogebia_crab 4 2 0.80921053 0.68181818

383 Upogebia_crab 4 2 0.69078947 0.69318182

384 Upogebia_crab 4 2 0.57236842 0.69318182

385 Upogebia_crab 4 2 0.50657895 0.68181818

386 Upogebia_crab 4 2 0.38815789 0.68181818

387 Upogebia_crab 4 2 0.35526316 0.68181818

388 Upogebia_crab 4 2 0.32236842 0.61363636

389 Upogebia_crab 4 2 0.28289474 0.53409091

390 Upogebia_crab 4 2 0.23684210 0.54545455

391 Upogebia_crab 4 2 0.19078947 0.50000000

392 Upogebia_crab 4 2 0.14473684 0.36363636

393 Upogebia_crab 4 2 0.07236842 0.23863636

394 Upogebia_crab 4 2 0.05921053 0.15909091

395 Upogebia_crab 4 2 0.03289474 0.11363636

396 Upogebia_crab 4 2 0.01315789 0.07954546

397 Upogebia_crab 4 2 0.00000000 0.00000000

398 Upogebia_crab 4 3 1.00000000 1.00000000

399 Upogebia_crab 4 3 0.98639456 0.87804878

400 Upogebia_crab 4 3 0.93197279 0.87804878

401 Upogebia_crab 4 3 0.88435374 0.87804878

402 Upogebia_crab 4 3 0.80952381 0.87804878

403 Upogebia_crab 4 3 0.70068027 0.87804878

404 Upogebia_crab 4 3 0.60544218 0.87804878

405 Upogebia_crab 4 3 0.51700680 0.87804878

406 Upogebia_crab 4 3 0.44217687 0.87804878

407 Upogebia_crab 4 3 0.42176871 0.80487805

408 Upogebia_crab 4 3 0.38095238 0.74390244

409 Upogebia_crab 4 3 0.33333333 0.67073171

410 Upogebia_crab 4 3 0.29251701 0.60975610

411 Upogebia_crab 4 3 0.25850340 0.54878049

412 Upogebia_crab 4 3 0.19047619 0.48780488

413 Upogebia_crab 4 3 0.14965986 0.41463415

414 Upogebia_crab 4 3 0.10204082 0.36585366

415 Upogebia_crab 4 3 0.08843537 0.30487805

416 Upogebia_crab 4 3 0.06122449 0.24390244

417 Upogebia_crab 4 3 0.04761905 0.20731707

418 Upogebia_crab 4 3 0.02721088 0.10975610

419 Upogebia_crab 4 3 0.01360544 0.06097561

420 Upogebia_crab 4 3 0.00000000 0.00000000

421 Upogebia_crab 4 4 1.00000000 1.00000000

422 Upogebia_crab 4 4 0.93835616 0.84415584

423 Upogebia_crab 4 4 0.75342466 0.80519480

424 Upogebia_crab 4 4 0.59589041 0.81818182

425 Upogebia_crab 4 4 0.49315068 0.81818182

426 Upogebia_crab 4 4 0.43150685 0.81818182

427 Upogebia_crab 4 4 0.35616438 0.80519480

428 Upogebia_crab 4 4 0.34931507 0.77922078

429 Upogebia_crab 4 4 0.29452055 0.70129870

430 Upogebia_crab 4 4 0.23972603 0.54545455

431 Upogebia_crab 4 4 0.19863014 0.48051948

432 Upogebia_crab 4 4 0.11643836 0.40259740

433 Upogebia_crab 4 4 0.09589041 0.28571429

434 Upogebia_crab 4 4 0.07534247 0.19480519

435 Upogebia_crab 4 4 0.05479452 0.16883117

436 Upogebia_crab 4 4 0.04794520 0.09090909

437 Upogebia_crab 4 4 0.02739726 0.09090909

438 Upogebia_crab 4 4 0.01369863 0.05194805

439 Upogebia_crab 4 4 0.00000000 0.00000000

440 P_serratus 5 1 1.00000000 1.00000000

441 P_serratus 5 1 0.93548387 0.96815287

442 P_serratus 5 1 0.86559140 0.98726115

443 P_serratus 5 1 0.80645161 0.98089172

444 P_serratus 5 1 0.73655914 0.96178344

445 P_serratus 5 1 0.67204301 0.97452229

446 P_serratus 5 1 0.60752688 0.96815287

447 P_serratus 5 1 0.53763441 0.98089172

448 P_serratus 5 1 0.47311828 0.99363057

449 P_serratus 5 1 0.40860215 0.98089172

450 P_serratus 5 1 0.33870968 0.94267516

451 P_serratus 5 1 0.26881720 0.85350318

452 P_serratus 5 1 0.20430107 0.77070064

453 P_serratus 5 1 0.13440860 0.59235669

454 P_serratus 5 1 0.06989247 0.37579618

455 P_serratus 5 1 0.00000000 0.00000000

456 P_serratus 5 2 1.00000000 0.98064516

457 P_serratus 5 2 0.93548387 1.00000000

458 P_serratus 5 2 0.86559140 0.96774194

459 P_serratus 5 2 0.80645161 0.92903226

460 P_serratus 5 2 0.73655914 0.95483871

461 P_serratus 5 2 0.67204301 0.95483871

462 P_serratus 5 2 0.60752688 0.96774194

463 P_serratus 5 2 0.53763441 0.96774194

464 P_serratus 5 2 0.47311828 0.94838710

465 P_serratus 5 2 0.40860215 0.94193548

466 P_serratus 5 2 0.33870968 0.88387097

467 P_serratus 5 2 0.26881720 0.81290323

468 P_serratus 5 2 0.20430107 0.79354839

469 P_serratus 5 2 0.13440860 0.66451613

470 P_serratus 5 2 0.06989247 0.35483871

471 P_serratus 5 2 0.00000000 0.00000000

472 P_elegans 6 1 1.00000000 0.97241379

473 P_elegans 6 1 0.92473118 0.95172414

474 P_elegans 6 1 0.86021505 0.97241379

475 P_elegans 6 1 0.79569892 0.96551724

476 P_elegans 6 1 0.73118280 0.95862069

477 P_elegans 6 1 0.67204301 0.95172414

478 P_elegans 6 1 0.60752688 0.93103448

479 P_elegans 6 1 0.53763441 0.92413793

480 P_elegans 6 1 0.47849462 0.94482759

481 P_elegans 6 1 0.40860215 0.93103448

482 P_elegans 6 1 0.34408602 1.00000000

483 P_elegans 6 1 0.27956989 0.94482759

484 P_elegans 6 1 0.20430107 0.94482759

485 P_elegans 6 1 0.13978495 0.68275862

486 P_elegans 6 1 0.06451613 0.48965517

487 P_elegans 6 1 0.00000000 0.00000000

488 P_elegans 6 2 1.00000000 0.92957747

489 P_elegans 6 2 0.92473118 0.91549296

490 P_elegans 6 2 0.86021505 0.92957747

491 P_elegans 6 2 0.79569892 0.91549296

492 P_elegans 6 2 0.73118280 0.92253521

493 P_elegans 6 2 0.67204301 0.92253521

494 P_elegans 6 2 0.60752688 0.92253521

495 P_elegans 6 2 0.53763441 0.94366197

496 P_elegans 6 2 0.47849462 0.99295775

497 P_elegans 6 2 0.40860215 1.00000000

498 P_elegans 6 2 0.34408602 0.95070422

499 P_elegans 6 2 0.27956989 0.95070422

500 P_elegans 6 2 0.20430107 0.80281690

501 P_elegans 6 2 0.13978495 0.63380282

502 P_elegans 6 2 0.06451613 0.44366197

503 P_elegans 6 2 0.00000000 0.00000000

504 N_virens 7 1 1.00000000 1.00000000

505 N_virens 7 1 0.88888889 0.88659794

506 N_virens 7 1 0.77777778 0.84536082

507 N_virens 7 1 0.66666667 0.74226804

508 N_virens 7 1 0.55555556 0.59793814

509 N_virens 7 1 0.44444444 0.55670103

510 N_virens 7 1 0.33333333 0.49484536

511 N_virens 7 1 0.22222222 0.36082474

512 N_virens 7 1 0.11111111 0.27835052

513 N_virens 7 1 0.05555556 0.21649484

514 N_virens 7 1 0.00000000 0.00000000

515 N_virens 7 2 1.00000000 1.00000000

516 N_virens 7 2 0.88888889 0.91566265

517 N_virens 7 2 0.77777778 0.78313253

518 N_virens 7 2 0.66666667 0.74698795

519 N_virens 7 2 0.55555556 0.57831325

520 N_virens 7 2 0.44444444 0.54216867

521 N_virens 7 2 0.33333333 0.45783133

522 N_virens 7 2 0.22222222 0.32530120

523 N_virens 7 2 0.11111111 0.26506024

524 N_virens 7 2 0.05555556 0.16867470

525 N_virens 7 2 0.00000000 0.00000000

526 N_virens 7 3 1.00000000 1.00000000

527 N_virens 7 3 0.88888889 0.91549296

528 N_virens 7 3 0.77777778 0.81690141

529 N_virens 7 3 0.66666667 0.74647887

530 N_virens 7 3 0.55555556 0.47887324

531 N_virens 7 3 0.44444444 0.35211268

532 N_virens 7 3 0.33333333 0.26760563

533 N_virens 7 3 0.22222222 0.23943662

534 N_virens 7 3 0.11111111 0.21126761

535 N_virens 7 3 0.05555556 0.11267606

536 N_virens 7 3 0.00000000 0.00000000

537 N_virens 7 4 1.00000000 1.00000000

538 N_virens 7 4 0.88888889 0.87692308

539 N_virens 7 4 0.77777778 0.83076923

540 N_virens 7 4 0.66666667 0.75384615

541 N_virens 7 4 0.55555556 0.66153846

542 N_virens 7 4 0.44444444 0.56923077

543 N_virens 7 4 0.33333333 0.49230769

544 N_virens 7 4 0.22222222 0.35384615

545 N_virens 7 4 0.11111111 0.18461538

546 N_virens 7 4 0.05555556 0.13846154

547 N_virens 7 4 0.00000000 0.00000000

548 N_virens 7 5 1.00000000 1.00000000

549 N_virens 7 5 0.88888889 0.82758621

550 N_virens 7 5 0.77777778 0.77586207

551 N_virens 7 5 0.66666667 0.74137931

552 N_virens 7 5 0.55555556 0.65517241

553 N_virens 7 5 0.44444444 0.50000000

554 N_virens 7 5 0.33333333 0.43103448

555 N_virens 7 5 0.22222222 0.32758621

556 N_virens 7 5 0.11111111 0.15517241

557 N_virens 7 5 0.05555556 0.08620690

558 N_virens 7 5 0.00000000 0.00000000

559 N_diversicolor 8 1 1.00000000 1.00000000

560 N_diversicolor 8 1 0.88888889 0.87500000

561 N_diversicolor 8 1 0.77777778 0.81730769

562 N_diversicolor 8 1 0.66666667 0.75000000

563 N_diversicolor 8 1 0.55555556 0.64423077

564 N_diversicolor 8 1 0.44444444 0.60576923

565 N_diversicolor 8 1 0.33333333 0.47115385

566 N_diversicolor 8 1 0.22222222 0.39423077

567 N_diversicolor 8 1 0.11111111 0.27884615

568 N_diversicolor 8 1 0.05555556 0.21153846

569 N_diversicolor 8 1 0.00000000 0.00000000

570 N_diversicolor 8 2 1.00000000 1.00000000

571 N_diversicolor 8 2 0.88888889 0.85263158

572 N_diversicolor 8 2 0.77777778 0.66315789

573 N_diversicolor 8 2 0.66666667 0.61052632

574 N_diversicolor 8 2 0.55555556 0.55789474

575 N_diversicolor 8 2 0.44444444 0.51578947

576 N_diversicolor 8 2 0.33333333 0.40000000

577 N_diversicolor 8 2 0.22222222 0.31578947

578 N_diversicolor 8 2 0.11111111 0.28421053

579 N_diversicolor 8 2 0.05555556 0.18947368

580 N_diversicolor 8 2 0.00000000 0.00000000

581 N_diversicolor 8 3 1.00000000 1.00000000

582 N_diversicolor 8 3 0.88888889 0.82758621

583 N_diversicolor 8 3 0.77777778 0.79310345

584 N_diversicolor 8 3 0.66666667 0.72413793

585 N_diversicolor 8 3 0.55555556 0.62068965

586 N_diversicolor 8 3 0.44444444 0.50574713

587 N_diversicolor 8 3 0.33333333 0.39080460

588 N_diversicolor 8 3 0.22222222 0.31034483

589 N_diversicolor 8 3 0.11111111 0.19540230

590 N_diversicolor 8 3 0.05555556 0.09195402

591 N_diversicolor 8 3 0.00000000 0.00000000

592 N_diversicolor 8 4 1.00000000 1.00000000

593 N_diversicolor 8 4 0.88888889 0.89062500

594 N_diversicolor 8 4 0.77777778 0.82812500

595 N_diversicolor 8 4 0.66666667 0.73437500

596 N_diversicolor 8 4 0.55555556 0.71875000

597 N_diversicolor 8 4 0.44444444 0.57812500

598 N_diversicolor 8 4 0.33333333 0.42187500

599 N_diversicolor 8 4 0.22222222 0.35937500

600 N_diversicolor 8 4 0.11111111 0.32812500

601 N_diversicolor 8 4 0.05555556 0.20312500

602 N_diversicolor 8 4 0.00000000 0.00000000

603 N_aciculata 9 1 1.00000000 1.00000000

604 N_aciculata 9 1 0.88888889 0.73255814

605 N_aciculata 9 1 0.77777778 0.61627907

606 N_aciculata 9 1 0.66666667 0.51162791

607 N_aciculata 9 1 0.55555556 0.47674419

608 N_aciculata 9 1 0.44444444 0.44186046

609 N_aciculata 9 1 0.33333333 0.37209302

610 N_aciculata 9 1 0.22222222 0.30232558

611 N_aciculata 9 1 0.11111111 0.16279070

612 N_aciculata 9 1 0.05555556 0.09302326

613 N_aciculata 9 1 0.00000000 0.00000000

614 N_aciculata 9 2 1.00000000 1.00000000

615 N_aciculata 9 2 0.88888889 0.97260274

616 N_aciculata 9 2 0.77777778 0.78082192

617 N_aciculata 9 2 0.66666667 0.58904110

618 N_aciculata 9 2 0.55555556 0.57534247

619 N_aciculata 9 2 0.44444444 0.57534247

620 N_aciculata 9 2 0.33333333 0.39726027

621 N_aciculata 9 2 0.22222222 0.28767123

622 N_aciculata 9 2 0.11111111 0.23287671

623 N_aciculata 9 2 0.05555556 0.15068493

624 N_aciculata 9 2 0.00000000 0.00000000

625 N_aciculata 9 3 1.00000000 1.00000000

626 N_aciculata 9 3 0.88888889 0.86440678

627 N_aciculata 9 3 0.77777778 0.72881356

628 N_aciculata 9 3 0.66666667 0.47457627

629 N_aciculata 9 3 0.55555556 0.45762712

630 N_aciculata 9 3 0.44444444 0.35593220

631 N_aciculata 9 3 0.33333333 0.23728814

632 N_aciculata 9 3 0.22222222 0.16949153

633 N_aciculata 9 3 0.11111111 0.13559322

634 N_aciculata 9 3 0.05555556 0.10169491

635 N_aciculata 9 3 0.00000000 0.00000000

636 N_aciculata 9 4 1.00000000 1.00000000

637 N_aciculata 9 4 0.88888889 0.75438596

638 N_aciculata 9 4 0.77777778 0.71929825

639 N_aciculata 9 4 0.66666667 0.61403509

640 N_aciculata 9 4 0.55555556 0.49122807

641 N_aciculata 9 4 0.44444444 0.49122807

642 N_aciculata 9 4 0.33333333 0.31578947

643 N_aciculata 9 4 0.22222222 0.24561404

644 N_aciculata 9 4 0.11111111 0.14035088

645 N_aciculata 9 4 0.05555556 0.12280702

646 N_aciculata 9 4 0.00000000 0.00000000

647 A_marina 10 1 1.00000000 1.00000000

648 A_marina 10 1 0.88888889 0.87777778

649 A_marina 10 1 0.77777778 0.84444444

650 A_marina 10 1 0.66666667 0.70000000

651 A_marina 10 1 0.55555556 0.54444444

652 A_marina 10 1 0.44444444 0.46666667

653 A_marina 10 1 0.33333333 0.44444444

654 A_marina 10 1 0.22222222 0.34444444

655 A_marina 10 1 0.11111111 0.28888889

656 A_marina 10 1 0.05555556 0.22222222

657 A_marina 10 1 0.00000000 0.00000000

658 A_marina 10 2 1.00000000 1.00000000

659 A_marina 10 2 0.88888889 0.89411765

660 A_marina 10 2 0.77777778 0.81176471

661 A_marina 10 2 0.66666667 0.80000000

662 A_marina 10 2 0.55555556 0.67058823

663 A_marina 10 2 0.44444444 0.54117647

664 A_marina 10 2 0.33333333 0.44705882

665 A_marina 10 2 0.22222222 0.43529412

666 A_marina 10 2 0.11111111 0.28235294

667 A_marina 10 2 0.05555556 0.18823529

668 A_marina 10 2 0.00000000 0.00000000

669 A_marina 10 3 1.00000000 1.00000000

670 A_marina 10 3 0.88888889 0.88157895

671 A_marina 10 3 0.77777778 0.80263158

672 A_marina 10 3 0.66666667 0.68421053

673 A_marina 10 3 0.55555556 0.46052632

674 A_marina 10 3 0.44444444 0.38157895

675 A_marina 10 3 0.33333333 0.36842105

676 A_marina 10 3 0.22222222 0.23684210

677 A_marina 10 3 0.11111111 0.22368421

678 A_marina 10 3 0.05555556 0.17105263

679 A_marina 10 3 0.00000000 0.00000000

680 A_marina 10 4 1.00000000 1.00000000

681 A_marina 10 4 0.88888889 0.91304348

682 A_marina 10 4 0.77777778 0.79710145

683 A_marina 10 4 0.66666667 0.71014493

684 A_marina 10 4 0.55555556 0.56521739

685 A_marina 10 4 0.44444444 0.52173913

686 A_marina 10 4 0.33333333 0.36231884

687 A_marina 10 4 0.22222222 0.31884058

688 A_marina 10 4 0.11111111 0.20289855

689 A_marina 10 4 0.05555556 0.15942029

690 A_marina 10 4 0.00000000 0.00000000

691 epaulette_sharks 11 1 1.00000000 0.87719298

692 epaulette_sharks 11 1 0.96330275 0.84210526

693 epaulette_sharks 11 1 0.91743119 0.92982456

694 epaulette_sharks 11 1 0.87614679 0.94736842

695 epaulette_sharks 11 1 0.82568807 0.94736842

696 epaulette_sharks 11 1 0.78899083 0.80701754

697 epaulette_sharks 11 1 0.74311927 0.98245614

698 epaulette_sharks 11 1 0.70183486 0.91228070

699 epaulette_sharks 11 1 0.66055046 0.84210526

700 epaulette_sharks 11 1 0.61926605 0.98245614

701 epaulette_sharks 11 1 0.57339449 0.84210526

702 epaulette_sharks 11 1 0.54128440 1.00000000

703 epaulette_sharks 11 1 0.49541284 0.85964912

704 epaulette_sharks 11 1 0.44954128 0.96491228

705 epaulette_sharks 11 1 0.40366972 0.92982456

706 epaulette_sharks 11 1 0.36238532 0.96491228

707 epaulette_sharks 11 1 0.33027523 0.85964912

708 epaulette_sharks 11 1 0.28440367 0.82456140

709 epaulette_sharks 11 1 0.22477064 0.70175439

710 epaulette_sharks 11 1 0.19266055 0.66666667

711 epaulette_sharks 11 1 0.16513761 0.64912281

712 epaulette_sharks 11 1 0.12844037 0.49122807

713 epaulette_sharks 11 1 0.00000000 0.00000000

714 epaulette_sharks 11 2 1.00000000 0.98701299

715 epaulette_sharks 11 2 0.93750000 0.87012987

716 epaulette_sharks 11 2 0.86979167 0.97402597

717 epaulette_sharks 11 2 0.81770833 0.83116883

718 epaulette_sharks 11 2 0.75000000 0.94805195

719 epaulette_sharks 11 2 0.69270833 0.83116883

720 epaulette_sharks 11 2 0.62500000 0.96103896

721 epaulette_sharks 11 2 0.56250000 0.85714286

722 epaulette_sharks 11 2 0.50000000 0.97402597

723 epaulette_sharks 11 2 0.44270833 0.87012987

724 epaulette_sharks 11 2 0.38020833 1.00000000

725 epaulette_sharks 11 2 0.31770833 0.84415584

726 epaulette_sharks 11 2 0.26562500 0.80519480

727 epaulette_sharks 11 2 0.22395833 0.61038961

728 epaulette_sharks 11 2 0.17187500 0.66233766

729 epaulette_sharks 11 2 0.13020833 0.33766234

730 epaulette_sharks 11 2 0.10937500 0.29870130

731 epaulette_sharks 11 2 0.07291667 0.10389610

732 epaulette_sharks 11 2 0.05729167 0.03896104

733 epaulette_sharks 11 2 0.04687500 0.00000000

734 epaulette_sharks 11 2 0.00000000 0.00000000

735 epaulette_sharks 11 3 1.00000000 0.90909091

736 epaulette_sharks 11 3 0.92982456 0.97402597

737 epaulette_sharks 11 3 0.86549708 0.94805195

738 epaulette_sharks 11 3 0.79532164 0.92207792

739 epaulette_sharks 11 3 0.73099415 0.97402597

740 epaulette_sharks 11 3 0.66081871 1.00000000

741 epaulette_sharks 11 3 0.59649123 0.93506493

742 epaulette_sharks 11 3 0.53801170 0.80519480

743 epaulette_sharks 11 3 0.46783626 0.93506493

744 epaulette_sharks 11 3 0.40350877 0.93506493

745 epaulette_sharks 11 3 0.33918129 0.88311688

746 epaulette_sharks 11 3 0.29239766 0.68831169

747 epaulette_sharks 11 3 0.24561404 0.61038961

748 epaulette_sharks 11 3 0.21052632 0.48051948

749 epaulette_sharks 11 3 0.17543860 0.54545455

750 epaulette_sharks 11 3 0.14619883 0.33766234

751 epaulette_sharks 11 3 0.12865497 0.36363636

752 epaulette_sharks 11 3 0.10526316 0.33766234

753 epaulette_sharks 11 3 0.08771930 0.24675325

754 epaulette_sharks 11 3 0.07017544 0.09090909

755 epaulette_sharks 11 3 0.00000000 0.00000000

756 epaulette_sharks 11 4 1.00000000 0.92156863

757 epaulette_sharks 11 4 0.97237569 0.74509804

758 epaulette_sharks 11 4 0.93922652 1.00000000

759 epaulette_sharks 11 4 0.90055249 0.80392157

760 epaulette_sharks 11 4 0.86187845 0.90196078

761 epaulette_sharks 11 4 0.82872928 0.90196078

762 epaulette_sharks 11 4 0.79005525 0.90196078

763 epaulette_sharks 11 4 0.76243094 0.90196078

764 epaulette_sharks 11 4 0.71823204 0.90196078

765 epaulette_sharks 11 4 0.68508287 0.92156863

766 epaulette_sharks 11 4 0.65193370 0.90196078

767 epaulette_sharks 11 4 0.61878453 0.82352941

768 epaulette_sharks 11 4 0.58563536 0.90196078

769 epaulette_sharks 11 4 0.54696133 0.90196078

770 epaulette_sharks 11 4 0.50828729 0.90196078

771 epaulette_sharks 11 4 0.47513812 0.90196078

772 epaulette_sharks 11 4 0.43646409 0.90196078

773 epaulette_sharks 11 4 0.40331492 0.76470588

774 epaulette_sharks 11 4 0.38121547 0.68627451

775 epaulette_sharks 11 4 0.34806630 0.82352941

776 epaulette_sharks 11 4 0.32044199 0.62745098

777 epaulette_sharks 11 4 0.29281768 0.68627451

778 epaulette_sharks 11 4 0.26519337 0.64705882

779 epaulette_sharks 11 4 0.24309392 0.54901961

780 epaulette_sharks 11 4 0.20441989 0.43137255

781 epaulette_sharks 11 4 0.14917127 0.39215686

782 epaulette_sharks 11 4 0.12154696 0.33333333

783 epaulette_sharks 11 4 0.09392265 0.15686274

784 epaulette_sharks 11 4 0.00000000 0.00000000

785 epaulette_sharks 11 5 1.00000000 0.83333333

786 epaulette_sharks 11 5 0.91758242 0.85416667

787 epaulette_sharks 11 5 0.87362637 1.00000000

788 epaulette_sharks 11 5 0.83516484 0.91666667

789 epaulette_sharks 11 5 0.75824176 0.83333333

790 epaulette_sharks 11 5 0.71978022 0.95833333

791 epaulette_sharks 11 5 0.68131868 0.91666667

792 epaulette_sharks 11 5 0.64285714 0.95833333

793 epaulette_sharks 11 5 0.59890110 0.81250000

794 epaulette_sharks 11 5 0.56043956 0.97916667

795 epaulette_sharks 11 5 0.52197802 0.87500000

796 epaulette_sharks 11 5 0.48351648 1.00000000

797 epaulette_sharks 11 5 0.44505494 0.83333333

798 epaulette_sharks 11 5 0.41758242 0.93750000

799 epaulette_sharks 11 5 0.37362637 0.89583333

800 epaulette_sharks 11 5 0.32417582 0.97916667

801 epaulette_sharks 11 5 0.27472527 0.89583333

802 epaulette_sharks 11 5 0.21978022 0.70833333

803 epaulette_sharks 11 5 0.18131868 0.79166667

804 epaulette_sharks 11 5 0.11538461 0.52083333

805 epaulette_sharks 11 5 0.07142857 0.25000000

806 epaulette_sharks 11 5 0.00000000 0.00000000

807 epaulette_sharks 11 6 1.00000000 0.87037037

808 epaulette_sharks 11 6 0.95336788 1.00000000

809 epaulette_sharks 11 6 0.90673575 0.88888889

810 epaulette_sharks 11 6 0.86528497 0.96296296

811 epaulette_sharks 11 6 0.81865285 0.92592593

812 epaulette_sharks 11 6 0.77202072 0.94444444

813 epaulette_sharks 11 6 0.72538860 0.94444444

814 epaulette_sharks 11 6 0.68393782 0.85185185

815 epaulette_sharks 11 6 0.63730570 0.96296296

816 epaulette_sharks 11 6 0.58549223 0.96296296

817 epaulette_sharks 11 6 0.54404145 0.88888889

818 epaulette_sharks 11 6 0.49740933 0.96296296

819 epaulette_sharks 11 6 0.45595855 0.94444444

820 epaulette_sharks 11 6 0.40414508 0.92592593

821 epaulette_sharks 11 6 0.37305700 0.77777778

822 epaulette_sharks 11 6 0.32642487 0.79629630

823 epaulette_sharks 11 6 0.28497409 0.74074074

824 epaulette_sharks 11 6 0.25388601 0.68518519

825 epaulette_sharks 11 6 0.22279793 0.66666667

826 epaulette_sharks 11 6 0.19689119 0.48148148

827 epaulette_sharks 11 6 0.17098446 0.57407407

828 epaulette_sharks 11 6 0.15544041 0.50000000

829 epaulette_sharks 11 6 0.10362694 0.44444444

830 epaulette_sharks 11 6 0.03626943 0.22222222

831 epaulette_sharks 11 6 0.00000000 0.00000000

832 epaulette_sharks 11 7 1.00000000 0.94791667

833 epaulette_sharks 11 7 0.93296089 0.96875000

834 epaulette_sharks 11 7 0.87150838 0.90625000

835 epaulette_sharks 11 7 0.81005587 0.90625000

836 epaulette_sharks 11 7 0.74860335 0.87500000

837 epaulette_sharks 11 7 0.68715084 1.00000000

838 epaulette_sharks 11 7 0.62569832 0.85416667

839 epaulette_sharks 11 7 0.56424581 0.93750000

840 epaulette_sharks 11 7 0.50837989 0.87500000

841 epaulette_sharks 11 7 0.44134078 0.95833333

842 epaulette_sharks 11 7 0.37988827 0.87500000

843 epaulette_sharks 11 7 0.32960894 0.69791667

844 epaulette_sharks 11 7 0.29050279 0.62500000

845 epaulette_sharks 11 7 0.25139665 0.50000000

846 epaulette_sharks 11 7 0.21787709 0.53125000

847 epaulette_sharks 11 7 0.17877095 0.55208333

848 epaulette_sharks 11 7 0.14525140 0.44791667

849 epaulette_sharks 11 7 0.12849162 0.34375000

850 epaulette_sharks 11 7 0.10614525 0.32291667

851 epaulette_sharks 11 7 0.08938548 0.25000000

852 epaulette_sharks 11 7 0.06703911 0.25000000

853 epaulette_sharks 11 7 0.05027933 0.25000000

854 epaulette_sharks 11 7 0.03351955 0.12500000

855 epaulette_sharks 11 7 0.00000000 0.00000000

856 epaulette_sharks 11 8 1.00000000 0.85897436

857 epaulette_sharks 11 8 0.93582888 1.00000000

858 epaulette_sharks 11 8 0.88770054 0.92307692

859 epaulette_sharks 11 8 0.82352941 0.93589744

860 epaulette_sharks 11 8 0.73262032 0.80769231

861 epaulette_sharks 11 8 0.67914438 0.82051282

862 epaulette_sharks 11 8 0.63101604 0.82051282

863 epaulette_sharks 11 8 0.58288770 0.74358974

864 epaulette_sharks 11 8 0.53475936 0.78205128

865 epaulette_sharks 11 8 0.48128342 0.83333333

866 epaulette_sharks 11 8 0.42780749 0.92307692

867 epaulette_sharks 11 8 0.37433155 0.79487180

868 epaulette_sharks 11 8 0.33689840 0.70512820

869 epaulette_sharks 11 8 0.29411765 0.67948718

870 epaulette_sharks 11 8 0.22459893 0.56410256

871 epaulette_sharks 11 8 0.17112299 0.47435897

872 epaulette_sharks 11 8 0.11764706 0.28205128

873 epaulette_sharks 11 8 0.07486631 0.17948718

874 epaulette_sharks 11 8 0.00000000 0.00000000

875 fish_Ammodytes 12 1 1.00000000 1.00000000

876 fish_Ammodytes 12 1 0.93750000 0.96428571

877 fish_Ammodytes 12 1 0.88888889 0.94642857

878 fish_Ammodytes 12 1 0.83333333 0.91071429

879 fish_Ammodytes 12 1 0.77777778 0.87500000

880 fish_Ammodytes 12 1 0.73611111 0.87500000

881 fish_Ammodytes 12 1 0.68750000 0.85714286

882 fish_Ammodytes 12 1 0.63888889 0.83928571

883 fish_Ammodytes 12 1 0.59027778 0.82142857

884 fish_Ammodytes 12 1 0.54861111 0.80357143

885 fish_Ammodytes 12 1 0.50694444 0.82142857

886 fish_Ammodytes 12 1 0.45833333 0.83928571

887 fish_Ammodytes 12 1 0.40972222 0.80357143

888 fish_Ammodytes 12 1 0.37500000 0.78571429

889 fish_Ammodytes 12 1 0.33333333 0.76785714

890 fish_Ammodytes 12 1 0.29166667 0.78571429

891 fish_Ammodytes 12 1 0.25694444 0.76785714

892 fish_Ammodytes 12 1 0.22222222 0.71428571

893 fish_Ammodytes 12 1 0.18750000 0.67857143

894 fish_Ammodytes 12 1 0.17361111 0.67857143

895 fish_Ammodytes 12 1 0.13194444 0.50000000

896 fish_Ammodytes 12 1 0.11805556 0.57142857

897 fish_Ammodytes 12 1 0.10416667 0.46428571

898 fish_Ammodytes 12 1 0.09027778 0.46428571

899 fish_Ammodytes 12 1 0.08333333 0.35714286

900 fish_Ammodytes 12 1 0.06250000 0.32142857

901 fish_Ammodytes 12 1 0.00000000 0.00000000

902 fish_Ammodytes 12 2 1.00000000 0.85454546

903 fish_Ammodytes 12 2 0.95945946 0.87272727

904 fish_Ammodytes 12 2 0.93918919 0.87272727

905 fish_Ammodytes 12 2 0.90540540 0.83636364

906 fish_Ammodytes 12 2 0.87837838 0.89090909

907 fish_Ammodytes 12 2 0.85810811 0.81818182

908 fish_Ammodytes 12 2 0.82432432 0.85454546

909 fish_Ammodytes 12 2 0.79729730 0.85454546

910 fish_Ammodytes 12 2 0.77027027 0.87272727

911 fish_Ammodytes 12 2 0.75000000 0.92727273

912 fish_Ammodytes 12 2 0.72297297 0.83636364

913 fish_Ammodytes 12 2 0.69594595 0.85454546

914 fish_Ammodytes 12 2 0.66216216 0.85454546

915 fish_Ammodytes 12 2 0.63513513 0.87272727

916 fish_Ammodytes 12 2 0.61486487 0.90909091

917 fish_Ammodytes 12 2 0.59459460 0.87272727

918 fish_Ammodytes 12 2 0.56756757 0.87272727

919 fish_Ammodytes 12 2 0.54054054 0.89090909

920 fish_Ammodytes 12 2 0.51351351 0.92727273

921 fish_Ammodytes 12 2 0.48648649 0.89090909

922 fish_Ammodytes 12 2 0.45945946 0.89090909

923 fish_Ammodytes 12 2 0.43243243 0.94545455

924 fish_Ammodytes 12 2 0.41216216 0.90909091

925 fish_Ammodytes 12 2 0.37837838 0.92727273

926 fish_Ammodytes 12 2 0.35135135 0.96363636

927 fish_Ammodytes 12 2 0.33108108 0.96363636

928 fish_Ammodytes 12 2 0.30405405 0.89090909

929 fish_Ammodytes 12 2 0.27702703 0.98181818

930 fish_Ammodytes 12 2 0.25675676 0.94545455

931 fish_Ammodytes 12 2 0.22972973 1.00000000

932 fish_Ammodytes 12 2 0.20270270 0.94545455

933 fish_Ammodytes 12 2 0.18243243 0.85454546

934 fish_Ammodytes 12 2 0.15540540 0.85454546

935 fish_Ammodytes 12 2 0.13513513 0.67272727

936 fish_Ammodytes 12 2 0.12162162 0.60000000

937 fish_Ammodytes 12 2 0.10810811 0.56363636

938 fish_Ammodytes 12 2 0.00000000 0.00000000

939 fish_Ammodytes 12 3 1.00000000 0.96491228

940 fish_Ammodytes 12 3 0.93377483 1.00000000

941 fish_Ammodytes 12 3 0.87417219 0.89473684

942 fish_Ammodytes 12 3 0.81456954 0.91228070

943 fish_Ammodytes 12 3 0.76158940 0.89473684

944 fish_Ammodytes 12 3 0.70198675 0.87719298

945 fish_Ammodytes 12 3 0.64238411 0.89473684

946 fish_Ammodytes 12 3 0.58278146 0.84210526

947 fish_Ammodytes 12 3 0.52980132 0.84210526

948 fish_Ammodytes 12 3 0.47682119 0.87719298

949 fish_Ammodytes 12 3 0.41721854 0.84210526

950 fish_Ammodytes 12 3 0.36423841 0.84210526

951 fish_Ammodytes 12 3 0.31125828 0.82456140

952 fish_Ammodytes 12 3 0.26490066 0.77192982

953 fish_Ammodytes 12 3 0.22516556 0.77192982

954 fish_Ammodytes 12 3 0.20529801 0.77192982

955 fish_Ammodytes 12 3 0.17880795 0.75438596

956 fish_Ammodytes 12 3 0.15894040 0.71929825

957 fish_Ammodytes 12 3 0.13907285 0.59649123

958 fish_Ammodytes 12 3 0.11920530 0.56140351

959 fish_Ammodytes 12 3 0.10596026 0.52631579

960 fish_Ammodytes 12 3 0.08609272 0.42105263

961 fish_Ammodytes 12 3 0.07947020 0.42105263

962 fish_Ammodytes 12 3 0.06622517 0.35087719

963 fish_Ammodytes 12 3 0.00000000 0.00000000

964 fish_Ammodytes 12 4 1.00000000 1.00000000

965 fish_Ammodytes 12 4 0.96666667 0.94285714

966 fish_Ammodytes 12 4 0.93333333 0.87142857

967 fish_Ammodytes 12 4 0.90000000 0.95714286

968 fish_Ammodytes 12 4 0.86666667 0.82857143

969 fish_Ammodytes 12 4 0.83333333 0.92857143

970 fish_Ammodytes 12 4 0.80000000 0.82857143

971 fish_Ammodytes 12 4 0.77333333 0.85714286

972 fish_Ammodytes 12 4 0.74666667 0.75714286

973 fish_Ammodytes 12 4 0.72000000 0.85714286

974 fish_Ammodytes 12 4 0.69333333 0.85714286

975 fish_Ammodytes 12 4 0.66666667 0.77142857

976 fish_Ammodytes 12 4 0.64000000 0.77142857

977 fish_Ammodytes 12 4 0.60666667 0.87142857

978 fish_Ammodytes 12 4 0.58000000 0.75714286

979 fish_Ammodytes 12 4 0.56000000 0.84285714

980 fish_Ammodytes 12 4 0.52666667 0.77142857

981 fish_Ammodytes 12 4 0.50000000 0.88571429

982 fish_Ammodytes 12 4 0.47333333 0.75714286

983 fish_Ammodytes 12 4 0.44666667 0.78571429

984 fish_Ammodytes 12 4 0.42000000 0.75714286

985 fish_Ammodytes 12 4 0.39333333 0.80000000

986 fish_Ammodytes 12 4 0.36666667 0.77142857

987 fish_Ammodytes 12 4 0.34666667 0.77142857

988 fish_Ammodytes 12 4 0.32000000 0.85714286

989 fish_Ammodytes 12 4 0.29333333 0.78571429

990 fish_Ammodytes 12 4 0.26666667 0.82857143

991 fish_Ammodytes 12 4 0.24666667 0.80000000

992 fish_Ammodytes 12 4 0.22000000 0.77142857

993 fish_Ammodytes 12 4 0.19333333 0.88571429

994 fish_Ammodytes 12 4 0.17333333 0.74285714

995 fish_Ammodytes 12 4 0.15333333 0.77142857

996 fish_Ammodytes 12 4 0.12666667 0.67142857

997 fish_Ammodytes 12 4 0.11333333 0.67142857

998 fish_Ammodytes 12 4 0.09333333 0.67142857

999 fish_Ammodytes 12 4 0.08666667 0.54285714

1000 fish_Ammodytes 12 4 0.08000000 0.60000000

1001 fish_Ammodytes 12 4 0.06666667 0.54285714

1002 fish_Ammodytes 12 4 0.00000000 0.00000000

1003 fish_Ammodytes 12 5 1.00000000 1.00000000

1004 fish_Ammodytes 12 5 0.93661972 1.00000000

1005 fish_Ammodytes 12 5 0.88028169 0.97560976

1006 fish_Ammodytes 12 5 0.82394366 0.97560976

1007 fish_Ammodytes 12 5 0.76760563 0.92682927

1008 fish_Ammodytes 12 5 0.71830986 0.90243902

1009 fish_Ammodytes 12 5 0.66197183 0.97560976

1010 fish_Ammodytes 12 5 0.60563380 0.90243902

1011 fish_Ammodytes 12 5 0.55633803 0.90243902

1012 fish_Ammodytes 12 5 0.50704225 0.87804878

1013 fish_Ammodytes 12 5 0.45774648 0.95121951

1014 fish_Ammodytes 12 5 0.40845070 0.85365854

1015 fish_Ammodytes 12 5 0.36619718 0.87804878

1016 fish_Ammodytes 12 5 0.30985915 0.82926829

1017 fish_Ammodytes 12 5 0.26760563 0.85365854

1018 fish_Ammodytes 12 5 0.23239437 0.80487805

1019 fish_Ammodytes 12 5 0.19014085 0.78048781

1020 fish_Ammodytes 12 5 0.14788732 0.75609756

1021 fish_Ammodytes 12 5 0.11267606 0.58536585

1022 fish_Ammodytes 12 5 0.10563380 0.53658537

1023 fish_Ammodytes 12 5 0.09154930 0.43902439

1024 fish_Ammodytes 12 5 0.07042253 0.41463415

1025 fish_Ammodytes 12 5 0.06338028 0.31707317

1026 fish_Ammodytes 12 5 0.05633803 0.29268293

1027 fish_Ammodytes 12 5 0.04225352 0.21951219

1028 fish_Ammodytes 12 5 0.03521127 0.17073171

1029 fish_Ammodytes 12 5 0.02816901 0.12195122

1030 fish_Ammodytes 12 5 0.02112676 0.07317073

1031 fish_Ammodytes 12 5 0.00000000 0.00000000

1032 fish_Ammodytes 12 6 1.00000000 0.82857143

1033 fish_Ammodytes 12 6 0.97972973 0.91428571

1034 fish_Ammodytes 12 6 0.95270270 0.92857143

1035 fish_Ammodytes 12 6 0.91891892 0.94285714

1036 fish_Ammodytes 12 6 0.89189189 0.88571429

1037 fish_Ammodytes 12 6 0.85810811 0.95714286

1038 fish_Ammodytes 12 6 0.83783784 0.91428571

1039 fish_Ammodytes 12 6 0.80405405 0.91428571

1040 fish_Ammodytes 12 6 0.78378378 0.92857143

1041 fish_Ammodytes 12 6 0.75675676 0.92857143

1042 fish_Ammodytes 12 6 0.72972973 0.94285714

1043 fish_Ammodytes 12 6 0.70270270 1.00000000

1044 fish_Ammodytes 12 6 0.66891892 0.95714286

1045 fish_Ammodytes 12 6 0.64189189 0.95714286

1046 fish_Ammodytes 12 6 0.62162162 0.88571429

1047 fish_Ammodytes 12 6 0.58783784 0.92857143

1048 fish_Ammodytes 12 6 0.56081081 0.94285714

1049 fish_Ammodytes 12 6 0.53378378 0.94285714

1050 fish_Ammodytes 12 6 0.50675676 0.94285714

1051 fish_Ammodytes 12 6 0.48648649 0.91428571

1052 fish_Ammodytes 12 6 0.45270270 0.95714286

1053 fish_Ammodytes 12 6 0.43243243 0.95714286

1054 fish_Ammodytes 12 6 0.40540540 0.97142857

1055 fish_Ammodytes 12 6 0.37837838 0.98571429

1056 fish_Ammodytes 12 6 0.35135135 0.98571429

1057 fish_Ammodytes 12 6 0.33108108 0.90000000

1058 fish_Ammodytes 12 6 0.30405405 0.90000000

1059 fish_Ammodytes 12 6 0.28378378 0.91428571

1060 fish_Ammodytes 12 6 0.25675676 0.87142857

1061 fish_Ammodytes 12 6 0.23648649 0.84285714

1062 fish_Ammodytes 12 6 0.21621622 0.71428571

1063 fish_Ammodytes 12 6 0.20270270 0.71428571

1064 fish_Ammodytes 12 6 0.18243243 0.67142857

1065 fish_Ammodytes 12 6 0.16891892 0.60000000

1066 fish_Ammodytes 12 6 0.15540540 0.55714286

1067 fish_Ammodytes 12 6 0.14864865 0.55714286

1068 fish_Ammodytes 12 6 0.12837838 0.51428571

1069 fish_Ammodytes 12 6 0.10810811 0.51428571

1070 fish_Ammodytes 12 6 0.00000000 0.00000000

1071 fish_Ammodytes 12 7 1.00000000 1.00000000

1072 fish_Ammodytes 12 7 0.94666667 0.63157895

1073 fish_Ammodytes 12 7 0.89333333 0.73684211

1074 fish_Ammodytes 12 7 0.84000000 0.81578947

1075 fish_Ammodytes 12 7 0.78666667 0.73684211

1076 fish_Ammodytes 12 7 0.74000000 0.76315789

1077 fish_Ammodytes 12 7 0.69333333 0.71052632

1078 fish_Ammodytes 12 7 0.61333333 0.73684211

1079 fish_Ammodytes 12 7 0.53333333 0.71052632

1080 fish_Ammodytes 12 7 0.48666667 0.68421053

1081 fish_Ammodytes 12 7 0.44666667 0.78947368

1082 fish_Ammodytes 12 7 0.38666667 0.71052632

1083 fish_Ammodytes 12 7 0.34666667 0.73684211

1084 fish_Ammodytes 12 7 0.30000000 0.68421053

1085 fish_Ammodytes 12 7 0.25333333 0.71052632

1086 fish_Ammodytes 12 7 0.21333333 0.71052632

1087 fish_Ammodytes 12 7 0.16666667 0.65789474

1088 fish_Ammodytes 12 7 0.13333333 0.52631579

1089 fish_Ammodytes 12 7 0.10000000 0.42105263

1090 fish_Ammodytes 12 7 0.08000000 0.34210526

1091 fish_Ammodytes 12 7 0.00000000 0.00000000

1092 fish_Ammodytes 12 8 1.00000000 0.88679245

1093 fish_Ammodytes 12 8 0.95333333 0.86792453

1094 fish_Ammodytes 12 8 0.90000000 0.90566038

1095 fish_Ammodytes 12 8 0.85333333 0.92452830

1096 fish_Ammodytes 12 8 0.81333333 0.86792453

1097 fish_Ammodytes 12 8 0.76000000 0.92452830

1098 fish_Ammodytes 12 8 0.71333333 0.94339623

1099 fish_Ammodytes 12 8 0.66666667 0.86792453

1100 fish_Ammodytes 12 8 0.62666667 0.81132076

1101 fish_Ammodytes 12 8 0.58666667 1.00000000

1102 fish_Ammodytes 12 8 0.54666667 0.88679245

1103 fish_Ammodytes 12 8 0.50000000 0.86792453

1104 fish_Ammodytes 12 8 0.46000000 0.83018868

1105 fish_Ammodytes 12 8 0.42000000 1.00000000

1106 fish_Ammodytes 12 8 0.37333333 0.98113207

1107 fish_Ammodytes 12 8 0.33333333 0.96226415

1108 fish_Ammodytes 12 8 0.29333333 0.94339623

1109 fish_Ammodytes 12 8 0.25333333 0.90566038

1110 fish_Ammodytes 12 8 0.22000000 0.88679245

1111 fish_Ammodytes 12 8 0.18666667 0.77358491

1112 fish_Ammodytes 12 8 0.16000000 0.67924528

1113 fish_Ammodytes 12 8 0.13333333 0.56603774

1114 fish_Ammodytes 12 8 0.12000000 0.47169811

1115 fish_Ammodytes 12 8 0.10000000 0.43396226

1116 fish_Ammodytes 12 8 0.08666667 0.41509434

1117 fish_Ammodytes 12 8 0.00000000 0.00000000

1118 fish_Ammodytes 12 9 1.00000000 0.92424242

1119 fish_Ammodytes 12 9 0.94573643 1.00000000

1120 fish_Ammodytes 12 9 0.88372093 0.93939394

1121 fish_Ammodytes 12 9 0.82170543 0.95454545

1122 fish_Ammodytes 12 9 0.75968992 0.96969697

1123 fish_Ammodytes 12 9 0.69767442 0.95454545

1124 fish_Ammodytes 12 9 0.64341085 1.00000000

1125 fish_Ammodytes 12 9 0.58914729 0.96969697

1126 fish_Ammodytes 12 9 0.52713178 1.00000000

1127 fish_Ammodytes 12 9 0.47286822 0.98484848

1128 fish_Ammodytes 12 9 0.41085271 1.00000000

1129 fish_Ammodytes 12 9 0.36434109 0.98484848

1130 fish_Ammodytes 12 9 0.30232558 1.00000000

1131 fish_Ammodytes 12 9 0.25581395 0.98484848

1132 fish_Ammodytes 12 9 0.21705426 1.00000000

1133 fish_Ammodytes 12 9 0.18604651 0.86363636

1134 fish_Ammodytes 12 9 0.17054264 0.86363636

1135 fish_Ammodytes 12 9 0.14728682 0.77272727

1136 fish_Ammodytes 12 9 0.13178295 0.77272727

1137 fish_Ammodytes 12 9 0.11627907 0.66666667

1138 fish_Ammodytes 12 9 0.10077519 0.57575758

1139 fish_Ammodytes 12 9 0.08527132 0.53030303

1140 fish_Ammodytes 12 9 0.06976744 0.48484848

1141 fish_Ammodytes 12 9 0.05426357 0.39393939

1142 fish_Ammodytes 12 9 0.00000000 0.00000000

1143 Pleurobrachia_bachei 13 1 1.00000000 1.00000000

1144 Pleurobrachia_bachei 13 1 0.94000000 1.00000000

1145 Pleurobrachia_bachei 13 1 0.88100000 0.88800000

1146 Pleurobrachia_bachei 13 1 0.82100000 0.78600000

1147 Pleurobrachia_bachei 13 1 0.76100000 0.78600000

1148 Pleurobrachia_bachei 13 1 0.70100000 0.78600000

1149 Pleurobrachia_bachei 13 1 0.64200000 0.78600000

1150 Pleurobrachia_bachei 13 1 0.58200000 0.78600000

1151 Pleurobrachia_bachei 13 1 0.52200000 0.82700000

1152 Pleurobrachia_bachei 13 1 0.46300000 0.82700000

1153 Pleurobrachia_bachei 13 1 0.41000000 0.82700000

1154 Pleurobrachia_bachei 13 1 0.35100000 0.74500000

1155 Pleurobrachia_bachei 13 1 0.29900000 0.65300000

1156 Pleurobrachia_bachei 13 1 0.23900000 0.65300000

1157 Pleurobrachia_bachei 13 1 0.17900000 0.65300000

1158 Pleurobrachia_bachei 13 1 0.11900000 0.65300000

1159 Pleurobrachia_bachei 13 1 0.03700000 0.46900000

1160 Pleurobrachia_bachei 13 1 0.00000000 0.00000000

1161 Mnemiopsis_leidyi 14 1 1.00000000 1.00000000

1162 Mnemiopsis_leidyi 14 1 0.93500000 1.00000000

1163 Mnemiopsis_leidyi 14 1 0.87700000 1.00000000

1164 Mnemiopsis_leidyi 14 1 0.82600000 0.95500000

1165 Mnemiopsis_leidyi 14 1 0.77500000 0.95500000

1166 Mnemiopsis_leidyi 14 1 0.70300000 0.85700000

1167 Mnemiopsis_leidyi 14 1 0.64500000 0.85700000

1168 Mnemiopsis_leidyi 14 1 0.58000000 0.85700000

1169 Mnemiopsis_leidyi 14 1 0.52200000 0.82700000

1170 Mnemiopsis_leidyi 14 1 0.46400000 0.82700000

1171 Mnemiopsis_leidyi 14 1 0.41300000 0.82700000

1172 Mnemiopsis_leidyi 14 1 0.34900000 0.82700000

1173 Mnemiopsis_leidyi 14 1 0.29000000 0.82700000

1174 Mnemiopsis_leidyi 14 1 0.23200000 0.82700000

1175 Mnemiopsis_leidyi 14 1 0.17400000 0.82700000

1176 Mnemiopsis_leidyi 14 1 0.11600000 0.84200000

1177 Mnemiopsis_leidyi 14 1 0.05800000 0.69200000

1178 Mnemiopsis_leidyi 14 1 0.02200000 0.41400000

1179 Mnemiopsis_leidyi 14 1 0.00000000 0.00000000

1180 Bolinopsis_infundibulum 15 1 1.00000000 1.00000000

1181 Bolinopsis_infundibulum 15 1 0.94400000 1.00000000

1182 Bolinopsis_infundibulum 15 1 0.88700000 0.90200000

1183 Bolinopsis_infundibulum 15 1 0.83100000 0.94700000

1184 Bolinopsis_infundibulum 15 1 0.77500000 0.94700000

1185 Bolinopsis_infundibulum 15 1 0.71800000 0.94700000

1186 Bolinopsis_infundibulum 15 1 0.66200000 0.94700000

1187 Bolinopsis_infundibulum 15 1 0.60600000 0.94700000

1188 Bolinopsis_infundibulum 15 1 0.54900000 0.94700000

1189 Bolinopsis_infundibulum 15 1 0.49300000 0.94000000

1190 Bolinopsis_infundibulum 15 1 0.44400000 0.86500000

1191 Bolinopsis_infundibulum 15 1 0.38700000 0.91000000

1192 Bolinopsis_infundibulum 15 1 0.33800000 0.86500000

1193 Bolinopsis_infundibulum 15 1 0.28200000 0.90200000

1194 Bolinopsis_infundibulum 15 1 0.22500000 0.94000000

1195 Bolinopsis_infundibulum 15 1 0.17600000 0.92500000

1196 Bolinopsis_infundibulum 15 1 0.11300000 0.74400000

1197 Bolinopsis_infundibulum 15 1 0.07700000 0.62400000

1198 Bolinopsis_infundibulum 15 1 0.02100000 0.54100000

1199 Bolinopsis_infundibulum 15 1 0.00000000 0.00000000

1200 Ciona_intestinalis 16 1 1.00000000 1.00000000

1201 Ciona_intestinalis 16 1 0.88900000 0.83800000

1202 Ciona_intestinalis 16 1 0.77800000 0.83800000

1203 Ciona_intestinalis 16 1 0.66700000 0.83800000

1204 Ciona_intestinalis 16 1 0.55600000 0.76900000

1205 Ciona_intestinalis 16 1 0.44400000 0.58100000

1206 Ciona_intestinalis 16 1 0.33300000 0.41900000

1207 Ciona_intestinalis 16 1 0.22200000 0.36300000

1208 Ciona_intestinalis 16 1 0.11100000 0.31300000

1209 Ciona_intestinalis 16 1 0.05500000 0.20600000

1210 Ciona_intestinalis 16 1 0.00000000 0.00000000

1211 Ciona_intestinalis 16 2 1.00000000 1.00000000

1212 Ciona_intestinalis 16 2 0.88900000 0.83700000

1213 Ciona_intestinalis 16 2 0.77800000 0.79300000

1214 Ciona_intestinalis 16 2 0.66700000 0.75500000

1215 Ciona_intestinalis 16 2 0.55600000 0.56500000

1216 Ciona_intestinalis 16 2 0.44400000 0.56500000

1217 Ciona_intestinalis 16 2 0.33300000 0.55300000

1218 Ciona_intestinalis 16 2 0.22200000 0.46200000

1219 Ciona_intestinalis 16 2 0.11100000 0.36400000

1220 Ciona_intestinalis 16 2 0.05500000 0.22800000

1221 Ciona_intestinalis 16 2 0.00000000 0.00000000

1222 Ciona_intestinalis 16 3 1.00000000 1.00000000

1223 Ciona_intestinalis 16 3 0.88900000 0.80600000

1224 Ciona_intestinalis 16 3 0.77800000 0.80600000

1225 Ciona_intestinalis 16 3 0.66700000 0.54800000

1226 Ciona_intestinalis 16 3 0.55600000 0.43500000

1227 Ciona_intestinalis 16 3 0.44400000 0.41900000

1228 Ciona_intestinalis 16 3 0.33300000 0.38700000

1229 Ciona_intestinalis 16 3 0.22200000 0.33900000

1230 Ciona_intestinalis 16 3 0.11100000 0.32300000

1231 Ciona_intestinalis 16 3 0.05500000 0.27400000

1232 Ciona_intestinalis 16 3 0.00000000 0.00000000

1233 Ciona_intestinalis 16 4 1.00000000 1.00000000

1234 Ciona_intestinalis 16 4 0.88900000 1.00000000

1235 Ciona_intestinalis 16 4 0.77800000 0.91500000

1236 Ciona_intestinalis 16 4 0.66700000 0.81700000

1237 Ciona_intestinalis 16 4 0.55600000 0.70400000

1238 Ciona_intestinalis 16 4 0.44400000 0.70400000

1239 Ciona_intestinalis 16 4 0.33300000 0.63400000

1240 Ciona_intestinalis 16 4 0.22200000 0.43700000

1241 Ciona_intestinalis 16 4 0.11100000 0.40800000

1242 Ciona_intestinalis 16 4 0.05500000 0.36600000

1243 Ciona_intestinalis 16 4 0.00000000 0.00000000

1244 Ciona_intestinalis 16 5 1.00000000 1.00000000

1245 Ciona_intestinalis 16 5 0.88900000 0.79000000

1246 Ciona_intestinalis 16 5 0.77800000 0.76500000

1247 Ciona_intestinalis 16 5 0.66700000 0.67900000

1248 Ciona_intestinalis 16 5 0.55600000 0.67900000

1249 Ciona_intestinalis 16 5 0.44400000 0.67900000

1250 Ciona_intestinalis 16 5 0.33300000 0.65400000

1251 Ciona_intestinalis 16 5 0.22200000 0.60500000

1252 Ciona_intestinalis 16 5 0.11100000 0.43200000

1253 Ciona_intestinalis 16 5 0.05500000 0.29600000

1254 Ciona_intestinalis 16 5 0.00000000 0.00000000

1255 Ciona_intestinalis 16 6 1.00000000 1.00000000

1256 Ciona_intestinalis 16 6 0.88900000 0.89300000

1257 Ciona_intestinalis 16 6 0.77800000 0.84000000

1258 Ciona_intestinalis 16 6 0.66700000 0.82400000

1259 Ciona_intestinalis 16 6 0.55600000 0.74800000

1260 Ciona_intestinalis 16 6 0.44400000 0.73300000

1261 Ciona_intestinalis 16 6 0.33300000 0.57300000

1262 Ciona_intestinalis 16 6 0.22200000 0.40500000

1263 Ciona_intestinalis 16 6 0.11100000 0.30500000

1264 Ciona_intestinalis 16 6 0.05500000 0.22100000

1265 Ciona_intestinalis 16 6 0.00000000 0.00000000
